# Title: Cytokine release syndrome is not usually caused by secondary hemophagocytic lymphohistiocytosis in a cohort of 19 critically ill COVID-19 patients

**DOI:** 10.1038/s41598-020-75260-w

**Published:** 2020-10-26

**Authors:** Georg Lorenz, Philipp Moog, Quirin Bachmann, Paul La Rosée, Heike Schneider, Michaela Schlegl, Christoph Spinner, Uwe Heemann, Roland M. Schmid, Hana Algül, Tobias Lahmer, Wolfgang Huber, Christoph Schmaderer

**Affiliations:** 1 School of Medicine, Klinikum rechts der Isar, Department of Nephrology, Technical University of Munich, Ismaninger Str. 22, 81675 Munich, Germany; 2 Clinic for Internal Medicine II, Schwarzwald-Baar Klinikum Villingen-Schwenningen, Klinikstr. 11, 78052 Villingen-Schwenningen, Germany; 3 School of Medicine, Klinikum rechts der Isar, Department for Clinical Chemistry, Technical University of Munich, Ismaninger Str. 22, 81675 Munich, Germany; 4 School of Medicine, Klinikum rechts der Isar, II. Department for Internal Medicine, Technical University of Munich, Ismaninger Str. 22, 81675 Munich, Germany; 5 Comprehensive Cancer Center Munich at the Klinikum rechts der Isar, Technische Universität München, 81675 Munich, Germany; 6 German Center for Infectious Research (DZIF), Technische Universität München, 81675 Munich, Germany; 7 School of Medicine, Klinikum rechts der Isar, Division of Rheumatology, Ismaninger Straße 22, 81675 Munich, Germany

**Keywords:** Immunology, Cytokines, Infection, Inflammation, Innate immune cells, Innate immunity

## Abstract

Severe COVID-19 associated respiratory failure, poses the one challenge of our days. Assessment and treatment of COVID-19 associated hyperinflammation may be key to improve outcomes. It was speculated that in subgroups of patients secondary hemophagocytic lymphohistiocytosis (sHLH) or cytokine release syndrome (CRS) with features of macrophage activation syndrome might drive severe disease trajectories. If confirmed, profound immunosuppressive therapy would be a rationale treatment approach. Over a median observation period of 11 (IQR: 8; 16) days, 19 consecutive confirmed severe COVID-19-patients admitted to our intensive-care-unit were tested for presence of sHLH by two independent experts. HScores and 2004-HLH diagnostic criteria were assessed. Patients were grouped according to short-term clinical courses: discharge from ICU versus ongoing ARDS or death at time of analysis. The median HScore at admission was 157 (IQR: 98;180), without the key clinical triad of HLH, i.e. progressive cytopenia, persistent fever and organomegaly. Independent expert chart review revealed the absence of sHLH in all cases. No patient reached more than 3/6 of modified HLH 2004 criteria. Nevertheless, patients presented hyperinflammation with peripheral neutrophilic signatures (neutrophil/lymphocyte-ratio > 3.5). The latter best paralleled their short-term clinical courses, with declining relative neutrophil numbers prior to extubation (4.4, [IQR: 2.5;6.3]; n = 8) versus those with unfavourable courses (7.6, [IQR: 5.2;31], n = 9). Our study rules out virus induced sHLH as the leading cause of most severe-COVID-19 trajectories. Instead, an associated innate neutrophilic hyperinflammatory response or virus-associated-CRS appears dominant in patients with an unfavourable clinical course. Therapeutic implications are discussed.

## Introduction

The SARS-CoV-2 globally poses medical and economic challenges. While epidemiologists and governments are buying time and capacity through various strategies, it is up to clinicians to optimize the treatment of severe COVID-19 ^1,2^. Various anti-viral drugs, including the polymerase-, or protease-inhibitors and passive immunization attempts are currently undergoing clinical trial ^3–5^.

Yet, in cases of serious COVID-19 with pneumonia and acute respiratory distress syndrome (ARDS), tocilizumab, a humanized monoclonal Interleukin-6-receptor antagonist, is administered in a phase II open label clinical trial (NCT04317092) to supress hyperinflammation that is assumed to cause fatal lung and multiorgan injury ^6,7^. This is supported by cohort data from China, which report increased ferritin, and elevated TNF-α, IL-6 and IFN-γ levels in severe versus mild COVID-19-cases. Based on these preliminary data, cytokine release syndrome (CRS) or virus induced adult secondary hemophagocytic lymphohistocytosis (sHLH) have been proposed as underlying aetiology of severe COVID-19 ^7,8^. The latter is a clinical syndrome, characterized by massive systemic inflammation, persistent fever flares, hepatosplenomegaly, and severe cytopenia, which is rooted in hemophagocytic activity in bone marrow. It is a rare and often fatal clinical syndrome which can be observed in the context of malignancy, systemic autoimmunity, and viral infections such as Epstein-Barr virus ^9^. Full blown sHLH in the latter context usually requires cytotoxic agents, i.e. etoposide, which is not to be used carelessly ^9^.

The term CRS originally defines drug toxicity, which can be observed subsequently to monoclonal antibody or adoptive T-cell therapies. The syndrome is rooted in massive aberrant B-, T-cell and monocyte activation and a systemic “cytokine storm”. The clinical course is pleiotropic, with fever, skin rash, cytopenia, neurologic symptoms, coagulopathy, hypotension and eventually organ failure i.e. ARDS. Application of tocilizumab usually resolves symptoms ^10,11^. In the sense of an exuberant anti-viral innate immune response, elevated cytokine levels, fever and CRS-like symptoms, including organ injury have been reported in the context of viral infections, such as H5N1, SARS-CoV-1 and SARS-CoV-2 ^14,1213^. To avoid confusion, we will refer to this with the term “virus-associated CRS”.

Whether “virus-associated” CRS, sHLH or both are present in severe COVID-19 profoundly impacts our understanding of the disease and impacts on therapeutic strategies. We therefore screened all consecutive 19 severe COVID-19-patients, who were admitted to our intensive care unit (ICU) for (a) CRS and (b) presence of sHLH, using the HScore, and 2004-HLH-diagnostic-criteria. Charts were reviewed by two independent HLH-experienced clinicians to rule in or out actual sHLH.

## Results

### Baseline characteristics, treatment and clinical course of severe COVID-19-cases

19 consecutive laboratory confirmed COVID-19-patients with typical chest X-ray (n = 1) or CT-scan (n = 18) were admitted to our ICU ^15,16^. Overall, they were mostly male (89%), with a median age of 70 (IQR: 58; 78) years. All had at least one pre-existing comorbidity or history of organ failure, with arterial hypertension (58%) being the most frequent comorbidity.

Median time from hospital admission to ICU-transferral, time from ICU-admission to oral intubation (n = 17) and median time of observation were 3 (IQR: 0;4), 0 (IQR: 0;0), 11 (IQR: 8;16) days, respectively. Median SOFA-Score and worst Horovitz index were 5 (IQR: 3;7) and 180 (IQR: 125;200) mmHg, respectively. Additional incidence of organ failure other than pulmonary can be retrieved from Table 1.

**Table 1:** Cohort characteristics at time of ICU-admission and clinical course.

Parameter	Overall (n = 19)	Favourable (n = 9)	Unfavourable course (n = 10)	
	Median (IQR)/Count (% of group)			*p*-value
Age	70 (58; 78)	59 (53; 73)	75 (60; 83)	**0.049**
Male sex no. (%)	17 (89%)	8 (89%)	9 (90%)	0.94
BMI	26 (24, 32)	25 (22; 37)	26 (24; 31)	0.48
Obesity (BMI ≥ 30)	6 (32%)	3 (33%)	3 (33%)	0.91
Comorbidity (sum) *	2 (2; 3)	2 (2; 4)	3 (2;3)	0.54
Pulmonary	5 (26%)	2 (22%)	3 (30%)	0.78
CVD	4 (21%)	0 (0%)	4 (40%)	0.16
AF	5 (26%)	2 (22%)	3 (30%)	0.78
Hypertension	11 (58%)	6 (67%)	5 (50%)	0.55
History of malignancy	2 (11%)	2 (22%)	0 (0%)	0.45
History of organ failure / sepsis	4 (21%)	2 (22%)	2 (20%)	0.97
Diabetes	5 (26%)	2 (22%)	3 (30%)	0.78
Liver disease	2 (11%)	1 (11%)	1 (10%)	0.97
Auto-immune/-inflammatory disease **	1 (5%)	1 (11%)	0 (0%)	0.72
Neurologic disorder / dementia	4 (21%)	2 (22%)	2 (20%)	0.97
CKD Stage III-IV	4 (21%)	1 (11%)	3 (30%)	0.50
Prior Immunosuppression ***	2 (11%)	2 (22%)	0 (0%)	0.45
SOFA-Score at time of ICU-admission	5 (3; 7)	4 (3; 6)	5.5 (3.5; 8)	0.34
**Clinical course**				
Time of observation	11 (8; 16)	11 (8;14)	12 (8; 16)	0.55
Time admission to ICU-admission [d]	3 (0; 4)	1 (0.5; 7)	3 (0; 3)	0.50
Time ICU-admission to intubation [d]	0 (0; 0)	0 (0; 0.8)	0 (0; 0)	0.67
Days of mechanical ventilation	11 (8; 16)	8.5 (8; 14)	15 (8; 17)	0.42
N requiring intubation	17 (89%)	7 (78%)	10 (100%)	0.13
Horovitz index (worst) mmHg	180 (100; 200)	200 (165; 200)	150 (74; 200)	**0.04**
Bilirubin [mg/dl] – max	0.8 (0.7; 1.8)	0.8 (0.7; 1.5)	1 (0.6, 6.2)	0.14
Creatinine [mg/dl] – max	1.6 (1.3; 2.2)	1.4 (1.3; 1.9)	1.9 (1.4; 3.9)	0.12
AST [U/l] – max	130 (69; 248)	108 (65; 222)	130 (71; 284)	0.37
Noradrenalin initial.[µg/h]	600 (0; 2000)	600 (200; 1100)	1150 (0; 2000)	0.77
Noradrenalin max.[µg/h]	700 (250; 2000)	600 (325; 1100)	1250 (0; 2000)	0.62
**Concomitant organ failure**				
AKI	9 (47%)	2 (22%)	7 (70%)	**0.04**
Liver failure	6 (32%)	0 (0%)	6 (60%)	**0.01**
De novo seizure	2 (11%)	0 (0%)	2 (20%)	0.17
Organ support (SLED/ADVOS/ECMO)	7 (37%)	1 (11%)	6 (60%)	**0.03**
Deceased patients	3 (16%)	n.a	3 (30%)	n.a

(Sub)groups: “Overall”, “favourable” versus “unfavourable” clinical group. Statistical comparisons were done using ANOVA-, Mann–Whitney- and Kruskal–Wallis-test as appropriate comparing “favourable” versus “unfavourable” clinical groups. *Comorbidities were defined as follows: Pulmonary disease: Asthma, chronic obstructive pulmonary disease or fibrotic lung disease; Cardiovascular disease (CVD): Pulmonary artery embolism, peripheral arterial occlusive disease, history of lung oedema; Atrial fibrillation (AF); Liver disease: history of gastrointestinal bleeding, liver-cirrhosis or pancreatitis;** Ulcerative colitis; ***prior immunosuppression included recent history of stem-cell transplantation, intake of cyclosporine and low dose steroids. Abbreviation: Advanced organ support (ADVOS), acute kidney injury – transient / requiring dialysis (AKI), Aspartate-aminotransferase (AST), chronic kidney disease (CKD), extracorporeal membrane oxygenation (ECMO), not applicable (n.a.), sustained low efficiency dialysis (SLED), Sepsis-related organ failure assessment score (SOFA).

Patients were treated in accordance with the “surviving sepsis campaign guidelines for severe COVID-19” ^17^. All patients received intravenous broad-spectrum antibiotics and regular surveillance for superinfection. Two patients additionally received remdesivir within clinical trials. Most patients (89%) showed rapid deterioration of oxygenation and were intubated after a median of 0 (IQR: 0;0) days.

**Figure 1:**
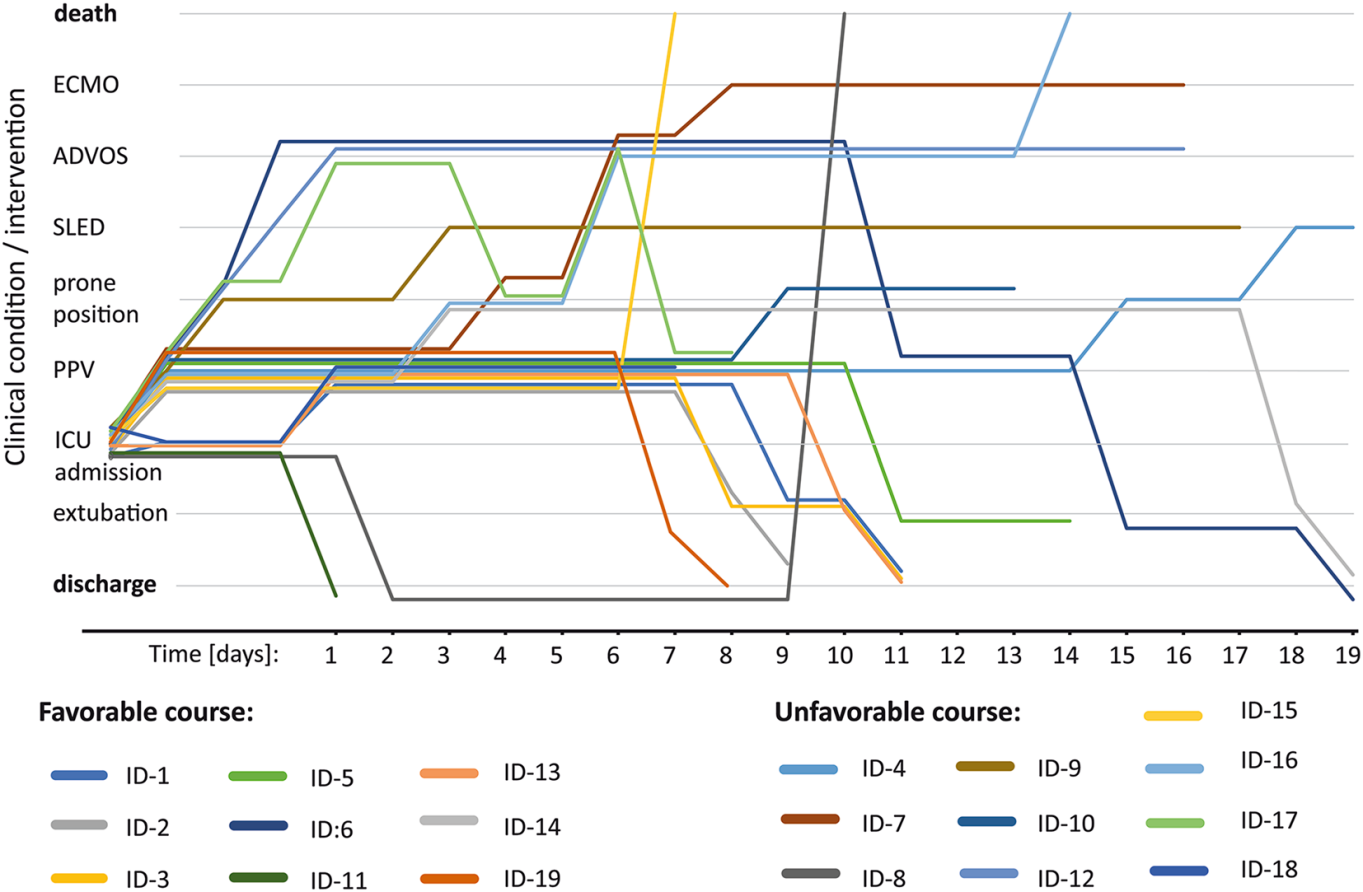
Depicts individual’s clinical courses. Time from ICU-admission in days is displayed on the x-axis. Upward vertical movement indicates worsening of the clinical condition (up: intubation + positive pressure ventilation (PPV), requiring prone positioning, requiring sustained low efficiency dialysis (SLED), advanced organ support (ADVOS = dialysis + albumin-dialysis + CO2 removal) or extracorporeal membrane oxygenation (ECMO = oxygenation + CO2-removal). Downward vertical movement indicates reduced requirement of organ support, spontaneous breathing and discharge from ICU in good clinical condition.

Our approach resulted in clinical improvement of 47% of patients. After a median of 8.5 (IQR 8;12.5) days, eight patients were extubated, one patient was discharged without need for mechanical ventilation. The remaining cases were judged as unfavourable, due to prolonged weaning, progressive ARDS requiring organ support (n = 7), or death (n = 3) (see Fig. 1). No group differences (favourable versus unfavourable group) were detected with respect to time of observation, comorbidities, SOFA-Score (5.5 [IQR: 3.5;7.8] versus 4 [IQR: 3;6], *p* = 0.34) or initial noradrenalin dose (1150 [IQR: 0;2000] versus 600 [IQR: 200;1100] µg/h, *p* = 0.77). Unfavourable clinical courses were associated with older age (75 [IQR: 60;83] versus 59 [IQR: 53;73] a, *p* = 0,049) and were observed among both men and women. Yet, higher Horovitz index (200 [IQR: 165;200] versus 150 [IQR: 74;200] mmHg, *p* = 0.04) was recorded in the “favourable group” during the observation period. Kidney injury (70% versus 22%, *p* = 0.04), liver failure (60% versus 0%, *p* = 0.01) and need for organ support were more likely in those who took an unfavourable clinical course (60% versus 11%, *p* = 0.03). Of note, requirement of organ support did not per se preclude a favourable course (Fig. 1 – ID-6). For more details see Table 1 and Fig. 1.

### Immunologic features and relation to clinical course in severe COVID-19

Most patients showed an hyperinflammatory immune response while the pulmonary gas exchange deteriorated. At ICU-admission, most had fever (38.2 (IQR: 37.6;39)°C), all presented with increased C-reactive-protein-, elevated IL-6-, serum ferritin- and sIL-2R- with relatively low procalcitonin-levels (CRP: 20.4 [IQR: 14.4;26.3] mg/dl; IL-6: 139 [IQR: 85.8;872] pg/ml; ferritin: 1815, [IQR: 863;3674] ng/ml; sIL-2R: 1404 [IQR: 1097;2843] IU/ml; PCT: 0.8 [IQR: 0.3;3.3] ng/ml, respectively). All patients showed hyper-fibrinogenemia (777 [IQR: 654;859] mg/dl). Splenomegaly (21%) and hepatomegaly (26%) were found in the minority of patients (Table 2).

**Table 2:** Short-term trends of immunologic parameters after ICU-admission.

Parameter	Overall (n = 19) ICU-ADM	Overall (n = 19) 1 week*	*p*-value (pairwise)	Favourable (n = 9)	Unfavourable Group (ADM) (n = 10)	*p*-value (indep.)
	Median (IQR) / Count (%)			Median (IQR) / Count (%)		
Body Temperature[°C]	38.2 (37.6; 39)	37.8 (37.3; 38.2)	0.20	38.5 (37.7; 38.7)	38.1 (37.4; 39.5)	0.70
Splenomegaly (1 = yes)	4 (21%)	n.a	n.a	2 (22%)	2 (20%)	0.97
Hepatomegaly (1 = yes)	5 (26%)	n.a	n.a	2 (22%)	3 (30%)	0.78
CRP [mg/dl]	20.4 (14.4; 26.3)	14.3 (8.1; 27.3)	0.14	21.3 (18.6; 24.7)	17.5(12.3; 27.9)	0.57
IL-6 [pg/ml]	139 (85.8; 872)	68 (38.5; 615,5)	0.379	90.2(64.8; 119.5)	568 (161; 2094)	0.14
PCT [ng/ml]	0.8 (0.3; 3.3)	0.4 (0.2; 1.1)	0.24	0.4 (0.2; 3.1)	1.0 (0.5; 3.4)	0.81
N (PCT ≥ 1 ng/ml)	n.a	8 (42%)	n.a	1 (11%)	7 (70%)	0.18
N (culture positive)	n.a	6 (32%)	n.a	1 (11%)	5 (50%)	0.32
D-Dimer [µg/l]	2961 (1498; 6548)	6492(3128;9618)	0.37	2961(1728;7689)	2493(1428;6410)	0.80
Fibrinogen[mg/dl]	777 (654; 859)	646 (594; 811)	**0.03**	775 (722; 835)	785 (530; 868)	0.39
sIL-2R [IU/ml] **	n.a	1404(1097;2843)	n.a	1271(954;2133)	2129(1161;4359)	0.10
Ferritin [ng/ml] **	n.a	1815(863; 3674)	n.a	919 (810; 1752)	3572(1819;5343)	**0.03**
Noradrenalin dose [µg/h]	600 (0; 2000)	0 (0; 400)	0.05	600 (200; 1100)	1150 (0; 2000)	0.77
Leucocytes [10^9^/L]	10.0 (6.1; 12.3)	9.4 (6.8; 12.3)	0.90	8.4 (5.4; 11.3)	10.6 (5.9; 14.0)	0.42
Neutrophils (%)	84 (78; 90)	76 (72; 88)	**0.01**	85 (82; 90)	84 (77; 92)	0.36
Lymphocytes (%)	9 (4; 15)	11 (6; 14)	0.98	8 (4.5; 12)	13 (4; 17)	0.37
NLR	10 (10; 21)	6 (5; 13)	0.027	11 (7; 20)	7 (5; 26)	0.89
Monocytes (%)	3 (1; 5)	6 (2; 10)	**0.03**	3 (1; 5.5)	3 (1; 6)	0.54
Body temperature [°C]	38.2 (37.6; 39)	37.8 (37.3; 38.2)	0.20	38.5 (37.7; 38.7)	38.1 (37.4; 39.5)	0.70

Column 2–4: We report relevant clinical immunologic parameters at time of ICU-admission (ADM) and after *1 week (5–8), while patients that required intubation were still on mechanical ventilation. Paired t-tests, or paired Wilcoxon-rank-test, were used to test for intra-individual differences, as applicable (full cohort). Columns 5 and 6: Group differences (favourable versus unfavourable clinical courses) were assessed using ANOVA or Kruskal–Wallis-test. **Serum ferritin and sIL-2R receptor were assayed after a median of 2 days (missing data n = 1) post admission (ADM) and 4 days later – we report peak values. We report median and interquartile range (IQR) or frequencies as counts and percent of total. ANOVA was used for group comparisons: favourable versus unfavourable clinical course. Abbreviations: Interleukin (IL), C-reactive-protein (CRP), neutrophil/lymphocyte-ratio (NLR), soluble Interleukin 2 receptor (sIL-2R).

Whereas fever was volatile within the first week (temperature day 1: 38.2 [IQR; 37.6;39], day 8: 37.8 [IQR 37.3; 38.2]°C, *p* = 0.20), all patients maintained elevated D-Dimers (day 1: 2961 [IQR: 1498;6548]; day 8: 6492 [IQR: 3128;9618] ng/ml, *p* = 0.37), elevated CRP (day 1: 20.4 [IQR: 14.4;26.3], day 8: 14.3 [IQR: 8.1;27.3] mg/dl, *p* = 0.14) and IL-6 levels (day 1: 139 [IQR: 85.8;872], day 8: 68 [IQR: 38.5;615.5] pg/ml, *p* = 0.34). Of eight cases who developed significantly elevated PCT ≥ 1 µg/L within the first week, 6 (75%) had positive blood culture or tracheal specimen testing for bacterial superinfection.

On the first day post intubation, most patients required rather high noradrenaline doses (day1: 600 [IQR: 0; 2000] µg/h). Yet, after a week post ICU-admission, they rarely needed circulatory support (day 8: 0 [IQR: 0;400], *p* = 0.05). Cumulatively, these data indicate that the circulatory and pyrogenic impact of the cytokine release associated with COVID-19 was transient and often self-limiting (Table 2).

**Figure 2:**
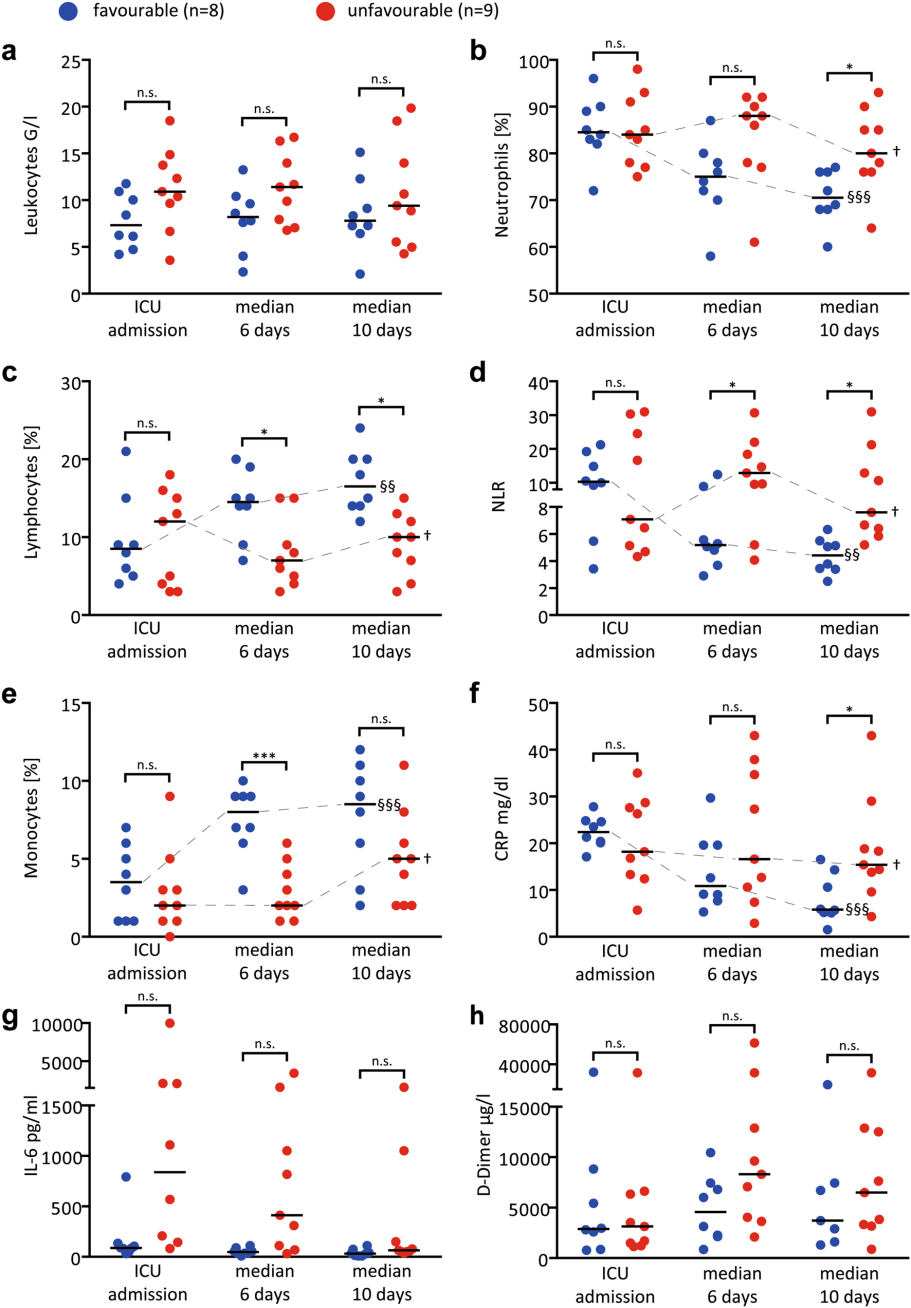
Immunologic parameters, neutrophilia and IL-6 in relation to clinical course at time of ICU admission, at day 4–7 (median 6 days)—when patients were still intubated—and prior to extubation if applicable (median 10 days). The median of 10 days differs from the median intubation time of 8.5 days (see main manuscript) since not all patients were intubated within 24 h post admission. Data from the unfavourable group (no extubation possible) were matched accordingly to achieve equivalent median days post ICU admission. Abbreviations: C-reactive-protein (CRP), Interleukin-(IL)-6, neutrophile/lymphocyte-ratio (NLR). This analysis includes 17 patients who had undergone intubation (1 patient each from the favourable and the unfavourable group were excluded, see methods section). Each datapoint represents one patient. Statistical significance for independent t-test between groups: n.s. = not significant, * = *p* < 0.05, ***p < 0.001. Statistical significance for repeated measure ANOVA † = not significant, § = *p* < 0.05, §§ = *p* < 0.01, §§§ = *p* < 0.001.

On the cellular level with varying absolute numbers of leucocytes, relative neutrophilia (84 [IQR: 78;90] %), lymphopenia (9 [IQR: 4;15] %), and reduced percentage of monocytes (3 [IQR: 1;5] %) were observed at ICU-admission (Table 2, Fig. 2 a-c, e). Consequently, neutrophil/lymphocyte ratio (NLR) was increased (10 [IQR: 5;21]; Fig. 2d). Immunophenotypic data revealed rather low numbers of circulating CD8 + T-cell subsets with a trend towards lower values in the unfavourable group (supplementary Table 1).

Strikingly, relative neutrophilia, lymphopenia and NLR paralleled the clinical course of our patients (Fig. 2b-d, Table 2). Of note, these intergroup differences could not be replicated in absolute cell numbers of the respective cell populations, likely due to the pronounced interindividual variation in leukocytes (supplementary Fig. 1). After the first week of intubation, patients in the favourable group separated and showed significantly decreased relative neutrophilia, lymphopenia and NLR the day prior to successful extubation, whereas NLR remained increased in the unfavourable group (favourable: 10.3 [IQR: 3.5;21.3] $\to$ 5.2 [IQR: 2.9;12.5] $\to$ 4.4 [IQR: 2.5;6.3] versus unfavourable: 7.8 [IQR: 4.3;31.9] $\to$ 12.9 [IQR: 4.1;12.9] $\to$ 7.6 [IQR: 5.2;31]; *p* = 0.03; Fig. 2b-d). The decline of CRP over this period was less pronounced and only separated the groups prior to extubation (Fig. 2f). Interestingly, despite a declining trend over the first week, serum IL-6-levels did not separate favourable and unfavourable clinical courses (median at tube removal: 32 [IQR: 13;49], versus 65 [IQR: 52;150] pg/ml, *p* = 0.15; Fig. 2g). D-Dimers tended to increase in both groups (repeated-measure-ANOVA: *p* = 0.13 or 0.2, Fig. 2h). Thus, an acute neutrophilic response rather than serum mediator kinetics paralleled the clinical course in our patients. The lack of organomegaly and lymphopenia suggest that hyperinflammation is linked to innate immunity.

### Assessment of virus induced HLH

Due to fever, hyperinflammation and high mortality, we asked whether patients with severe COVID-19-disease and organ dysfunction suffer from virus induced secondary HLH.

The median HScore of our patients was 122 (IQR: 63;145) or 157 (IQR: 98;180) if positive bone marrow histology criterion was assumed. Therefore, 6 (32%) patients would have reached the proposed cut-off of 169 for “suspicion of hemophagocytosis”. Herein a trend for higher HScores in the unfavourable group (139 [IQR: 95;156] versus 90 [IQR: 63;123.5], *p* = 0.1) was apparent, with 5/10 (50%) versus 1/9 (11%) patients reaching the cut-off of 169 in the respective groups. Substantial domains of the HScore, i.e. fever, spleno-, hepato-megaly, bi-cytopenia, were assessed positive in 5 (26%), 4 (21%), 5 (26%), 7 (37%) patients, respectively. None of the patients developed hypofibrinogenemia. Elevations in AST were detected in all patients (Table 3). 8 (42%) developed serum ferritin > 2000, and two exceeded 6000 ng/ml. Similar observations with an overall slightly lower median HScore of 155 [IQR: 98; 166] were made when calculating the HScore after one week post admission. Still 3 patients (30%) of the unfavourable group achieved the predefined cut-off (supplementary Table 2).

**Table 3:** Expert ratings, median HScores and modified HLH-2004 criteria fulfilled.

Parameter	Overall	Favourable (n = 9)	Unfavourable (n = 10)	*p*-value
	Median (IQR) / frequency			
HScore (BM assumed neg.)	122 (63; 145)	90 (63; 123.5)	139 (95; 156)	0.095
n > 169	1 (5.3%)	0 (0%)	1 (10%)	0.720
HScore (BM assumed pos.)	157 (98; 180)	125 (98; 159)	174 (130; 191)	0.095
n > 169	3 (32%)	1 (11%)	5 (50%)	0.156
Likelihood (%)	< 10%- 98%	< 10%—54%	< 10%—98%	n.a
Prior IS	2 (11%)	2 (22%)	0 (0%)	0.447
Fever (> 38.4 °C)	5 (26%)	2 (22%)	3 (30%)	0.780
Splenomegaly	4 (21%)	2 (22%)	2 (20%)	0.968
Hepatomegaly	5 (26%)	2 (22%)	3 (30%)	0.780
Bi-Cytopenia	7 (37%)	5 (56%)	2 (20%)	0.21
Tri-Cytopenia	3 (16%)	0 (0%)	3 (30%)	0.28
TAG > 132.7[mg/dl]	15 (79%)	7 (78%)	8(80%)	1
TAG > 354[mg/dl]	4 (21%)	2 (22%)	2 (20%)	0.968
Fibrinogen* < 250[mg/dl]	0 (0%)	0 (0%)	0 (0%)	1
Ferritin > 2000 [ng/ml]	8 (42%)	1 (11%)	7 (70%)	**0.028**
Ferritin > 6000 [ng/ml]	2 (11%)	0 (0%)	2 (20%)	0.497
AST > 30 U/l *	19 (100%)	9 (100%)	10 (100%)	1
Modified HLH 2004 cirteria	2 (0; 2)	1 (0; 2)	2 (1; 3)	0.113
n ≥ 4 criteria fullfilled:	0 (0%)	0 (0%)	0 (0%)	1
Sustained fever	3 (15.8%)	1 (11%)	2 (20%)	0.780
Sustained cytopenia	6 (32%)	1 (11%)	5 (50%)	0.156
Ferritin > 10.000[ng/ml]	1 (5.3%)	0 (0%)	1 (10%)	0.720
TAG-criterion	13 (68%)	5 (56%)	8 (80%)	0.400
sIL-2R > 2400 < 10.000	5 (26%)	1 (11%)	4 (40%)	0.315
Chart review (yes = 1)				
Expert-1: – HLH (yes)?	0 (0%)	0 (0%)	0 (0%)	1
Expert 2 – HLH (yes)?	0 (0%)	0 (0%)	0 (0%)	1
CRS-like?	11 (58%)	6 (67%)	5 (50%)	0.475
Undefined:	8 (42%)	3 (33%)	5 (50%)	0.475

The table reports median HScores for assumed negative and positive results for the bone marrow criterion. Groups: ”overall”, “favourable” versus “unfavourable” clinical group, statistical comparison was done comparing the latter two groups. Further frequencies of the HScore subdomains are reported. We do the same for the modified 2004 HLH criteria its subdomains. None of our experts detected evidence for HLH in any of the patients’ charts reviewed. CRS like inflammation was rated, when there was evidence for inflammatory disease in the absence of bacterial coinfection. These cases were classified as undefined. Abbreviations: bone marrow (BM) biopsy; Triglycerides (TAG), Aspartate-amino-transferase (AST), hemophagocytic lymphohistiocytosis (HLH), soluble Interleukin-2 receptor (sIL-2R). *The results were identical, when adjusted laboratory cut offs for hypofibrinogenemia and abnormal AST were used.

Nevertheless, the HScore is not intended for and has never been validated for use in an ICU-setting ^18^. Likewise, higher cut-off values for ferritin have been suggested ^19,20^.

We therefore applied “modified”—“2004 HLH-diagnostic-criteria”, which are considered positive in the presence of 4/6 of the following criteria: persistent fever, splenomegaly, persistent or progressive cytopenia, ferritin > 10.000 ng/ml, AST > 50 IU/l. As can be retrieved from Table 3 only one patient from the unfavourable group reached the modified cut-off for serum ferritin. The sustained fever- and cytopenia-criteria were reached by 3 (16%) and 6 (32%) patients, respectively. 13 (68%) patients showed hypertriglyceridemia (Table 3). Fever and cytopenia were judged in a strictly longitudinal manner. The remaining domains were identical to the HScore-domains (Table 3), this resulted in 0/19 patients fulfilling four of the “modified”—“2004-HLH-diagnostic- criteria”.

In line with this, sHLH usually implies massive activation of T-cellular immunity, which can be assayed via elevated sIL-2R ^21^. Although 5 (26%) of our patients showed values above 2000 U/ml, none exceeded the limit of 10,000 IU/ml, which has high specificity for sHLH.

Most importantly, sHLH is a clinical syndrome, which is ultimately defined by the assessment of the experienced clinician. We therefore had all case-charts (supplementary file 1) reviewed by experts. Both reached agreement, that the diagnostic criteria for classical sHLH were not fulfilled in any of the presented cases. (Table 3). However, CRS-like hyperinflammation associated with COVID-19 was assessed positive in 11 (58%) cases. 8 (42%) patients were defined “uncertain” regarding the latter due to concomitant superinfection (Table 3).

In summary, none of the patients showed evidence for secondary HLH within a median of 11 (8;16) days of observation after ICU-admission, despite the presence of ARDS, systemic hyperinflammation and eventually organ failure. Thus, virus-associated CRS but not sHLH is frequently associated with severe COVID-19.

## Discussion

Based on SARS-CoV-1 and preliminary results of recent anti-cytokine-targeted interventional studies, an exuberant immune response has already been extrapolated as a major cause of lung injury, organ failure and mortality in COVID-19 ^22^. In fact, hyperinflammation and hyperferritinemia are hallmarks of severe over moderate COVID-19 ^23,24^. Moreover, hypercoagulability causing thrombosis, a classical feature of secondary HLH, has been observed amongst around 30% of severe COVID-19 patients ^12^. And more recent evidence suggests, that this hyperinflammation can replicate different facets of both sHLH and viral CRS in different patient groups and different timepoints of disease courses ^12,23^. These observations gave rise to the idea, that “virus associated” CRS or even sHLH might drive severe COVID-19 ^7,25^.

Here we report on the clinical course and immunologic findings of 19 consecutive severe COVID-19-patients. We demonstrate that up to a median of 11 (8; 16) days post ICU-admission none of our patients showed classical evidence of sHLH, which practically excludes sHLH as an initial driver of severe COVID-19 in the majority of cases. We relied on various layers of evidence, including the screening for HLH using the HScore as suggested by Mehta P et al. only recent ^7^. Apart from high HScores in individual cases, all patients lacked the classical clinical triad, i.e. persistent fever, organomegaly and progressive cytopenia ^9,26^. In support of our data Wood et al. recently reported, that out of 40 severe COVID-19 cases, only three reached the H-score specific sensitivity cut-off of > 169. Of note, these patients lacked classical clinical HLH criteria, i.e. organomegaly ^27^. As also acknowledged by Wood et al., the HScore must be interpreted with caution in severe COVID-19-patients, since ferritin, fever, cytopenia and even the bone marrow criterion (“hemophagocytosis”) lose specificity in an ICU-setting where blood transfusions, drug-associated cytopenia, organ failure and sepsis are frequent events ^19,27–30^. Further, from a hitherto hypothetical point of view, severe COVID-19 can be seen per se as a hyperferritinemic syndrome. This makes the ferritin criterion of the H-score questionable, at least if it is not adjusted ^23^.

Aside from hyperinflammation and hypercoagulability (elevated D-Dimer), our patients lacked the classical immune-phenotypical features usually associated with sHLH/MAS including hypofibrinogenemia, expansion of CD8 + T cells and neutropenia ^31,32^. Instead, we report rather low counts of CD8 + T cells, lymphopenia and relative neutrophilia. These observations agree with various reports from Wuhan ^8,24^.

However, “absence of sHLH” is not contradictory to the hypothesis that virus-associated-CRS and hyperinflammation rather than viral replication determines the course of COVID-19 as some observations can hardly be explained by virus replication ^24^. Cases from France indicate, that development of severe COVID-19-pneumonia can be accompanied by a decreasing viral load ^33^. We and others find that deterioration of oxygenation occurs with some delay, about 3 days after hospital admission accompanied by the peak inflammatory reaction ^6^. Thereby, elevated inflammatory mediators, a hypercoagulable state (D-Dimer) and elevated NLR were characteristics of the disease at onset of ARDS. Cumulatively, these data confirm the presence of a strong virus-associated CRS in SARS-CoV-2-infection, simultaneously to the onset of ARDS ^6,24,34^. Our data adds to the current knowledge, that amongst severe COVID-19-patients, NLR and relative neutrophilia more than CRP paralleled the observed short-term clinical course. It is tempting to speculate that virus-associated CRS and the neutrophilic response are the main culprits in severe COVID-19-pneumonia, which occurs with a delay only when the “misguided anti-viral-immune response” hits ^25^. One might argue, that ARDS per se implies alveolar endothelial injury, complement-, neutrophil-activation, and NETosis and thus entails a systemic hyperinflammatory response ^35–37^. Still, others have reported, that an increased NLR is apparent already before the onset of ARDS, and was an independent predictor for severe COVID-19 in 61 patients ^34,38^. In addition, Zheng M et al. reported functional exhaustion of cytotoxic T cell subsets upon SARS-CoV-2 infection ^34^. This may not only hinder clearance of the virus but also override the cellular control mechanisms that normally inhibit the development of CRS ^25,34^.

Considering these data, combined anti-viral and anti-inflammatory treatment strategies are seemingly plausible options to improve outcomes. Although we could not find an association of serum IL-6 with the clinical course of our patients and although experimental data indicate potentially harmful effects of IL-6R blockade in experimental lung injury there have been occasional reports of therapeutic success with tocilizumab in COVID-19 ^25,39^. In addition, both IL-6 and IL-1 were associated with outcomes in ARDS decades ago ^40^. Still, we believe that a sense of proportion is required here.

In the absence of sHLH, cytoreductive drugs, i.e. etoposide, are not indicated. Further, we advocate caution when applying combined cytokine-directed treatment protocols in this vulnerable patient population. Our patients are elderly, carry comorbidities and are not immune to bacterial superinfections. The latter was also observed in a larger cohort ^6^. After all, 9/19 patients improved and overcame viral-CRS by standard supportive care. Thus, treatment within clinical trials should be prioritized when using experimental immunomodulation. Moreover, short acting substances, e.g. IL-1R-blockade with anakinra for which positive data exist from other neutrophilic hyperinflammatory conditions such as Still's disease with ARDS and septic shock deserves special consideration, as it allows the therapy to be quickly de-escalated ^41,42^. Also, Ruxolutinib a tyrosine kinase inhibitor interfering with Jak-STAT dependent cytokine signalling seems appealing (NCT 04338958). If neutrophils prove to be causally involved, colchicine could be another short-acting and cost-effective option for emerging countries, (NCT04322682)^43^.

Our short-term observational study has limitations. First, the overall sample size is relatively small with only a short follow up. In addition, 2 patients were transferred to our ward more than 48 h after external ICU admission, thus datasets for sIL-2R and ferritin were not complete. We can indeed exclude sHLH as a common underlying pathology in severe COVID-19 with ARDS. However, we cannot eliminate the possibility that some patients will develop secondary HLH later on, e.g. as a result of bacterial sepsis or prolonged viral-CRS ^44^. Moreover, the judgment of the (un)favourable disease-courses based on clinical criteria reflects a rather weak clinical endpoint and holds risk for bias. Nevertheless, all patients classified as favourable were permanently discharged from ICU at time of manuscript preparation. Further, in clinical reality, therapeutic decisions will likely also depend on similar clinical assessments. Yet, if immune-modulatory drugs meet our expectations, our observations raise the questions of (a) which (sub)groups should be treated and (b) at what timepoint ^12^, to not endanger patients with self-limiting courses by overtreatment.

## Methods

### Study design and patient characteristics and clinical course

This observational cohort study is a pilot study analysis of the multi-centre register of “COVID-19-register to document cases of secondary hemophagocytic lymphohistiocytosis” NCT04347460. All patients, their families or legal guardians gave written and informed consent. The study was approved by the local ethics committee of the Klinikum rechts der Isar of the Technical University of Munich (Ref. 161/20 S) and accordance with the declaration of Helsinki.

Patients’ characteristics were entered based on interviews with patients or families and the clinic’s medial record system. BMI, Horovitz Index (HI) and sequential-organ-failure-assessment-(SOFA)-Score were calculated on admission as body weight [kg]/height^2^ [m^2^], paO2/FiO2 or using the online-calculator https://mdcalc.com/sequential-organ-failure-assessment-sofa-score, respectively. Clinical data were independently reviewed by 2 researchers.

Severe COVID-19, shock and ARDS were defined according to the National Health Commission of China’s and the World Health Organization’s interims papers definition, respectively ^45,46^. As observation periods were limited, favourable spontaneous clinical course of severe COVID-19-patients were defined by either “no need for intubation” or “was extubated” at time of analysis versus those who were in need of mechanical ventilation, or in need of extracorporeal lung assist devices, prolonged weaning, or had died at time of analysis.

The datasets generated during and/or analysed during the current study are available from the corresponding author on reasonable request.

### Scores and assessment for HLH

The HScore was calculated at time of ICU-admission and after 1 week as described by Fardet et al. (see Table 4 and supplementary Table 1, respectively ^18^). Yet, in an ICU-setting only a cut-off value for ferritin ≥ 10.000 ng/ml demonstrated sufficient sensitivity in these patients ^19^. The bone marrow biopsy criterion was further shown to have poor specificity for HLH among seriously ill patients ^47^. Thus, the pediatric 2004-HLH criteria, were modified according to centre specific cut-offs, and a cut-off for ferritin of 10.000 ng/ml was used (“modified 2004-HLH criteria”; Table 4). Chart reviews to determine actual presence of sHLH were independently performed by L.G. M.P. and L.R.P., the latter two having at least ten years of experience in diagnosis and treatment of secondary HLH.

**Table 4:** HScore criteria as reported by Fardet et al. ^18^ and “modified 2004-HLH-diagnostic-criteria”.

HScore – Sum of points*	Modified 2004 diagnostic criteria *	Points
Immunosuppression: 18	n.d	
Fever: 0 (strictly < 38.4), 33 (38.4–39.4), or 49 (strictly > 39.4)	Fever ≥ 38.5 °C (if persistent -7d)	+ 1
Organomegaly: 0 (no), 23 (hepatomegaly or splenomegaly), or 38 (hepatomegaly and splenomegaly)	Splenomegaly	+ 1
Cytopenia (lowest within 7d):	Cytopenia	+ 1
0 (one lineage), 24 (two lineages), or 34 (three lineages):	(at least bi-lineage, persistent / progressive within 7d)	
Hgb ≤ 92 g/L	Hgb < 90 g/L	
Platelets ≤ 110 × 10^9^/L	Platelets < 100 × 10^9^/L	
Leucocytes ≤ 5 × 10^9^/L	Neutrophils < 1 × 10^9^/L*	
Ferritin: 0 (< 2000 ng/ml), 35 (2000–6000 ng/ml), or 50 (> 6000 ng/ml)	Ferritin ≥ 10,000 ng/ml *	+ 1
TAG [mg/dl]: 0 (< 132.7), 44 (132.7–354), or 64 (> 354)	TAG [mg/dl]* > 150	+ 1
Fibrinogen [mg/dl]: 0(> 250) or 30 (≤ 250)	Fibrinogen [mg/dl] * < 200	+ 1
AST ≥ 30 U/l: 19	n.d	
Hemophagocytosis in bone marrow – not assessed (35 points)	Hemophagocytosis in bone marrow – not assessed	n.a

*We used centre specific cut-offs for definition of hypo-fibrinogenaemia, hypertriglyceridemia, leucopenia and neutropenia with respect to the “modified HLH2004 criteria”. We used lower or upper laboratory specific reference ranges to define the adjusted cut offs. In addition the ferritin criterion was modified within the adjusted 2004 HLH-diagnostic guidelines to a cut off of ≥ 10.000 ng/ml, according to better specificity in ICU-patients ^19^. The HScore is calculated as a sum of points – see Fardet et al. ^18^. The sum-score can be transformed into a probability score of HLH – however, this has never been validated for assessment of HLH in an ICU-setting. Abbreviations: Aspartate-aminotransferase (AST), Haemoglobin (Hgb), triglycerides (TAG).

### Laboratory work and diagnostic procedures

PCR-testing for SARS-CoV2 was performed from tracheal specimens. 18/19 patients had positive results. One patient with disease specific radiology report ^15,16^, repeatedly tested negative from tracheal specimens, but demonstrated seroconversion for SARS-CoV-2-IgM and IgG. Routine laboratory parameters, sIL-2-receptor (sIL-2R, missing data n = 1) and lymphocyte phenotyping (missing data n = 1) were assayed by clinical central laboratories. Presence of splenomegaly or hepatomegaly were determined from CT-scans or via sonography. Predefined cut-offs were 14 cm or 16 cm respectively ^48–50^.

#### Statistics

Was performed using IBM SPSS Version 23. We report median and interquartile range (IQR) or counts and percent of total (%) as applicable. Group comparisons (favourable versus unfavourable clinical group or overall cohort at ICU admission or after 1 week) were done using ANOVA, Mann–Whitney-U-, Kruskal–Wallis-, repeated measure paired ANOVA or paired-Wilcoxon-rank-test as applicable.

## Supplementary information

Supplementary Information 1.

Supplementary Information 2.

Supplementary Information 3.
