# Supplementary figures and images for: Title: Cytokine release syndrome is not usually caused by secondary hemophagocytic lymphohistiocytosis in a cohort of 19 critically ill COVID-19 patients
Source: Sci Rep. 2020 Oct 26;10:18277. doi: 10.1038/s41598-020-75260-w (PMC7589537; doi:10.1038/s41598-020-75260-w)

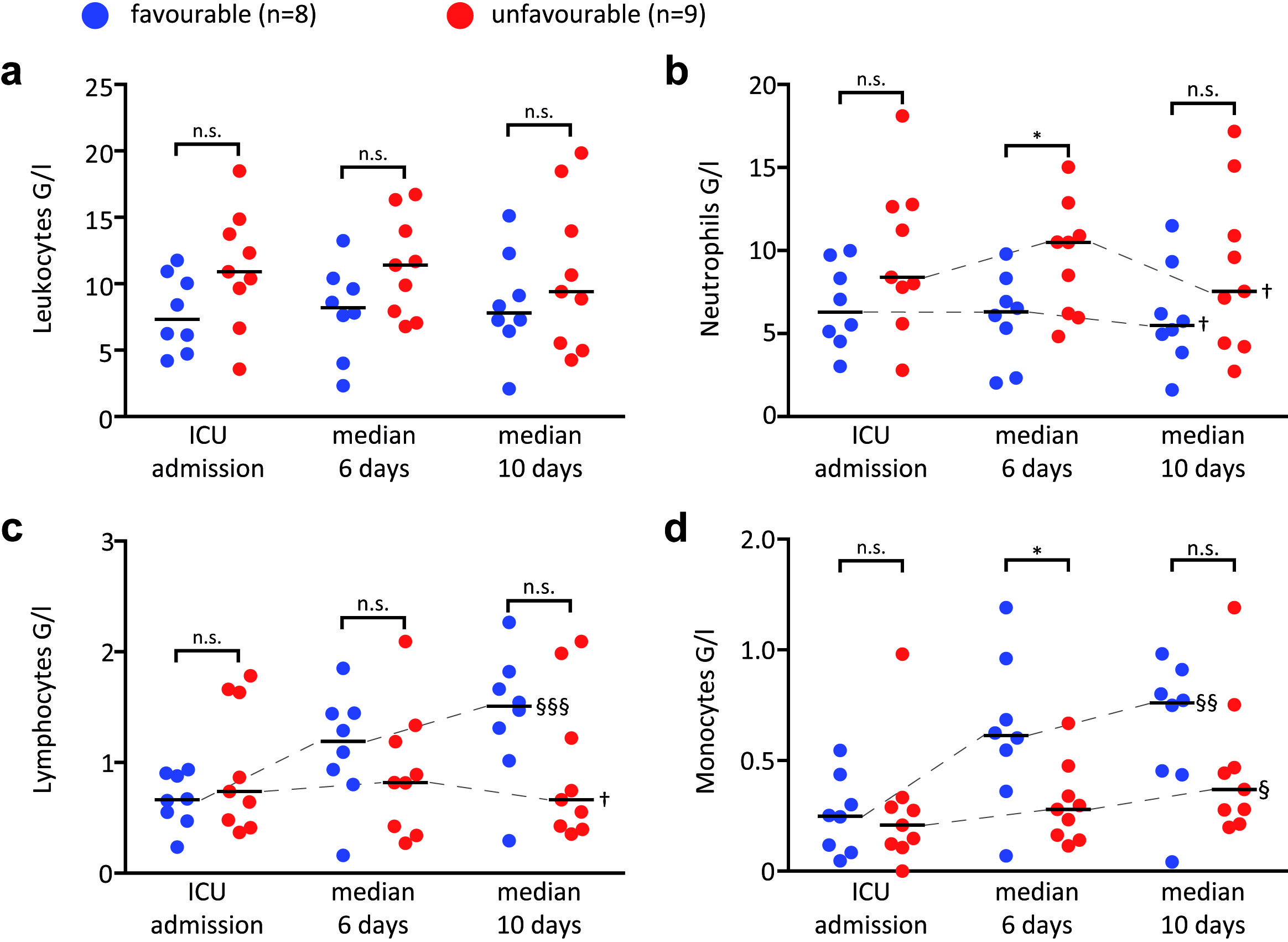

Supplement: Supplementary file 1 — Supplementary Information 1. [file 41598_2020_75260_MOESM1_ESM.jpg]
