# Supplementary material for: Title: Cytokine release syndrome is not usually caused by secondary hemophagocytic lymphohistiocytosis in a cohort of 19 critically ill COVID-19 patients
Source: Sci Rep. 2020 Oct 26;10:18277. doi: 10.1038/s41598-020-75260-w (PMC7589537; doi:10.1038/s41598-020-75260-w)
Supplement: Supplementary file 2 — Supplementary Information 2. [file 41598_2020_75260_MOESM2_ESM.docx]

**Supplementary information: Cytokine release syndrome is not usually caused by secondary hemophagocytic lymphohistiocytosis in a cohort of 19 critically ill COVID-19 patients**

Georg Lorenz^1^*^,#^, Philipp Moog^1^*^,#^, Quirin Bachmann^1^, Paul La Rosée^2^, Heike Schneider^3^, Michaela Schlegl^1^, Christoph Spinner^4^, Uwe Heemann^1^, Roland M. Schmid^4^, Hana Algül^4,5^, Tobias Lahmer^4^, Wolfgang Huber^4,^*, Christoph Schmaderer^1,6,^*

^1^ Technical University of Munich, School of Medicine, Klinikum rechts der Isar, Department of Nephrology, Ismaninger Str. 22, 81675 Munich, Germany

^2^ Clinic for Internal Medicine II, Schwarzwald-Baar Klinikum Villingen-Schwenningen, Klinikstr. 11, 78052 Villingen-Schwenningen, Germany

^3^ Technical University of Munich, School of Medicine, Klinikum rechts der Isar, Department for Clinical Chemistry, Ismaninger Str. 22, 81675 Munich, Germany

^4^ Technical University of Munich, School of Medicine, Klinikum rechts der Isar, II. Department for internal medicine, Ismaninger Str. 22, 81675 Munich, Germany

^5^ Comprehensive Cancer Center Munich at the Klinikum rechts der Isar, Technische Universität München

^6^ German Center for infectious research (DZIF), Technische Universität München

*** equal contribution**

**Supplementary table 1**: Cross sectional immune-phenotypic data after ICU-admission

| Parameter (reference values) | Overall (n=19) | Favourable  (n=9) | Unfavourable (n=10) | p-value |
| --- | --- | --- | --- | --- |
|  | Median (IQR) | | |  |
| Leukocytes (4.0-9.0 G/l) | 8.3 (7.3; 11.8) | 8.3 (7.3; 11.2) | 8.5 (6.5; 12.1) | 0.75 |
| B-cells CD19+ (90-580/µl) | 161 (63; 299) | 134 (43; 299) | 170 (40; 272) | 0.72 |
| T-cells CD3+ (850-2580/µl) | 886 (280; 1108) | 1105 (885; 1137) | 629 (258; 887) | 0.25 |
| CD4+ T-cells (490-1760/µl) | 629 (227; 710) | 648 (595; 752) | 379 (202; 679) | 0.54 |
| CD8+ T-cells (140-880/µl) | 189 (76; 254) | 233 (208; 459) | 140 (64; 169) | 0.08 |
| CD4/ CD8-Ratio (1.0-5.8) | 3.0 (1.5; 4.9) | 2.8 (1.2; 4.0) | 3.4 (2.1; 4.9) | 0.26 |
| NK cells CD16/56+CD3- (60-1020/µl) | 81 (46; 192) | 136 (80; 192) | 69 (29.5; 96) | 0.31 |
| Monocytes (4- 10%) | 7 (3; 10) | 9 (7; 10) | 4 (2; 9) | 0.98 |

Flow cytometric immune phenotyping was done after a median of 2 days post admission. We report median and interquartile range (IQR). ANOVA was used for group comparisons: favourable versus unfavourable clinical course. Abbreviations: Natural killer (NK)

**Supplementary table 2**: HScore at 1 week post admission

| Parameter | Overall | Favourable (n=9) | Unfavourable  (n=10) | p-value |
| --- | --- | --- | --- | --- |
|  | median (IQR) / frequency | | |  |
| HScore (BM assumed neg.) | 119 (62; 131) | 63 (63; 110) | 126 (95; 138) | 0.071 |
| - n >169 | 0 (0%) | 0 (0%) | 0 (0%) | 1 |
| HScore (BM assumed pos.) | 155 (98; 166) | 98 (98; 145) | 161 (130; 173) | 0.071 |
| - n >169 | 3 (16%) | 0 (0%) | 3 (30%) | 0.278 |
| - likelihood (%) | <10% - 80% | <10% - 40% | <10% - 80% | n.a |
| - Prior IS* | 2 (11%) | 2 (22%) | 0 (0%) | 0.447 |
| - Fever (>38.4°C) | 3 (16%) | 1 (11%) | 2 (20%) | 0.780 |
| - Splenomegaly* | 4 (21%) | 2 (22%) | 2 (20%) | 0.968 |
| - Hepatomegaly* | 5 (26%) | 2 (22%) | 3 (30%) | 0.780 |
| - Bi-Cytopenia | 3 (16%) | 2 (22%) | 1 (10%) | 0.66 |
| - Tri-Cytopenia | 1 (5%) | 0 (0%) | 1 (10%) | 0.72 |
| - TAG >132.7, <= 354 [mg/dl] | 11 (58%) | 5 (56%) | 6 (60%) | 0.91 |
| - TAG > 354[mg/dl] | 4 (21%) | 2 (22%) | 2 (20%) | 0.97 |
| - Fibrinogen* <250[mg/dl] | 0 (0%) | 0 (0%) | 0 (0%) | 1 |
| - Ferritin* > 2000 <= 6000[ng/ml] | 6 (32%) | 1 (11%) | 5 (50%) | 0.16 |
| - Ferritin* >6000 [ng/ml] | 2 (11%) | 0 (0%) | 2 (20%) | 0.50 |
| - AST>30 U/l * | 19 (100%) | 9 (100%) | 10 (100%) | 1 |

The HScore was again calculated based on clinical and laboratory data after the first week post ICU admission. *Ferritin and fibrinogen levels were reassessed after 4-7d post ICU admission, organomegaly was assessed only once during the first week in attempt to reduce exposure of staff members. Similarly, the domain “prior immunosuppression” remains identical to Hscore at admission / during the first week.

**
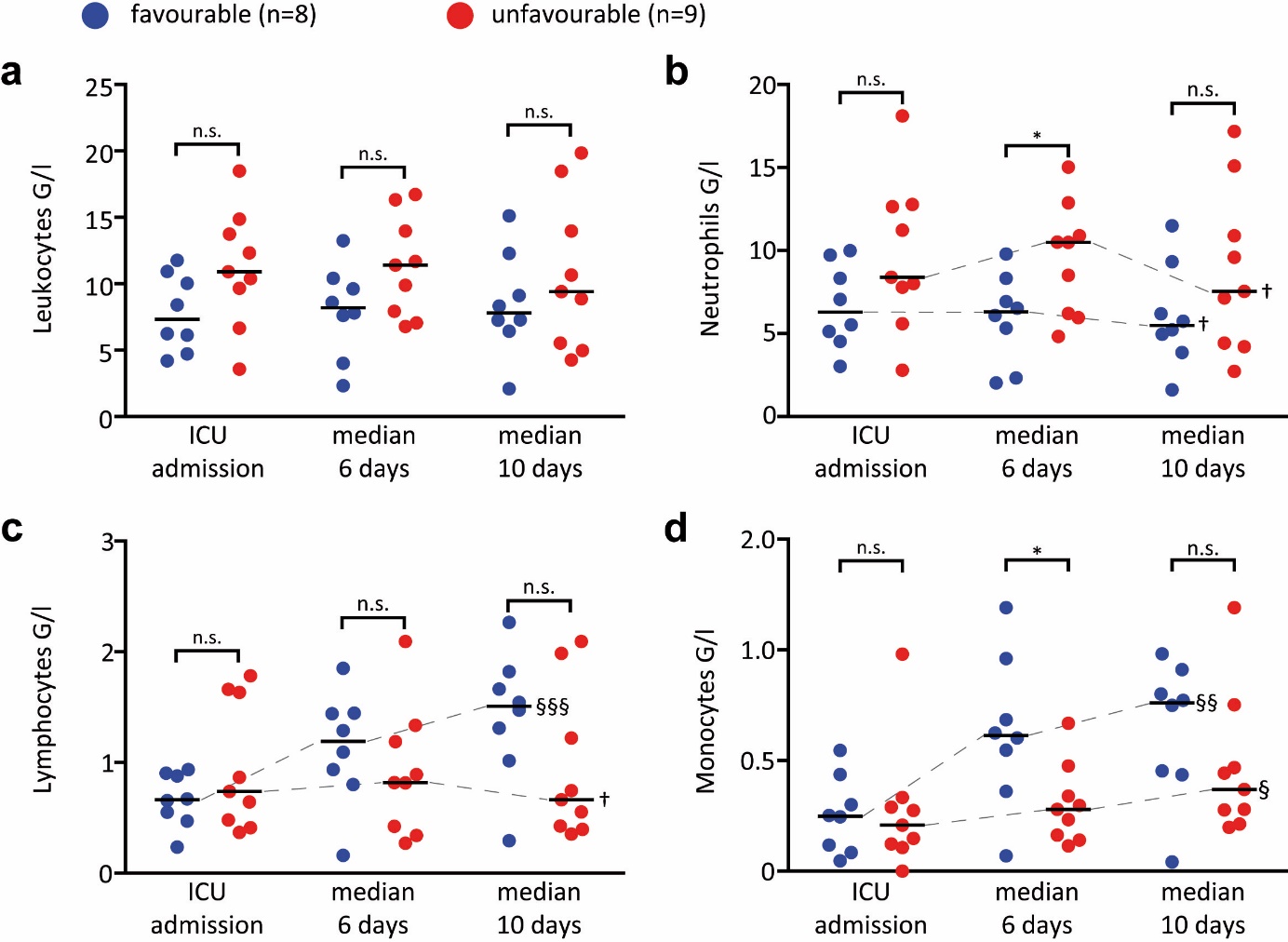
**

**Supplementary figure 1:** Absolute cell counts for (a) leucocytes, (b) neutrophils, (c) lymphocytes and (d) monocytes in analogy to figure 1 were stratified and compared by groups (“favourable” versus “unfavourable”); Differential blood cell counts were assessed (from left to right) at ICU admission, at day 4-7 (median 6 days), when patients were still intubated and prior to extubation if applicable (median 10 days). For the unfavourable group blood counts and inflammatory parameters were selected from a comparable timepoint (matched for a median of 10 days). This analysis includes 17/19 patients that were admitted to ICU (1 patient each from the favourable and the unfavourable group were excluded, see methods section). Each datapoint represents one patient. Statistical significance for independent t-test between groups: n.s. = not significant, * = p<0.05, ***p<0.001. Statistical significance for grouped anova † = not significant, § = p<0.05, §§ = p < 0.01, §§§ p < 0.001.
