# Supplementary material for: Title: Cytokine release syndrome is not usually caused by secondary hemophagocytic lymphohistiocytosis in a cohort of 19 critically ill COVID-19 patients
Source: Sci Rep. 2020 Oct 26;10:18277. doi: 10.1038/s41598-020-75260-w (PMC7589537; doi:10.1038/s41598-020-75260-w)
Supplement: Supplementary file 3 — Supplementary Information 3. [file 41598_2020_75260_MOESM3_ESM.pdf]

Supplementary information: Cytokine release syndrome is not usually caused by secondary hemophagocytic lymphohistiocytosis in a cohort of 19 critically ill COVID-19 patients

Georg Lorenz[1*,#], Philipp Moog[1*,#], Quirin Bachmann[1], Paul La Rosée[2], Heike Schneider[3], Michaela Schlegl[1], Christoph Spinner[4], Uwe Heemann[1], Roland M. Schmid[4], Hana Algül[4,5], Tobias Lahmer[4], Wolfgang Huber[4,*], Christoph Schmaderer[1,6,*]

[1] Technical University of Munich, School of Medicine, Klinikum rechts der Isar, Department of Nephrology, Ismaninger Str. 22, 81675 Munich, Germany

[2] Clinic for Internal Medicine II, Schwarzwald-Baar Klinikum Villingen-Schwenningen, Klinikstr. 11, 78052 Villingen-Schwenningen, Germany

[3] Technical University of Munich, School of Medicine, Klinikum rechts der Isar, Department for Clinical Chemistry, Ismaninger Str. 22, 81675 Munich, Germany

[4] Technical University of Munich, School of Medicine, Klinikum rechts der Isar, II. Department for internal medicine, Ismaninger Str. 22, 81675 Munich, Germany

[5] Comprehensive Cancer Center Munich at the Klinikum rechts der Isar, Technische Universität München

[6] German Center for infectious research (DZIF), Technische Universität München

*** equal contribution**

ID 1; m, 45 years

Relevant comorbidities:
- Asthma
- Arterial hypertension

Immunosuppression: no

Splenomegaly: yes

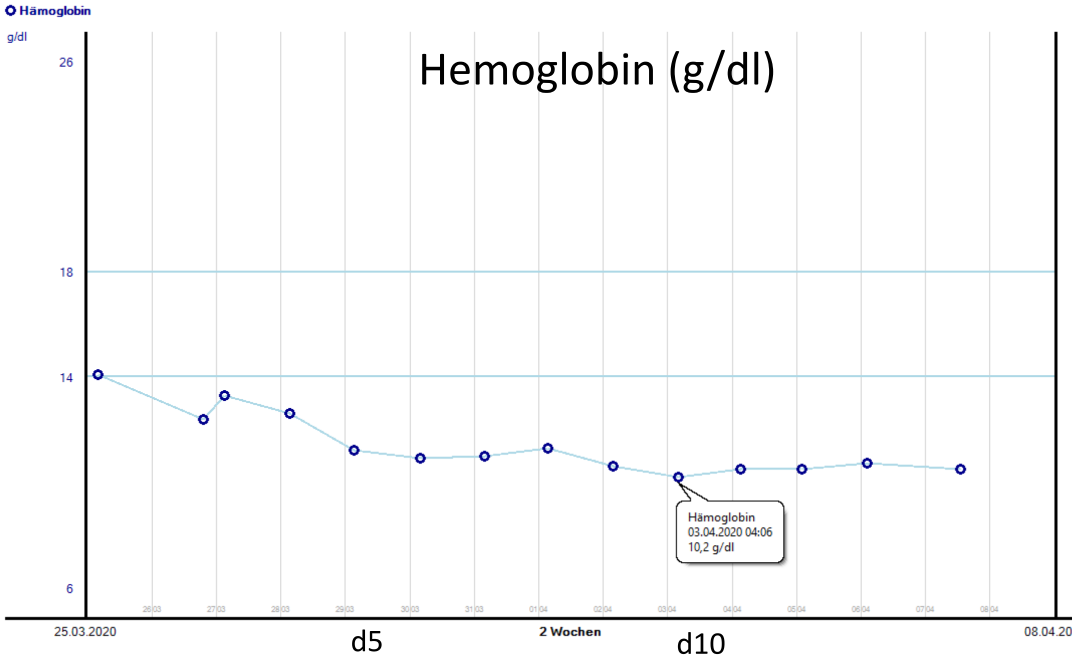

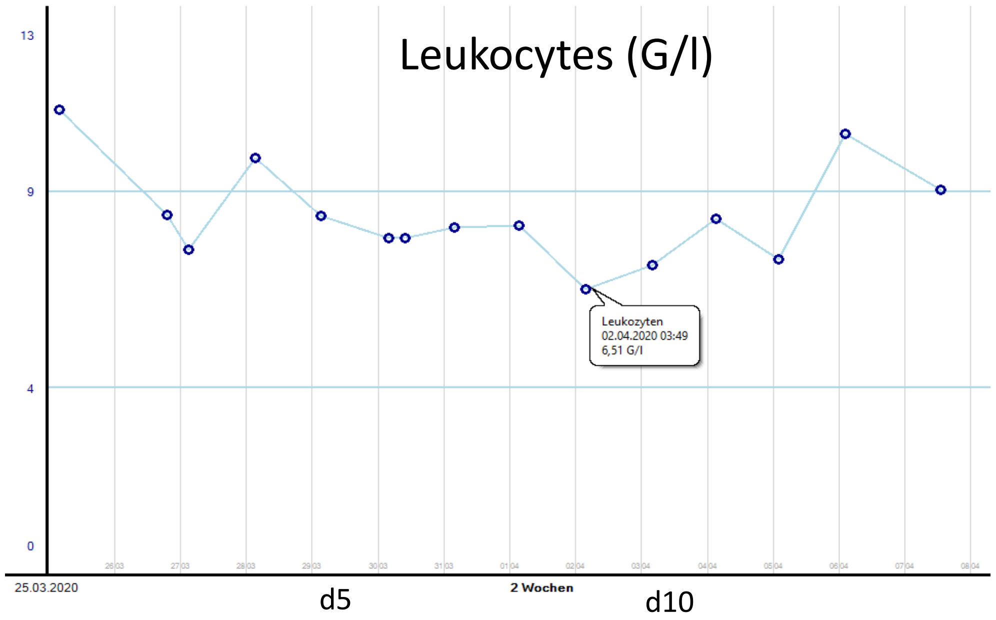

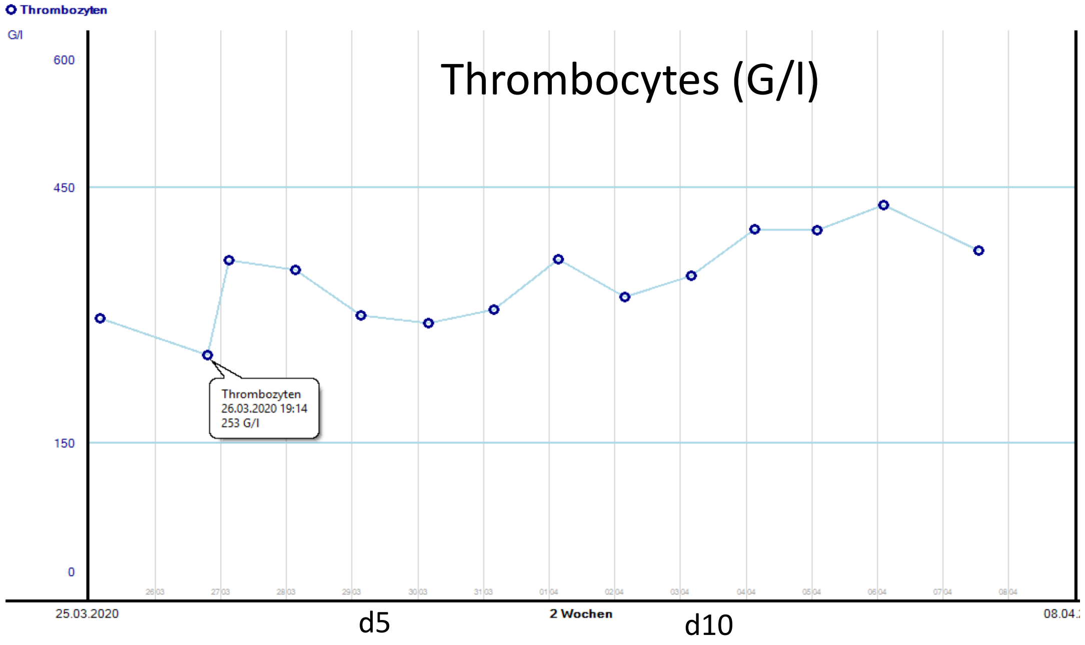

ID 1; m, 45 years

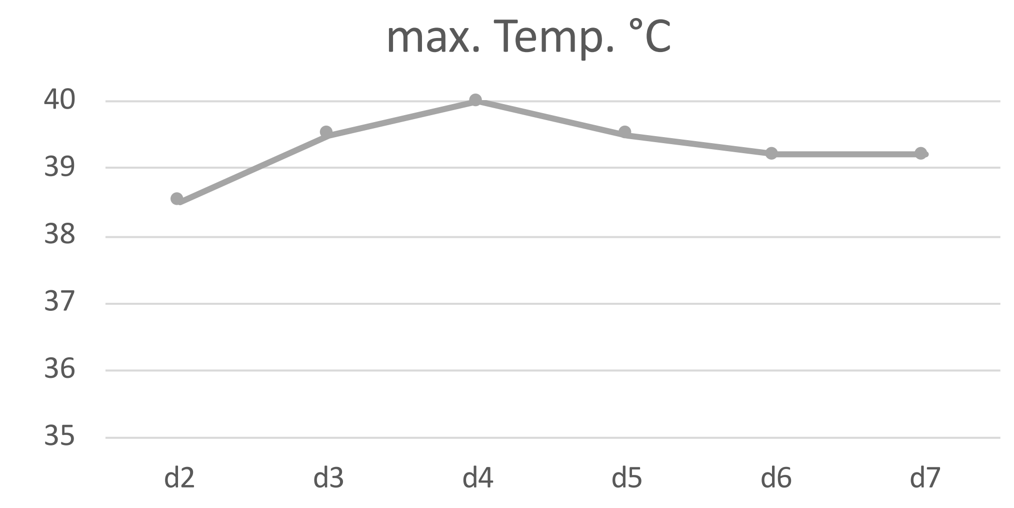

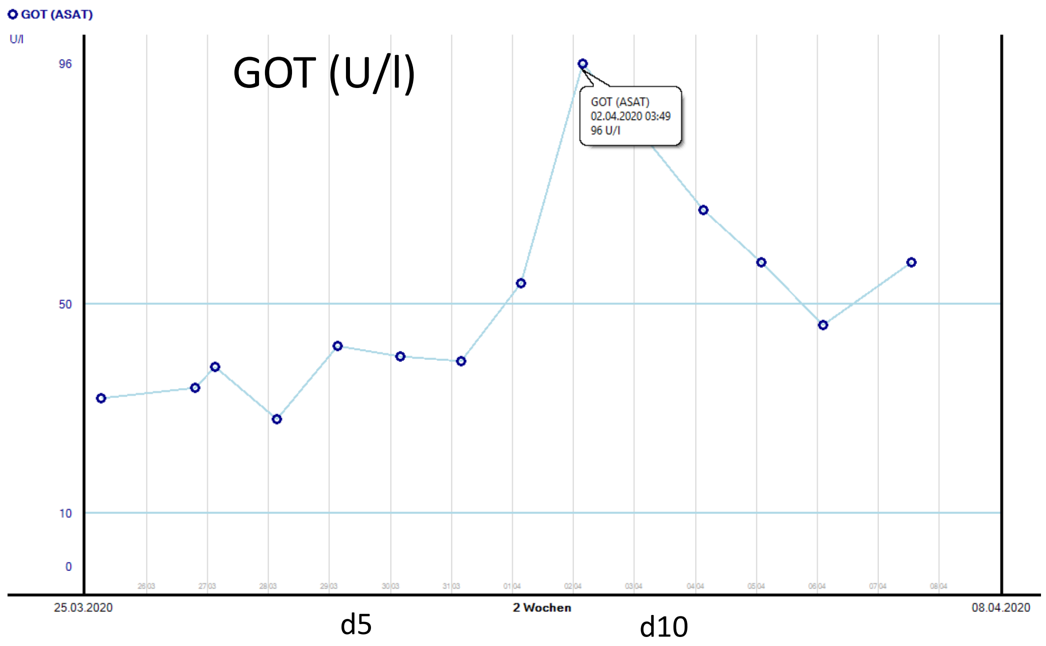

	d1	d2	d3	d4	d5	d6	d7	d8	d9	d10	d11
Ferritin (ng/ml)		618				842			782		
sIL2-R (U/ml)					2760						
Fibrinogen (mg/dl)		775				780					
Triglycerides (mg/dl)		92									
INR	1.0						1.0		1.0		
D-Dimer (µg/l FEU)	3128									6710	5347
Bilirubin (mg/dl)	0.6						0.5		0.4		
Procalcitonin (ng/ml)	0.3						0.1		0.1		

ID2; f, 58 years

Relevant comorbidities:
AML, first diagnosis Mar 2019
PBSCT Jul 2019

Immunosuppression: yes; Azacitidin

Splenomegaly: no

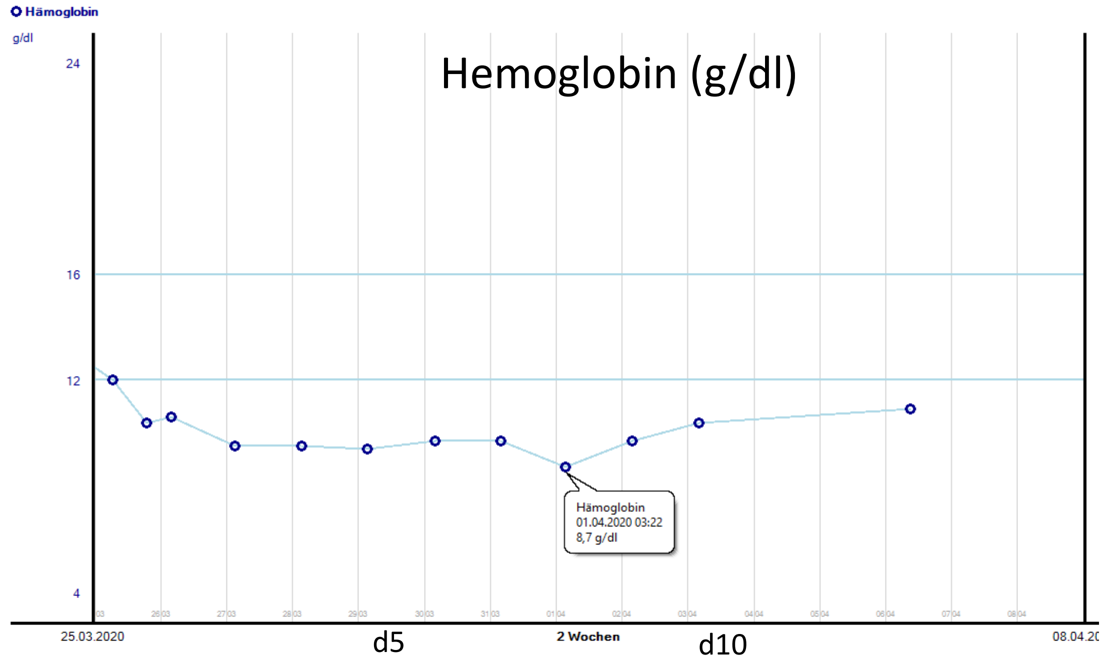

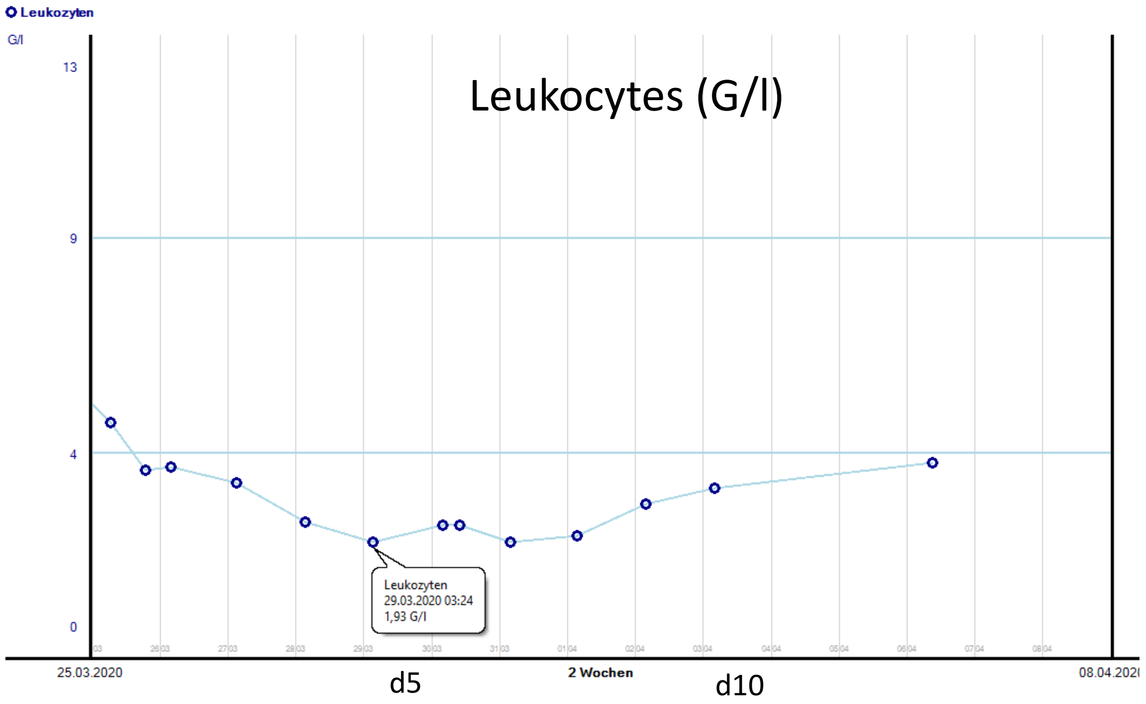

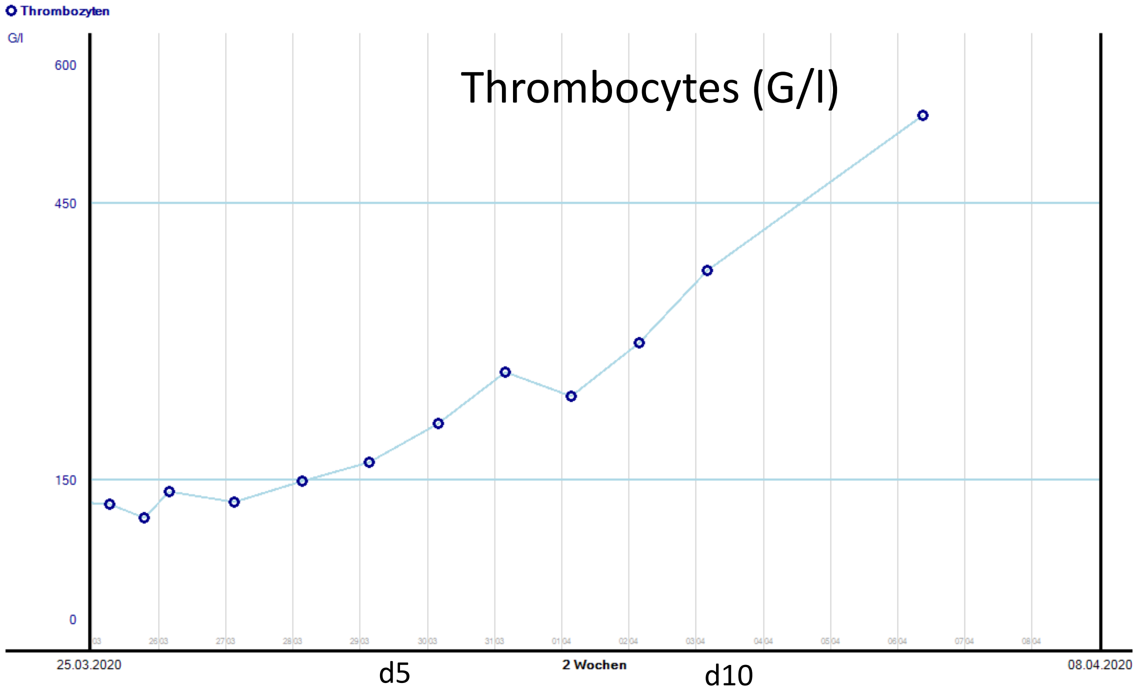

ID2; f, 58 years

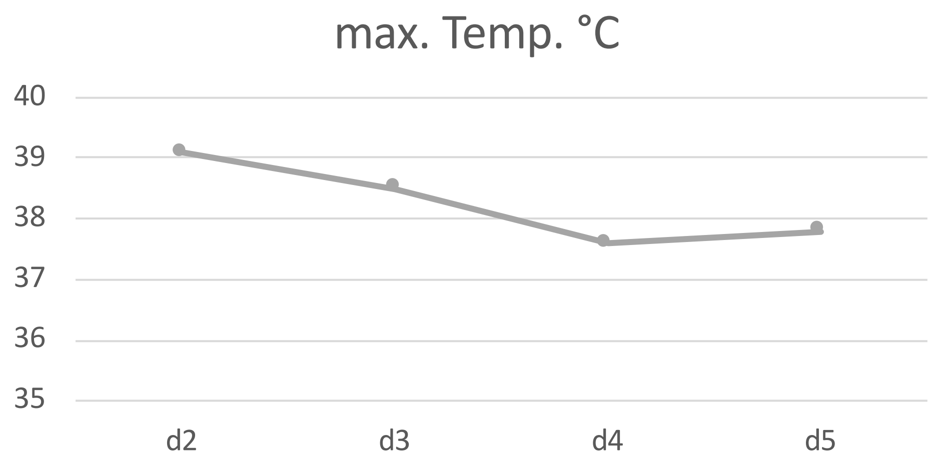

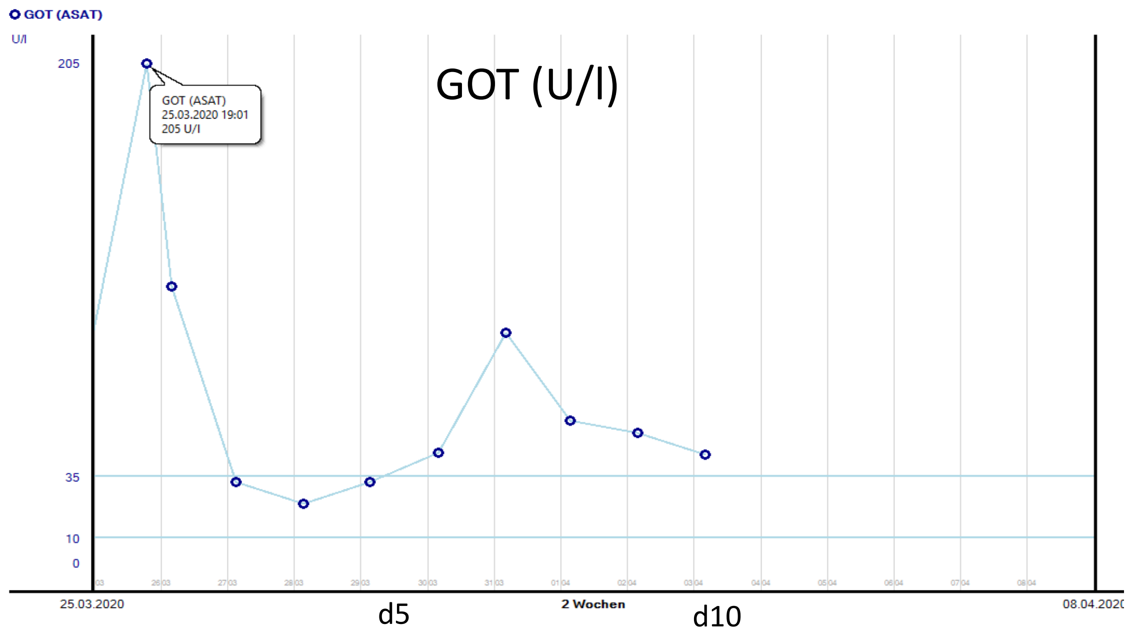

	d1	d2	d3	d4	d5	d6	d7	d8	d9	d10
Ferritin (ng/ml)				1570			1815		1138	
sIL2-R (U/ml)						1402				
Fibrinogen (mg/dl)			809				1013		714	
Triglycerides (mg/dl)			121							
INR	1.0					0.9			0.9	
D-Dimer (µg/l FEU)	2588						7446			6979
Bilirubin (mg/dl)	0.6					0.6			0.4	
Procalcitonin (ng/ml)	0.5						0.2			0.1

ID3; m, 76 years

Relevant comorbidities:
Arterial hypertension
COPD

Immunosuppression: no

Splenomegaly: no

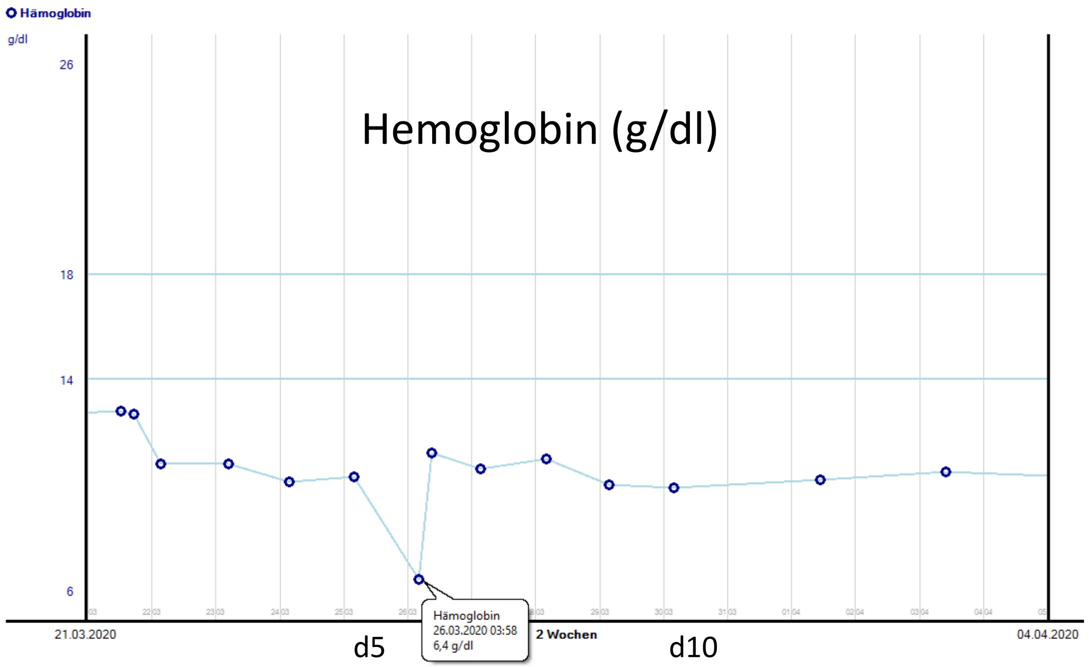

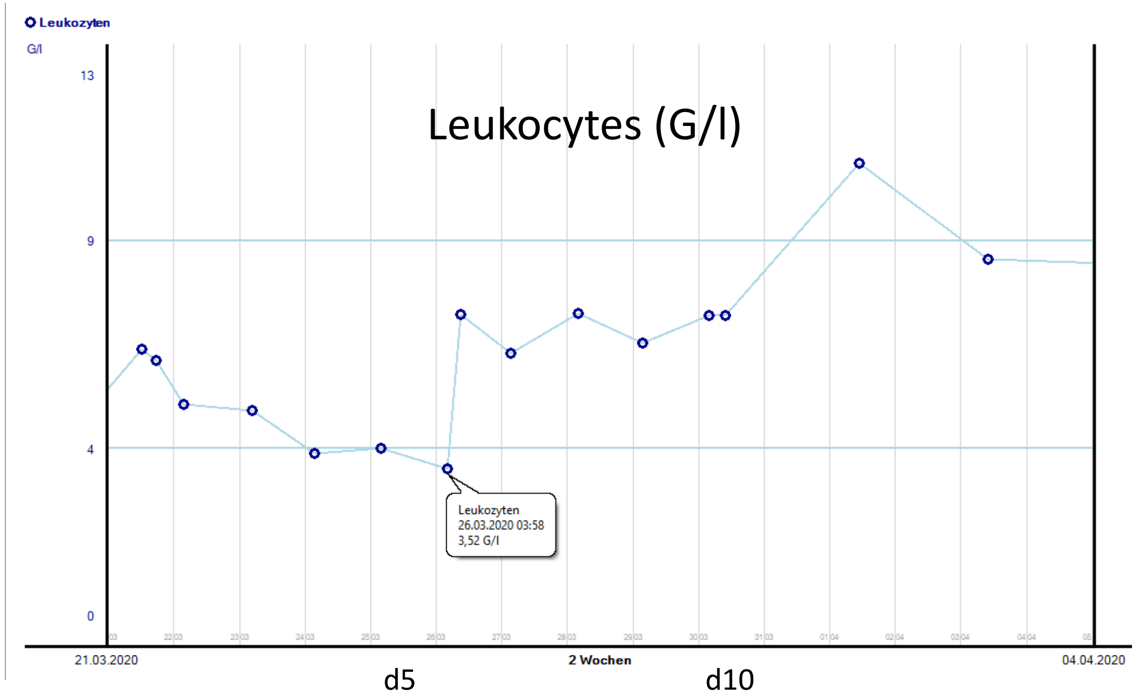

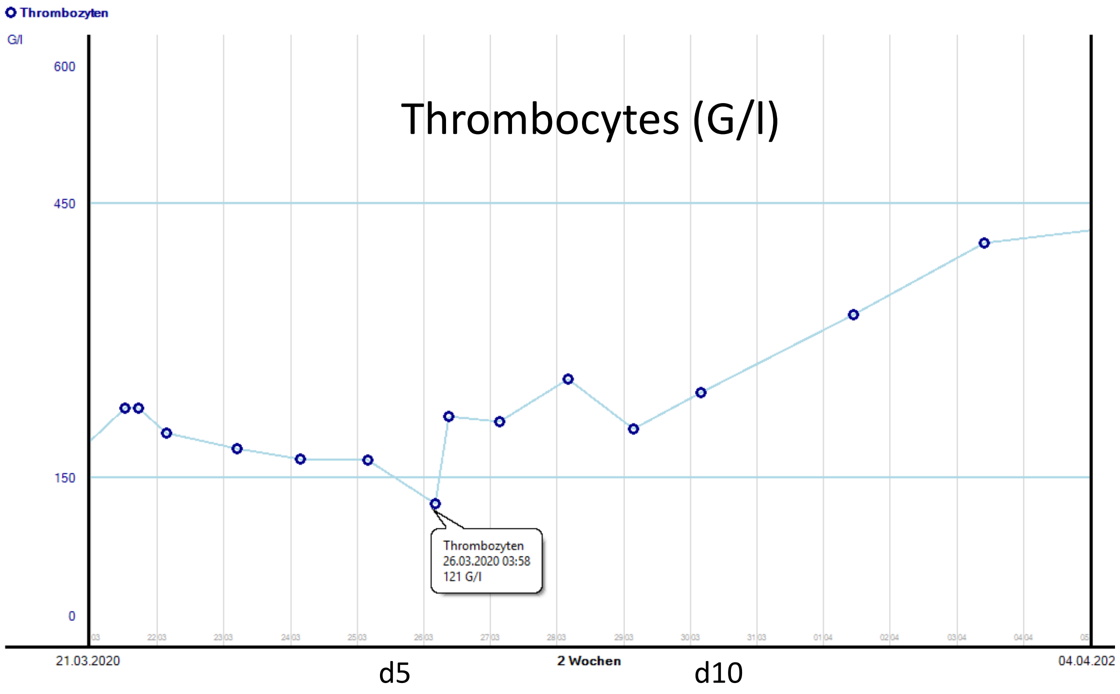

ID3; m, 76 years

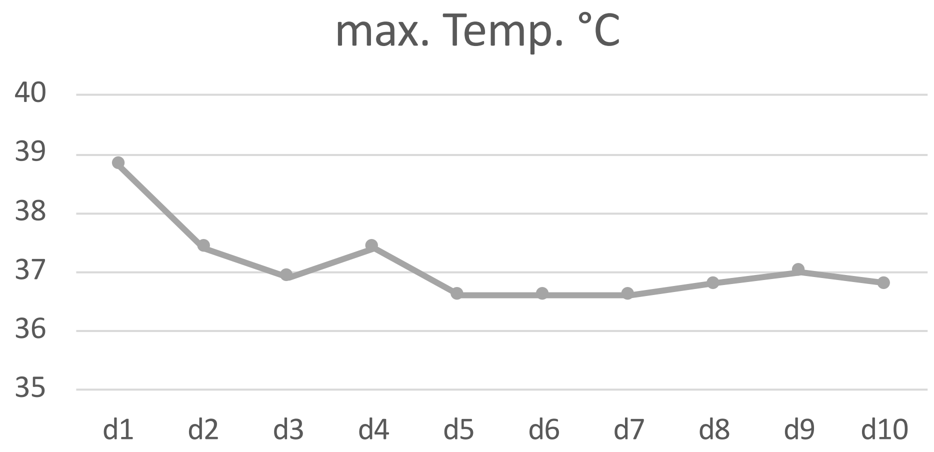

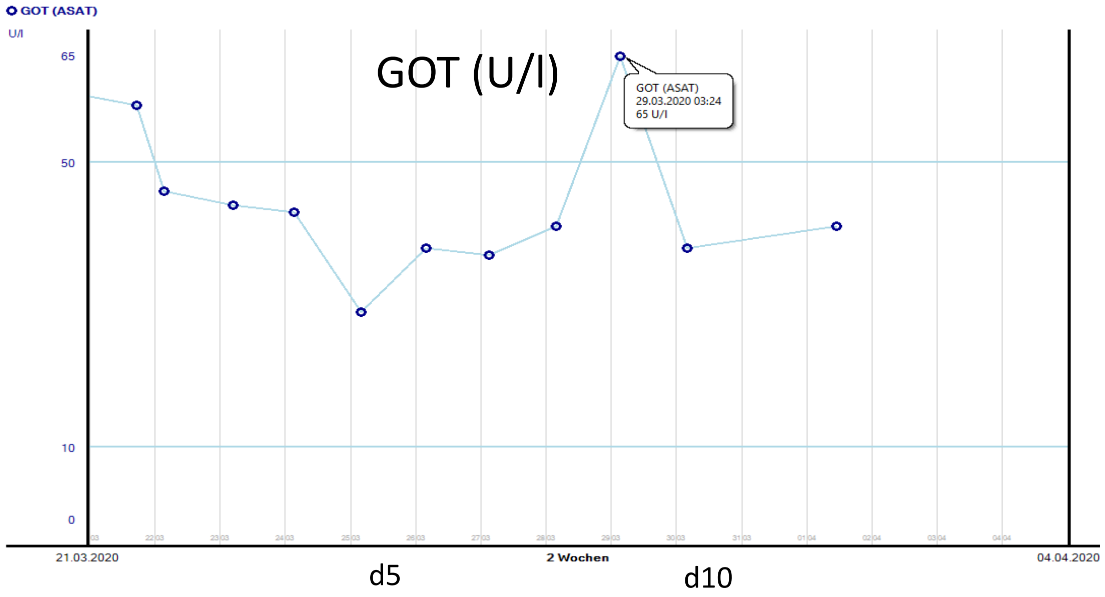

	d1	d2	d3	d4	d5	d6	d7	d8	d9	d10
Ferritin (ng/ml)								2045		
sIL2-R (U/ml)										1975
Fibrinogen (mg/dl)			605				877			
Triglycerides (mg/dl)			136				127			
INR	1.0			1.0			1.0			
D-Dimer (µg/l FEU)	2265						2961	3794		
Bilirubin (mg/dl)	0.5			0.3			0.3			
Procalcitonin (ng/ml)	0.4			0.3			0.3			

ID4; m, 84 years

Relevant Comorbidities:

Yersinia-Enterocolitis Mar 2020
Ulcerative Colitis
Acute Kidney Injury Mar 2020
Arterial hypertension
Combined aortic valvular defect

Immunosuppression: yes; Mesalazin

Splenomegaly: no

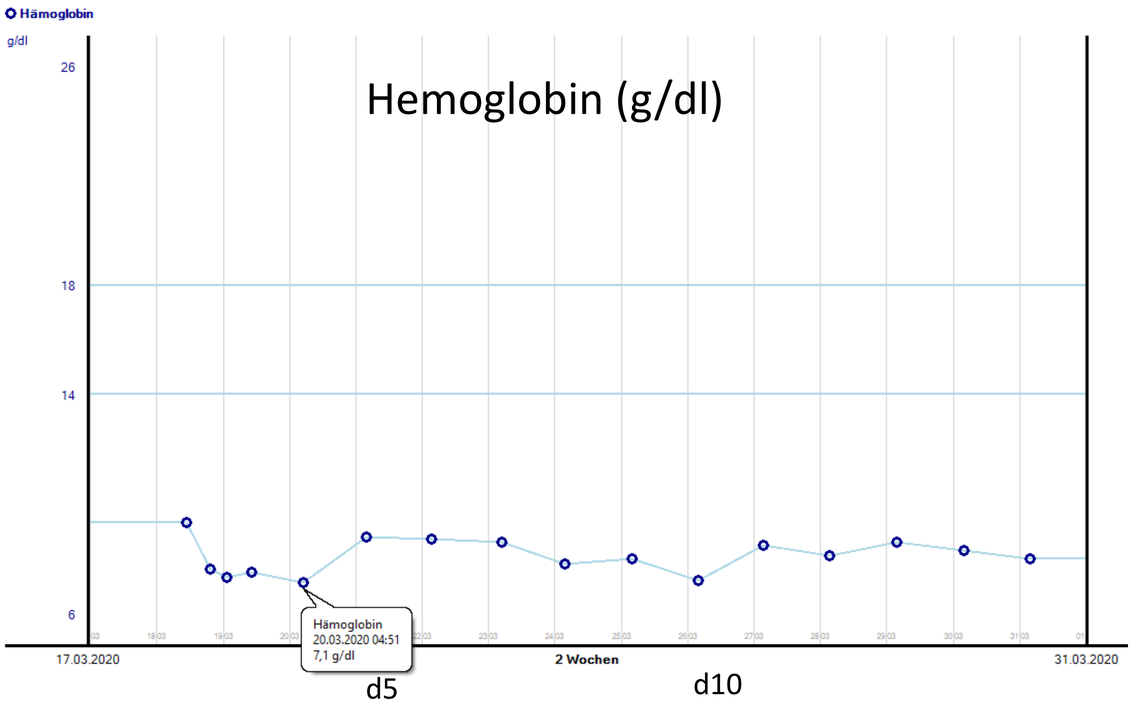

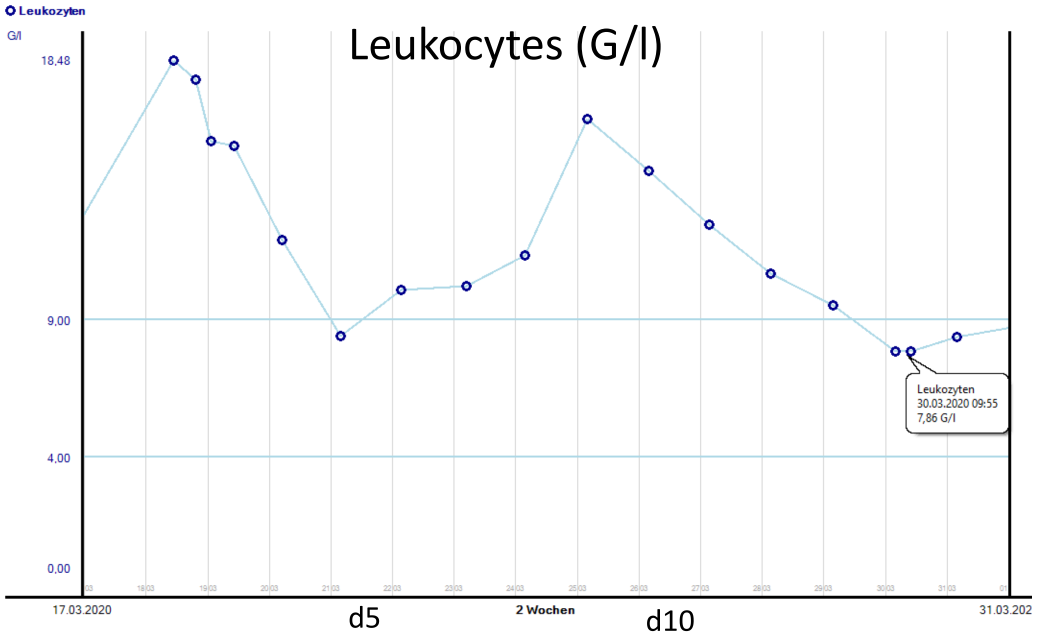

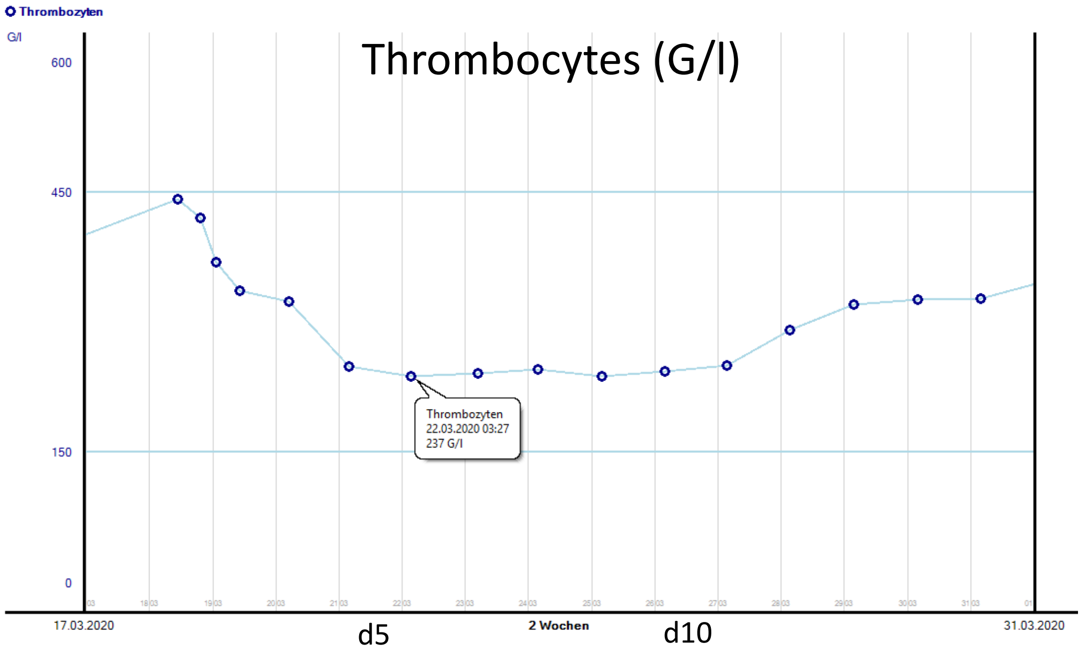

ID4; m, 84 years

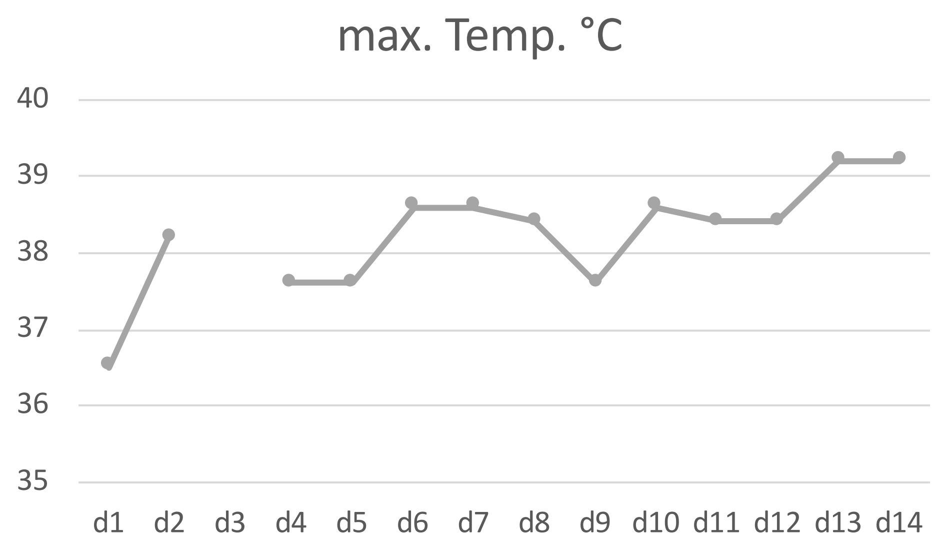

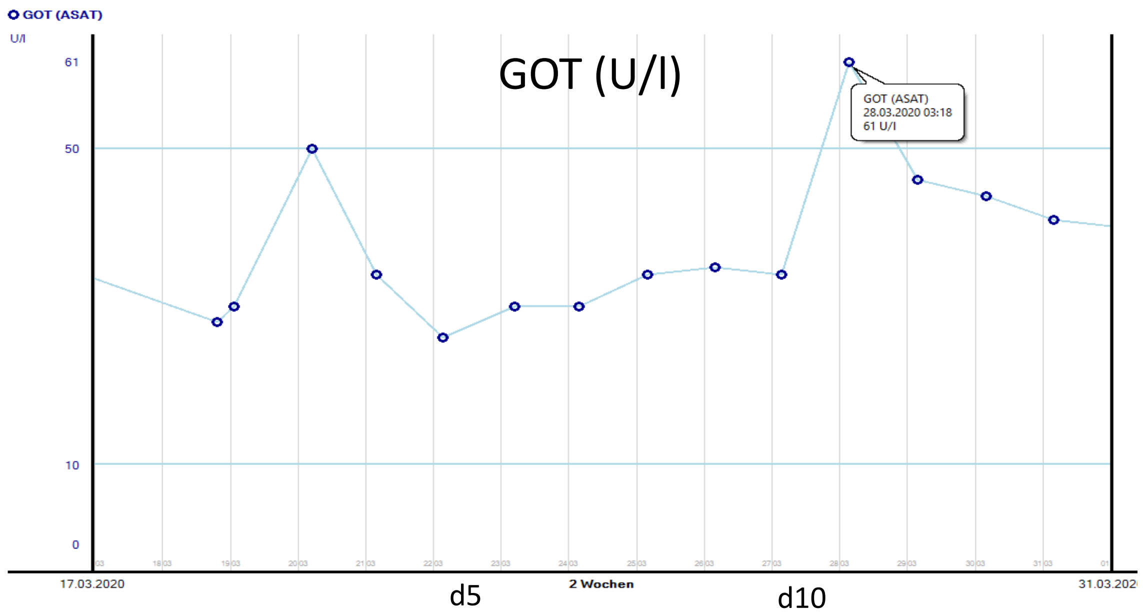

	d1	d2	d3	d4	d5	d6	d7	d8	……	d12
Ferritin (ng/ml)			3470							2527
sIL2-R (U/ml)										4855
Fibrinogen (mg/dl)				777						
Triglycerides (mg/dl)				149						
INR	1.1			1.1			1.3			
D-Dimer (µg/l FEU)				3522				4040	4063 (d9)	
Bilirubin (mg/dl)	0.2			0.3			0.4			
Procalcitonin (ng/ml)	0.5			0.4			0.9			

ID5; m, 70 years

Relevant comorbidities:
Atrial fibrillation
Arterial hypertension
Cerebrovascular disease
polyneuropathy

Immunosuppression: no

Splenomegaly: no

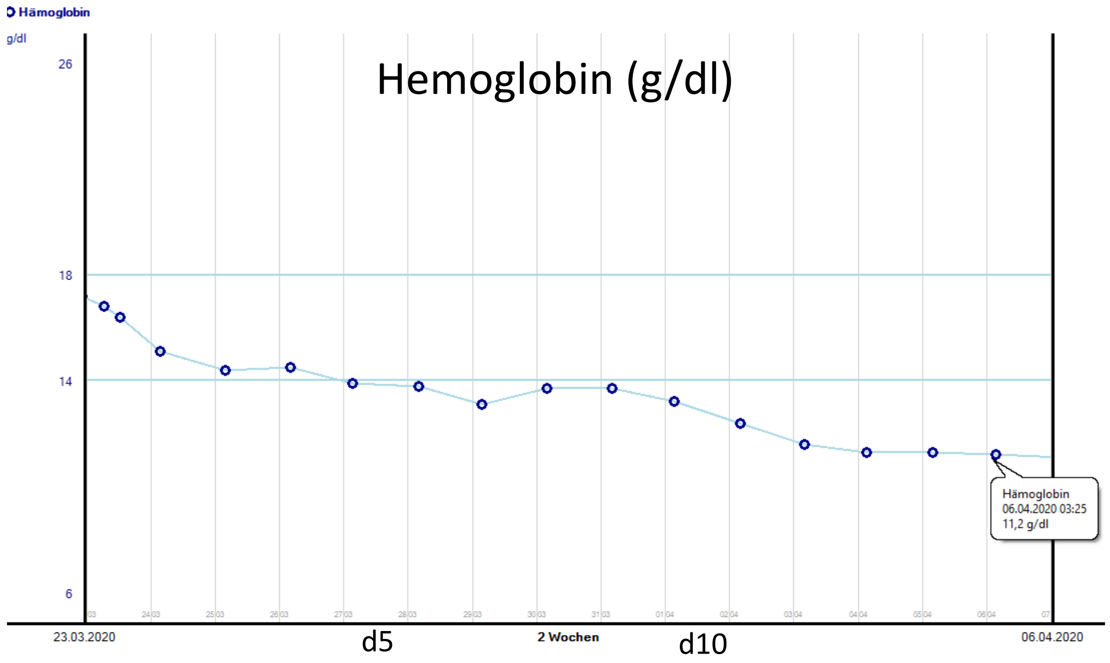

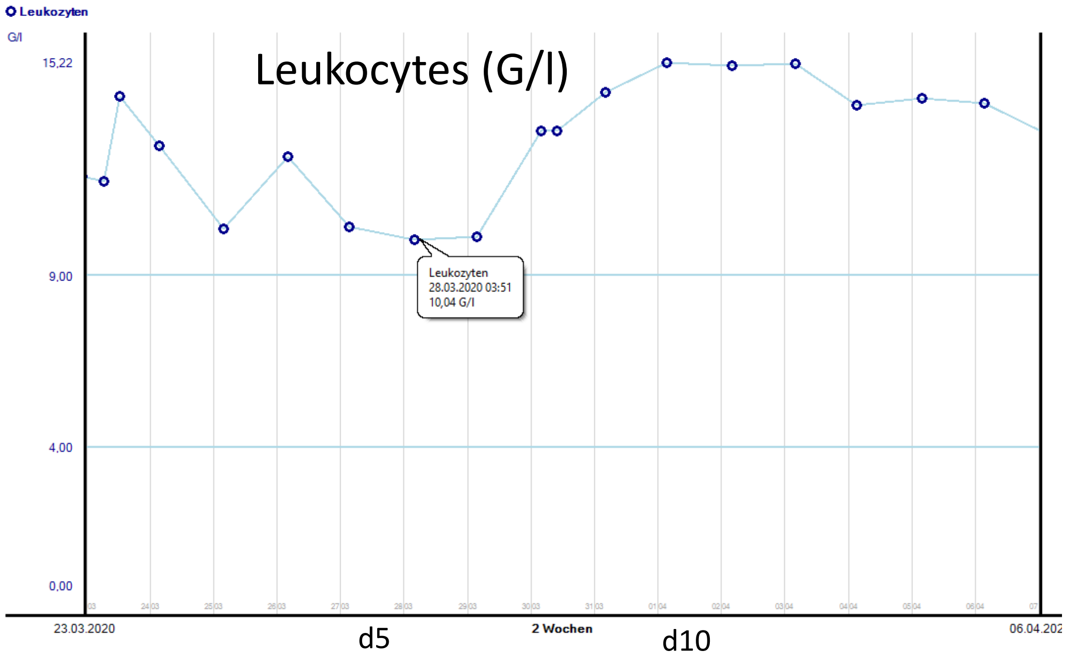

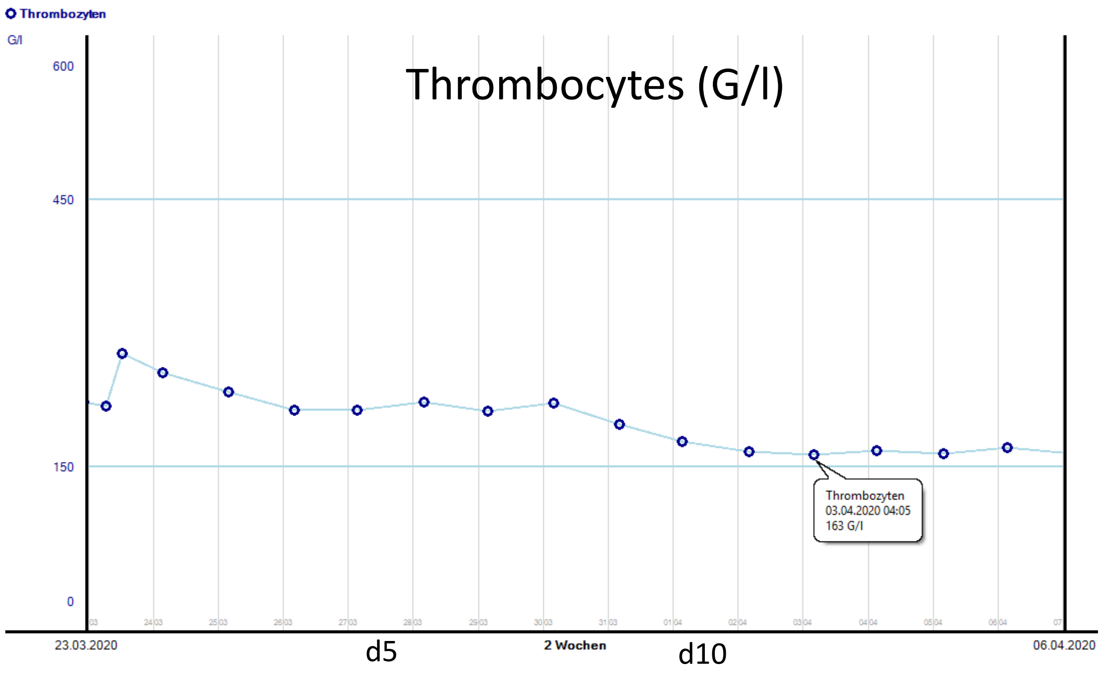

ID5; m, 70 years

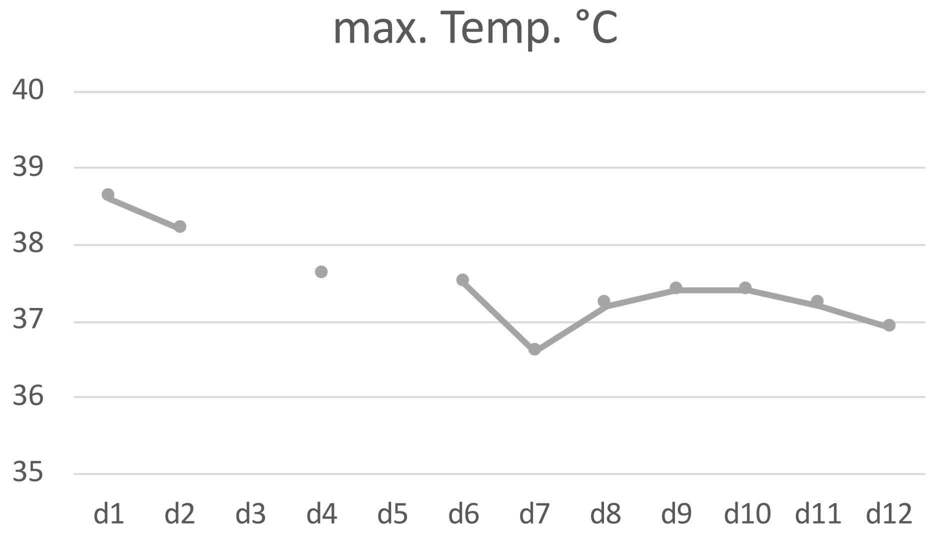

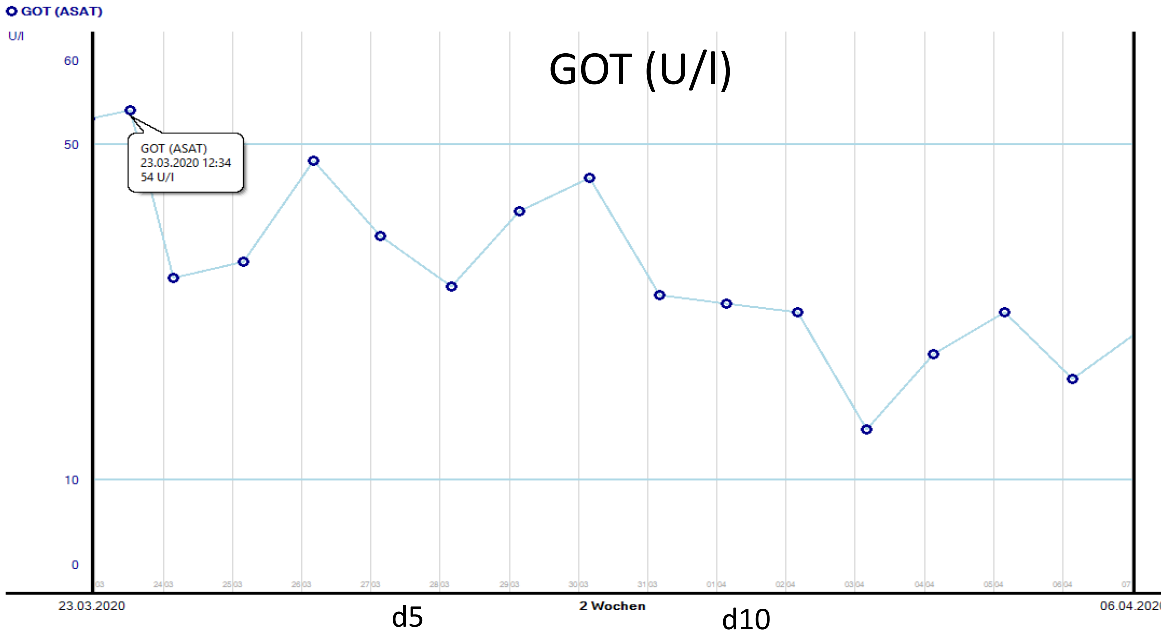

	d1	d2	d3	d4	d5	d6	d7	d8	d9	d10
Ferritin (ng/ml)					2115			1143		
sIL2-R (U/ml)								1105		
Fibrinogen (mg/dl)					752			729		
Triglycerides (mg/dl)					135					
INR	1.5			1.1			1.1			
D-Dimer (µg/l FEU)			2801	2508				2124		
Bilirubin (mg/dl)	1			0.8			0.6			
Procalcitonin (ng/ml)	0.2			0.8				0.2		

ID 6; m, 52 years

Relevant comorbidities:

cirrhosis
Condition after gastrointestinal stromal tumor
Condition after Duodenal ulcer mit Perforation
Arterial hypertension
Diabetes mellitus
Chronic kidney disease

Immunosuppression: no

Splenomegaly: yes

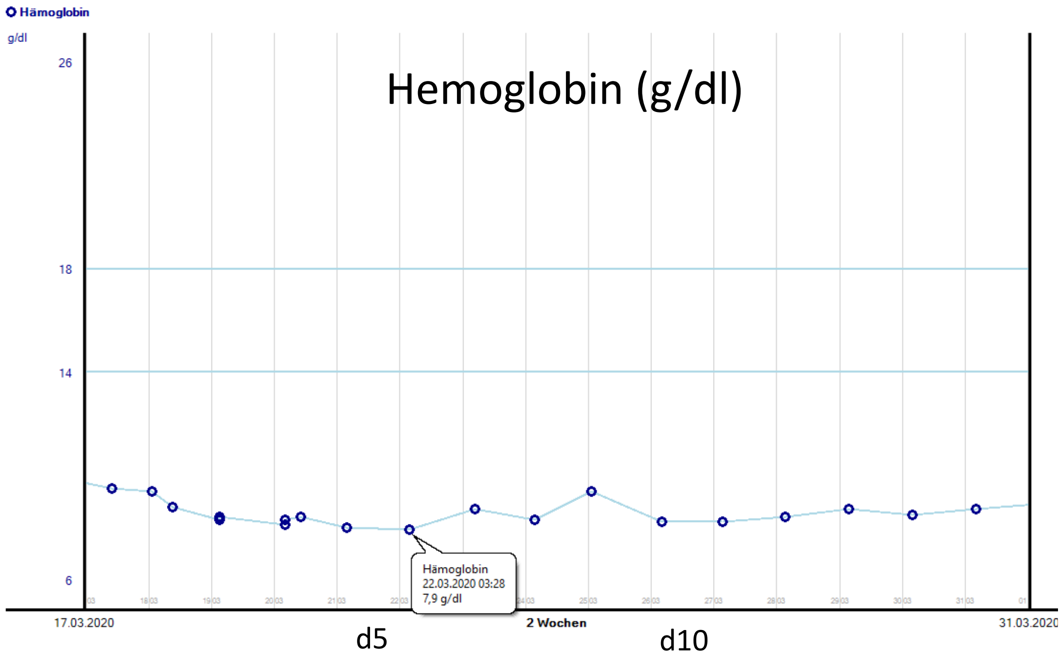

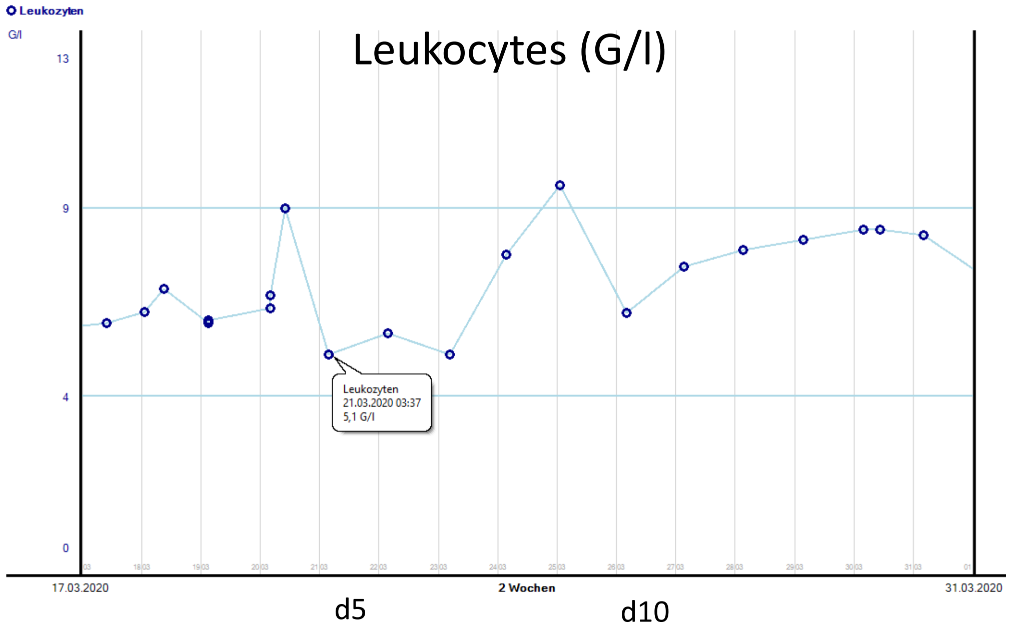

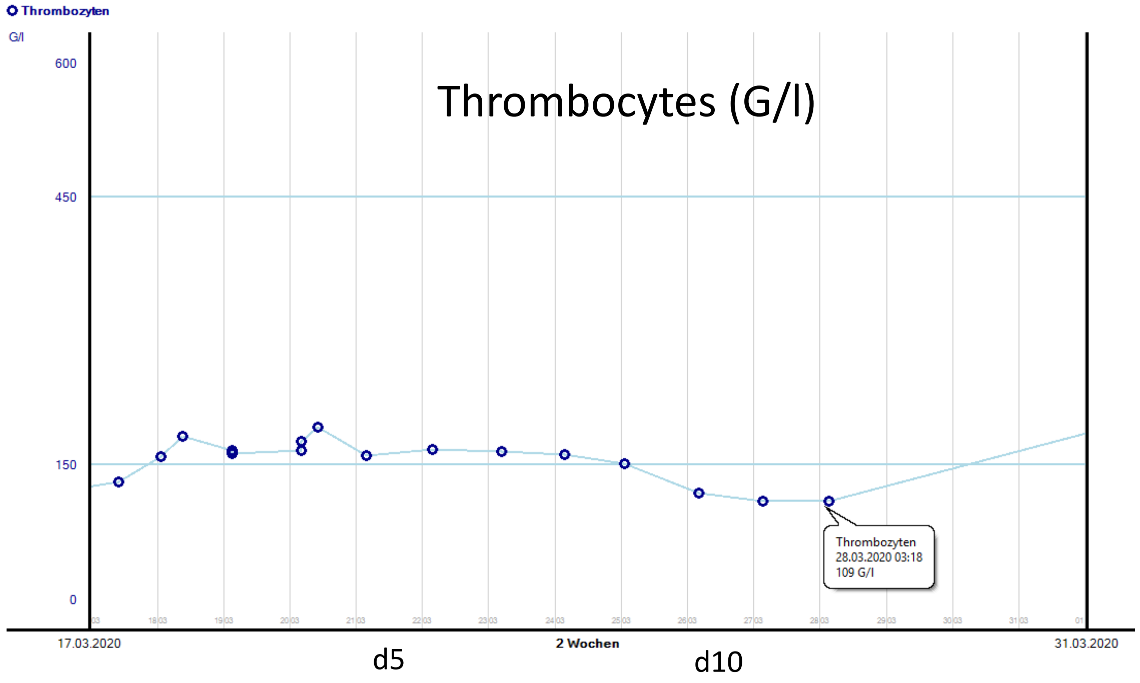

ID 6; m, 52 years

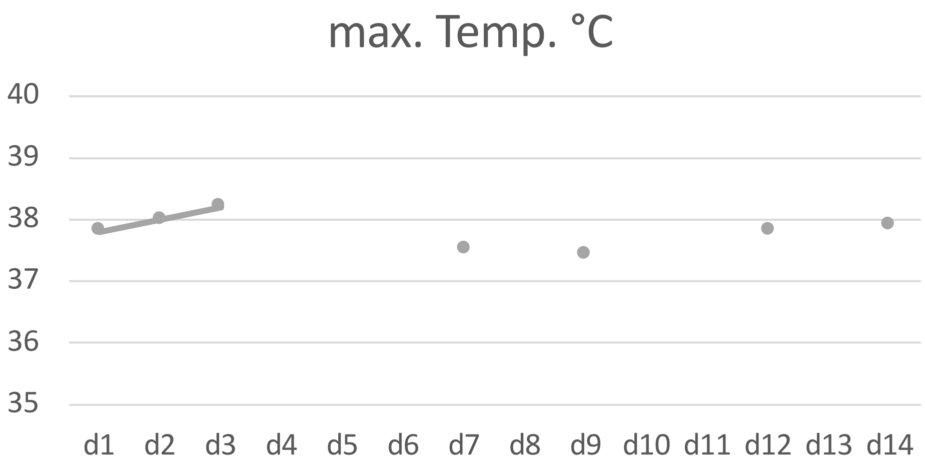

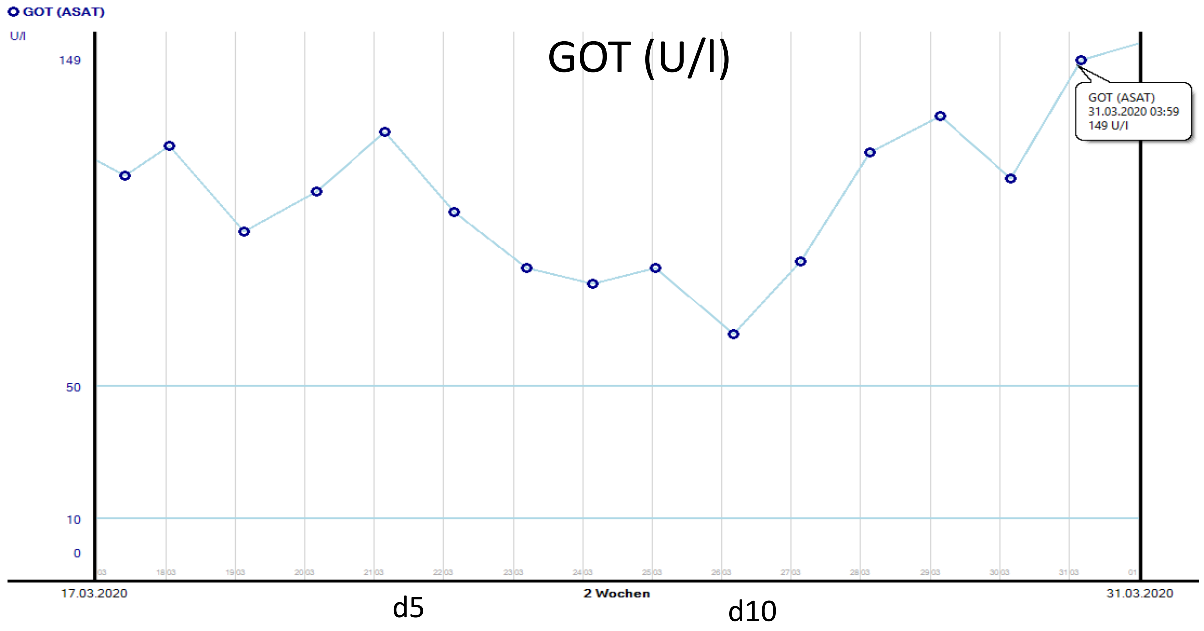

	d1	d2	d3	d4	d5	.....	d12	....	d16	...
Ferritin (ng/ml)			816				637		919 (max)	
sIL2-R (U/ml)							834		653	
Fibrinogen (mg/dl)				691			572			
Triglycerides (mg/dl)			280		428					
INR	1.0			1.0		1.0 (d8)				
D-Dimer (µg/l FEU)						868 (d10)		702 (d13)		
Bilirubin (mg/dl)	0.4			0.5		0.6 (d8)				
Procalcitonin (ng/ml)	0.7			0.8		0.5 (d8)				

ID 7; m, 54 years

Relevant Comorbidities:
none

Immunosuppression: no

Splenomegaly: yes

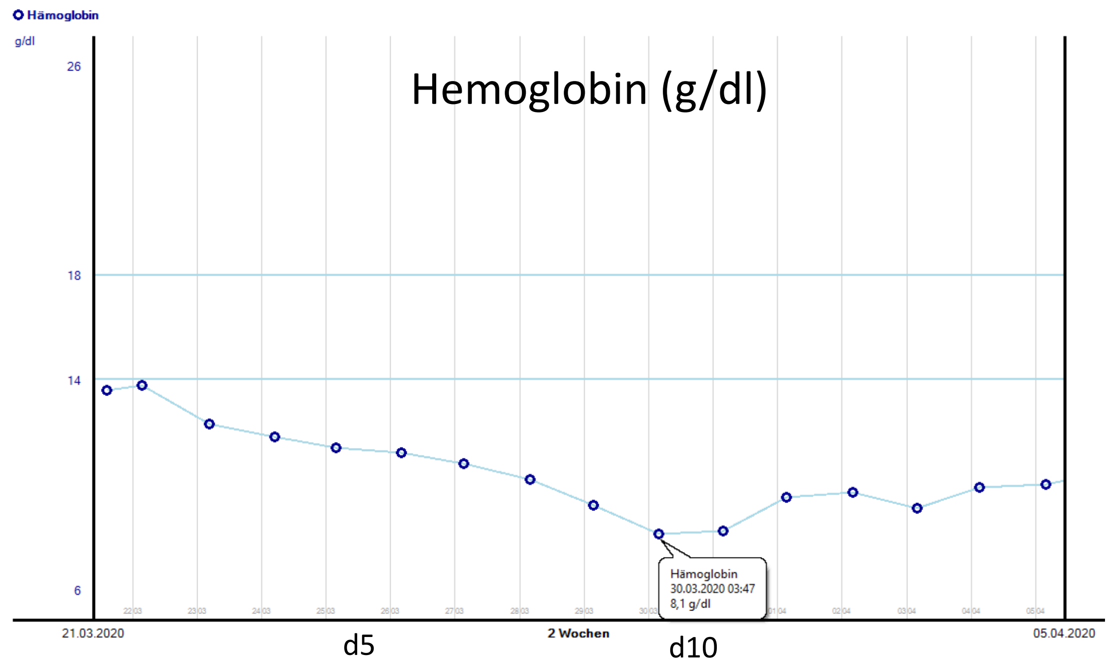

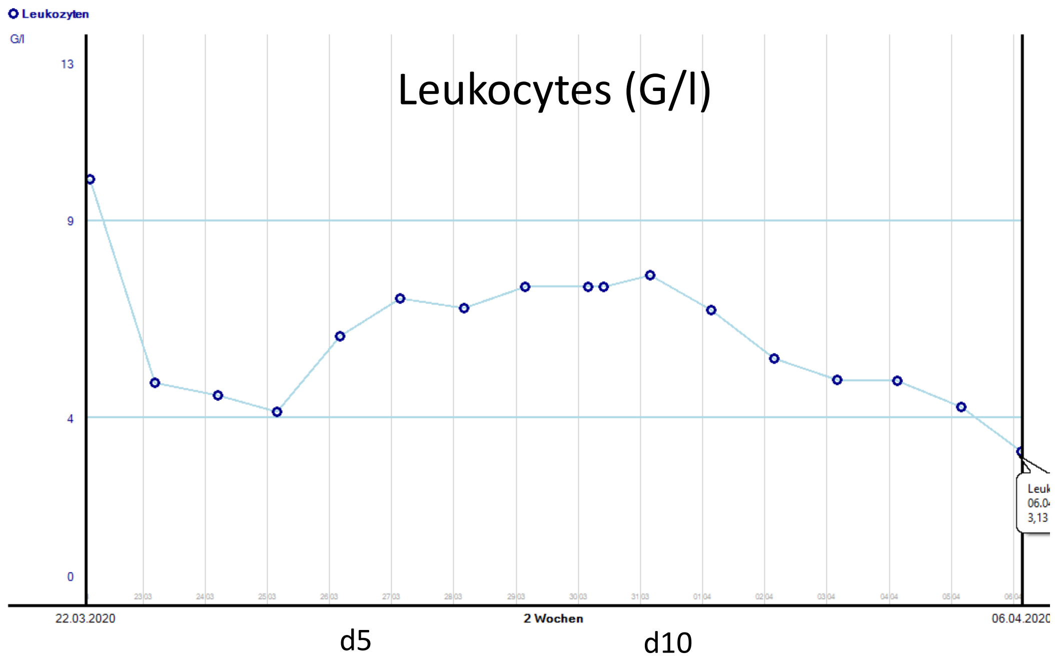

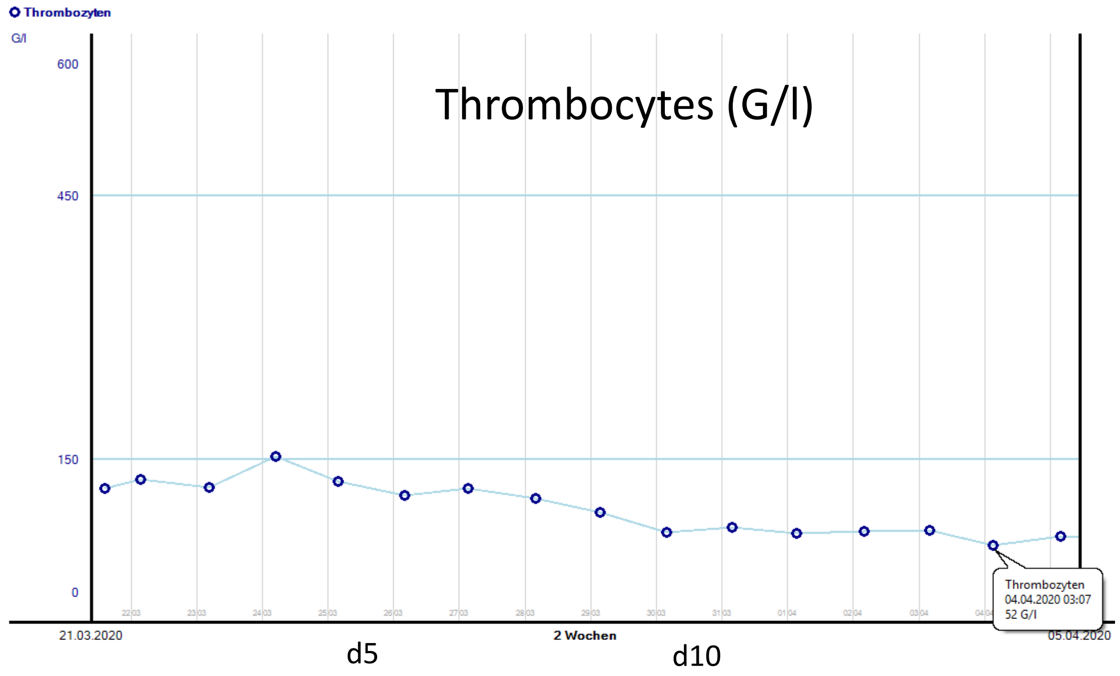

ID 7; m, 54 years

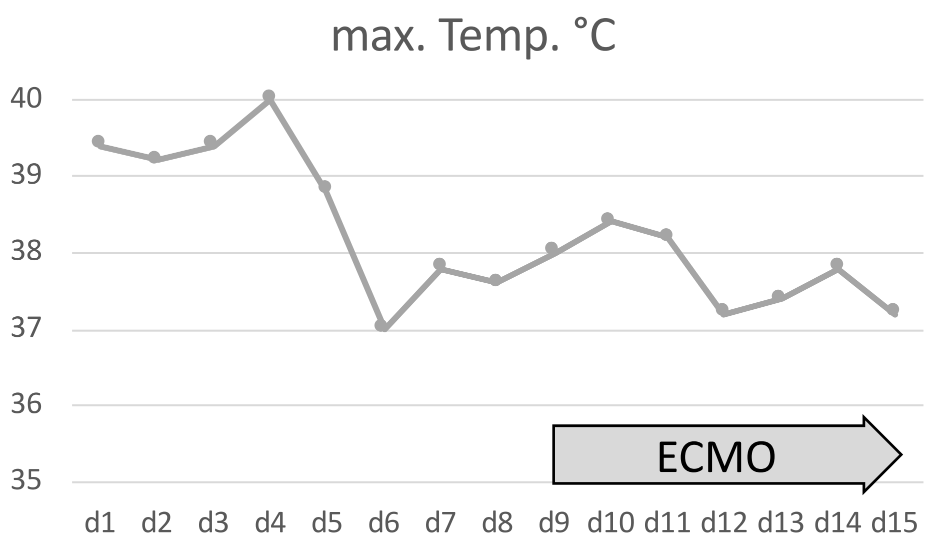

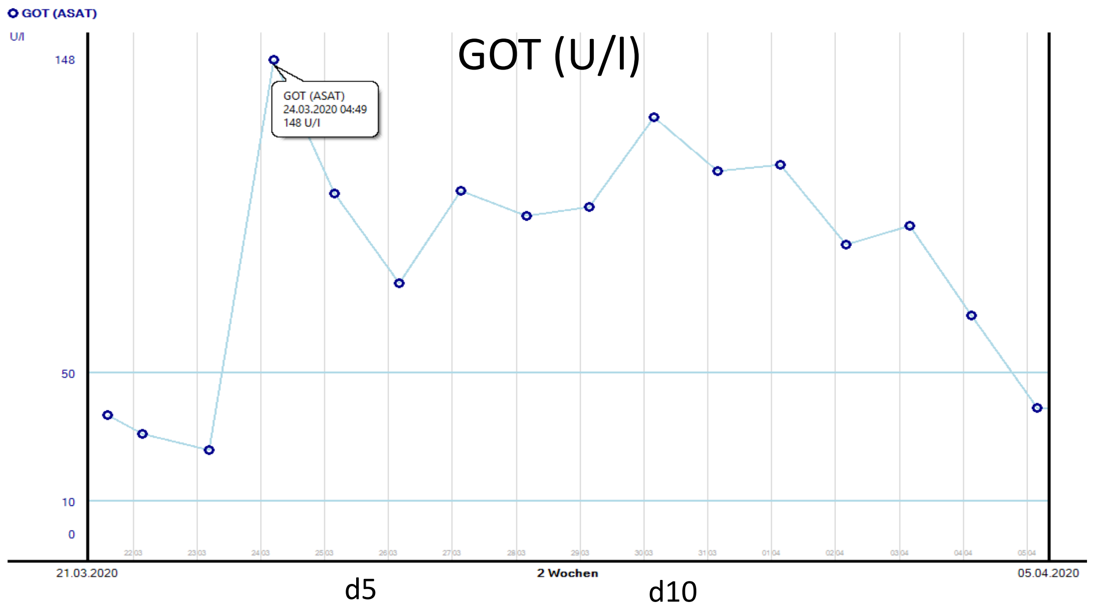

	d1	d2	d3	....	d7	d8	....	d10	....	d13
Ferritin (ng/ml)			1225		2775	2501		3343		4366
sIL2-R (U/ml)								1174		
Fibrinogen (mg/dl)	892				642			502		
Triglycerides (mg/dl)					282			240		
INR	1.1		1.1		1.2					
D-Dimer (µg/l FEU)	1701		129030		56865					
Bilirubin (mg/dl)	0.8		0.4		0.8					
Procalcitonin (ng/ml)	1.0		0.8		3.6					

Superinfection: positive blood culture for Staph. epid. d7

ID 8; m, 83 years

Relevant comorbidities:
Atrial fibrillation
Pancytopenia of unknown origin (not classified)

Immunosuppression: no

Splenomegaly: no

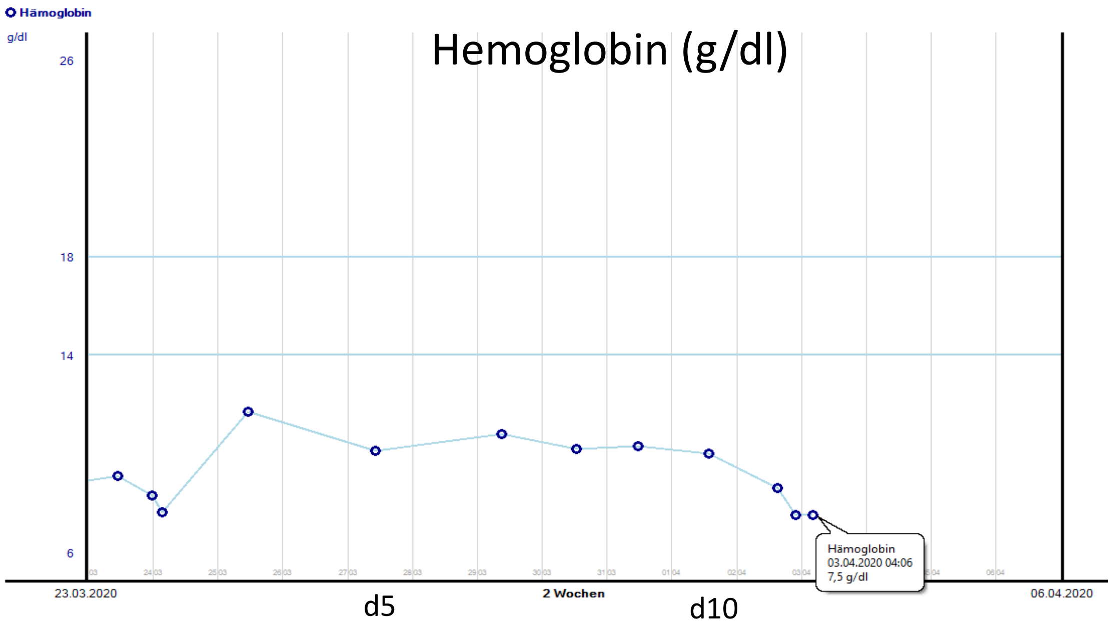

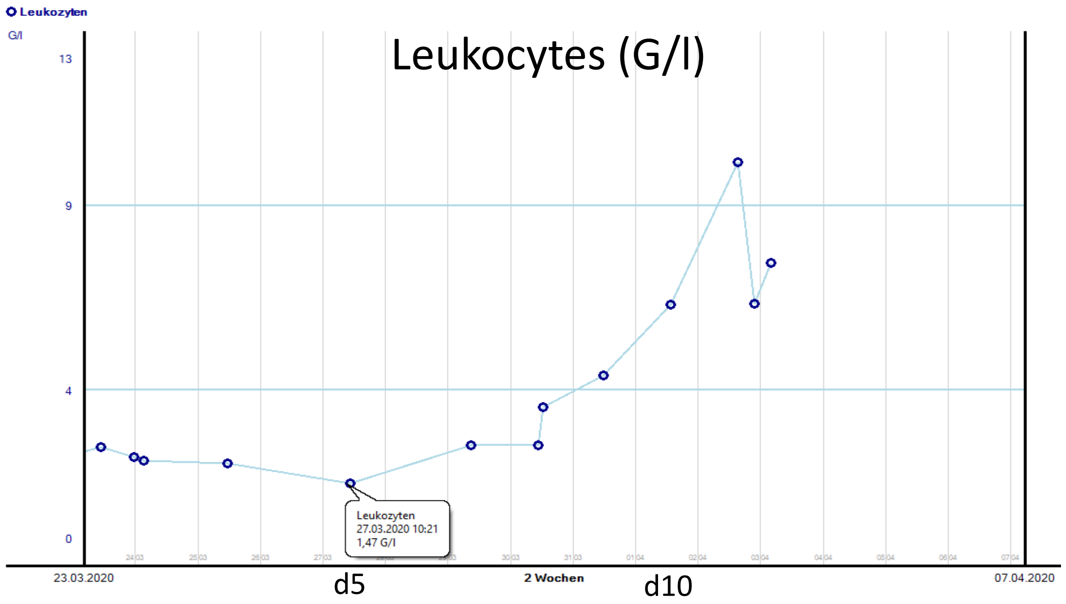

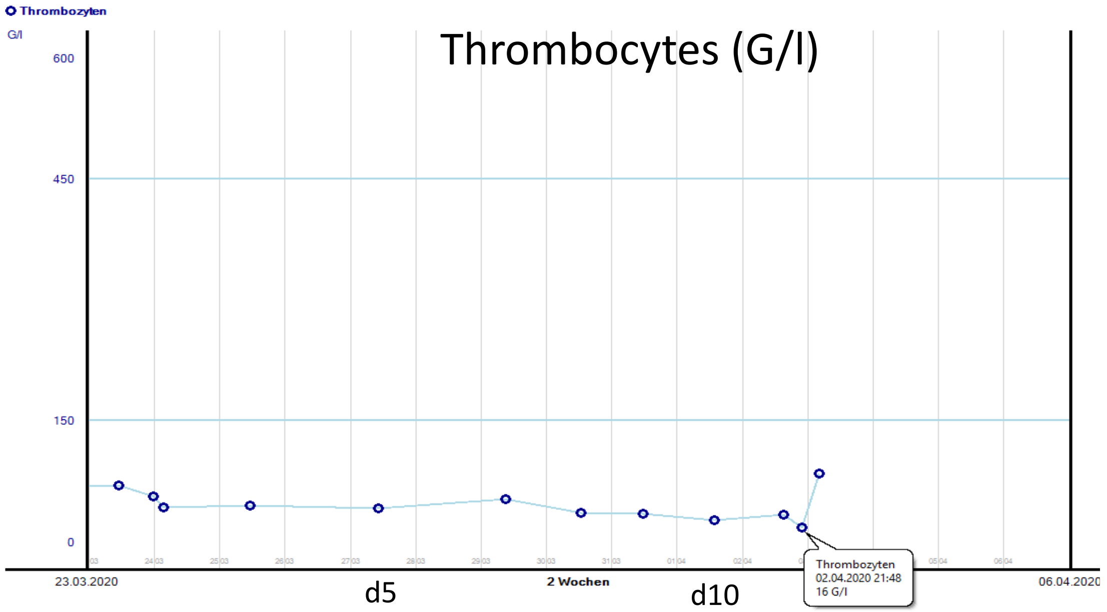

ID 8; m, 83 years

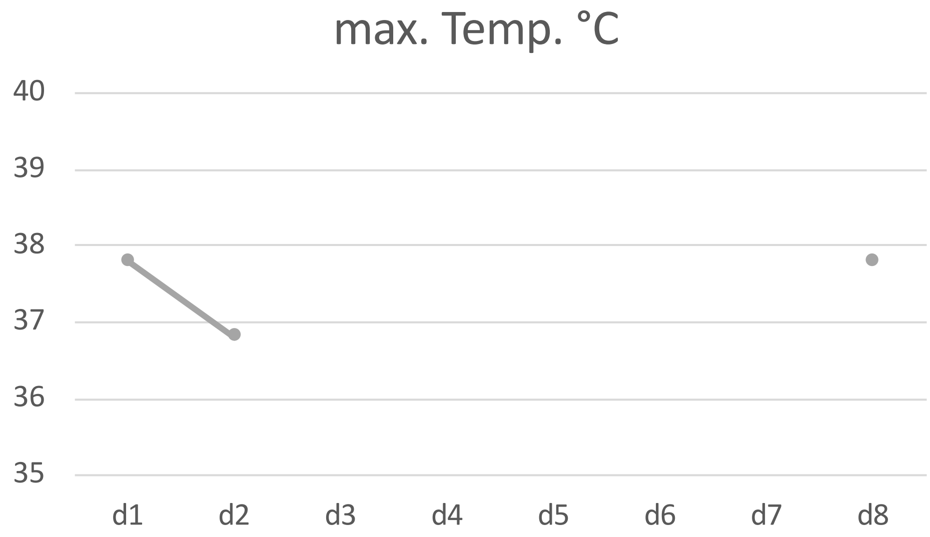

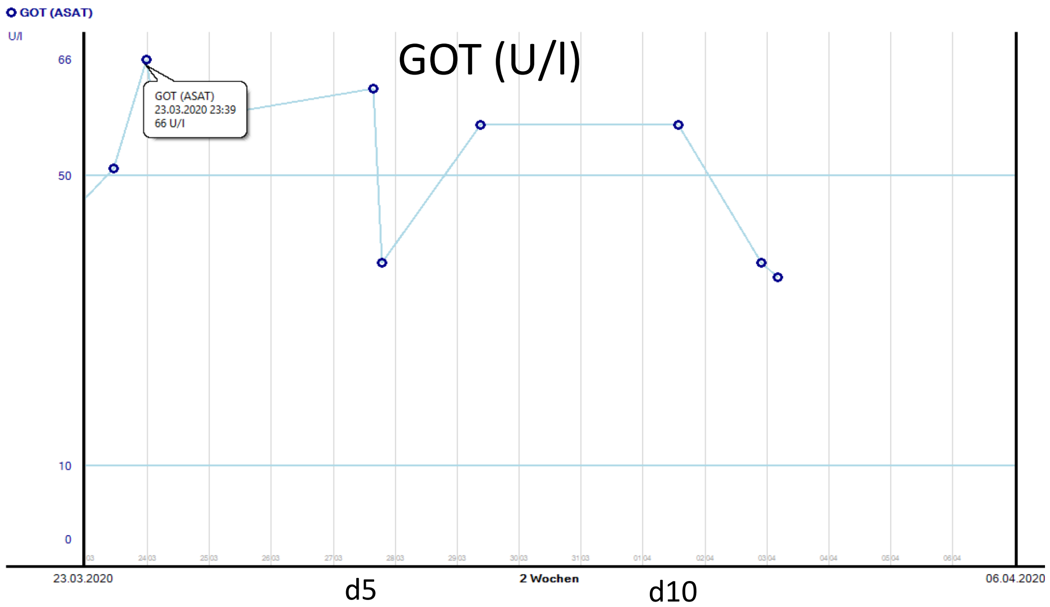

	d1	d2	d3	d4	d5	d6	d7	d8	d9	d12
Ferritin (ng/ml)										5059
sIL2-R (U/ml)								967		
Fibrinogen (mg/dl)										471
Triglycerides (mg/dl)										219
INR	2.3			1.9				1.6		
D-Dimer (µg/l FEU)									1840	
Bilirubin (mg/dl)	0.6			0.6		0.7				
Procalcitonin (ng/ml)	0.2			0.1				0.2		

ID 9; m, 72 years

Relevant comorbidities:
Atrial fibrillation
Arterial hypertension

Immunosuppression: no

Splenomegaly: no

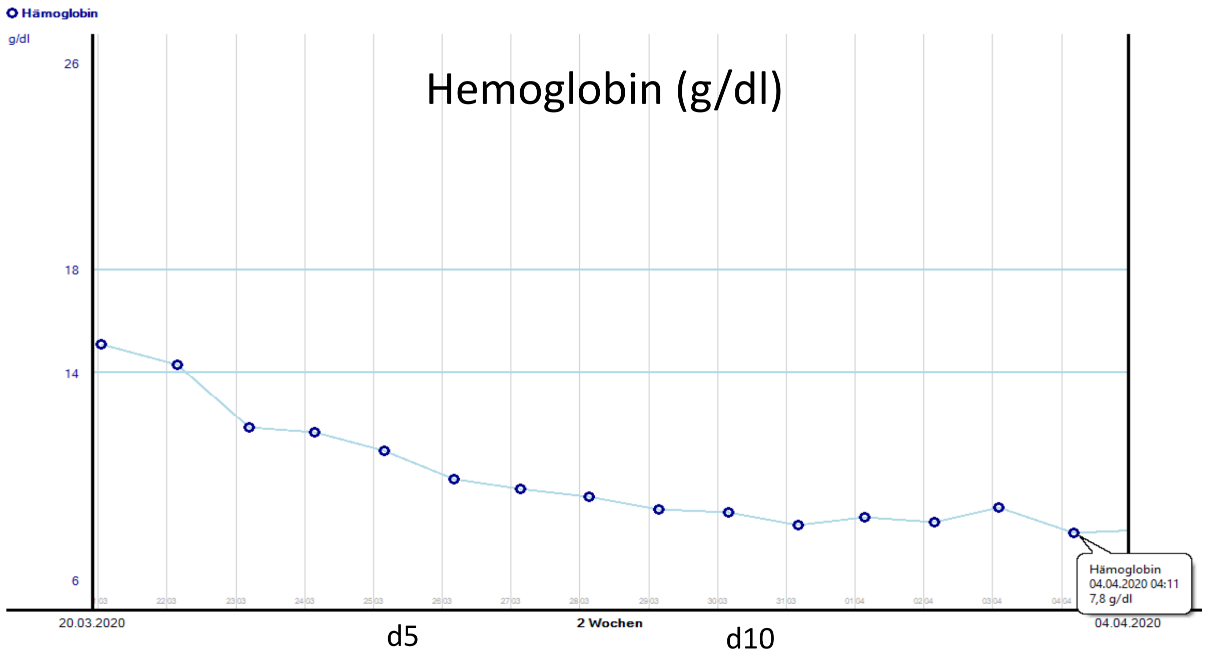

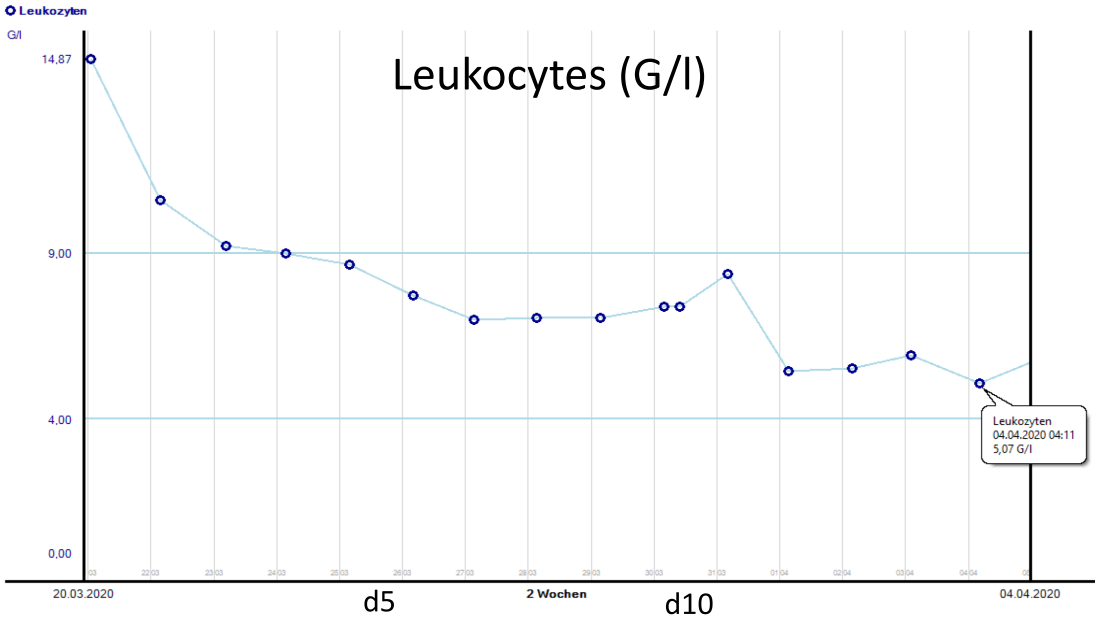

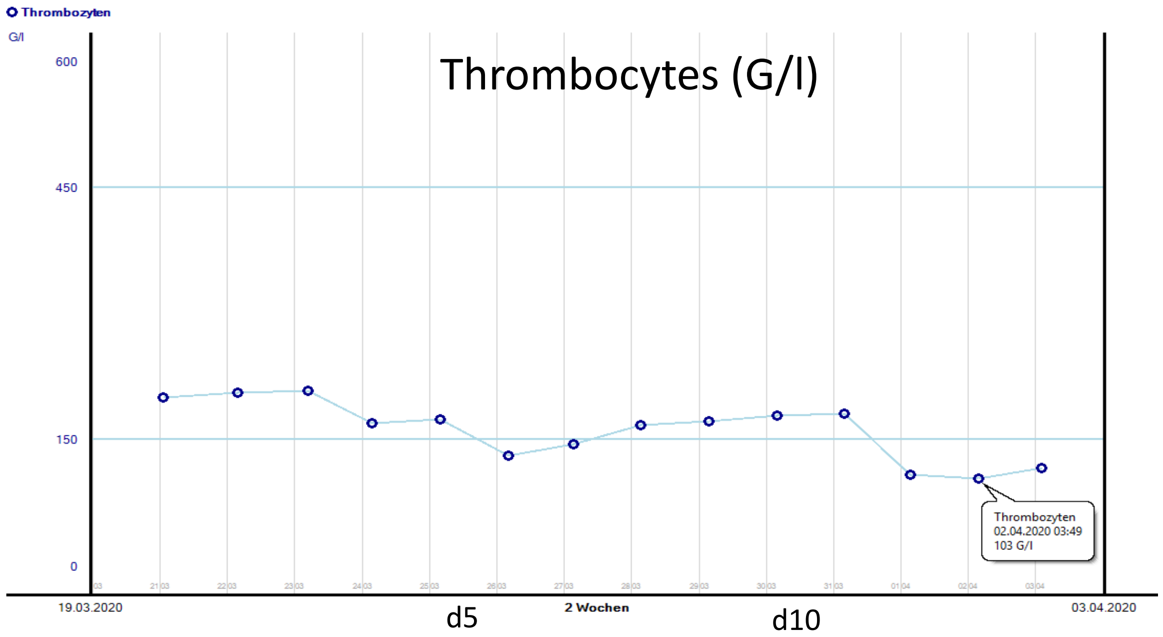

ID 9; m, 72 years

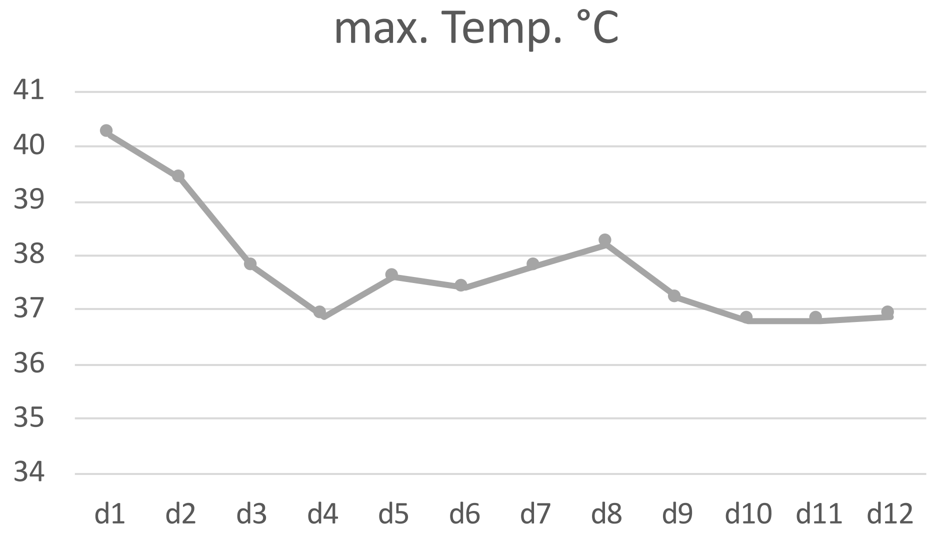

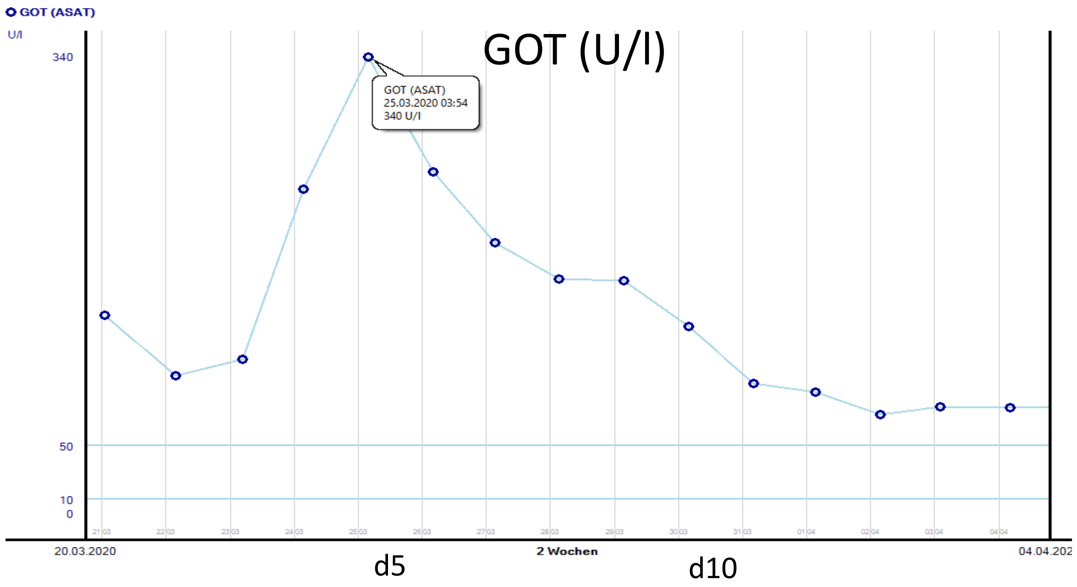

	d1	d2	d3	d4	d5	d6	…	d10	….	d13
Ferritin (ng/ml)								6196		
sIL2-R (U/ml)								3862		
Fibrinogen (mg/dl)		1028								867
Triglycerides (mg/dl)		218								
INR	1.2						1.0 (d7)			
D-Dimer (µg/l FEU)	1281				1374		2093 (d7)			
Bilirubin (mg/dl)	0.8			2.8			8.9 (d7)			
Procalcitonin (ng/ml)	4.9			8.2			2.5 (d7)			

Superinfection: culture negative

ID 10; m, 78 years

Relevant comorbidities:
Recent acute kidney injury (NSAID induced)

Immunosuppression: no

Splenomegaly: no

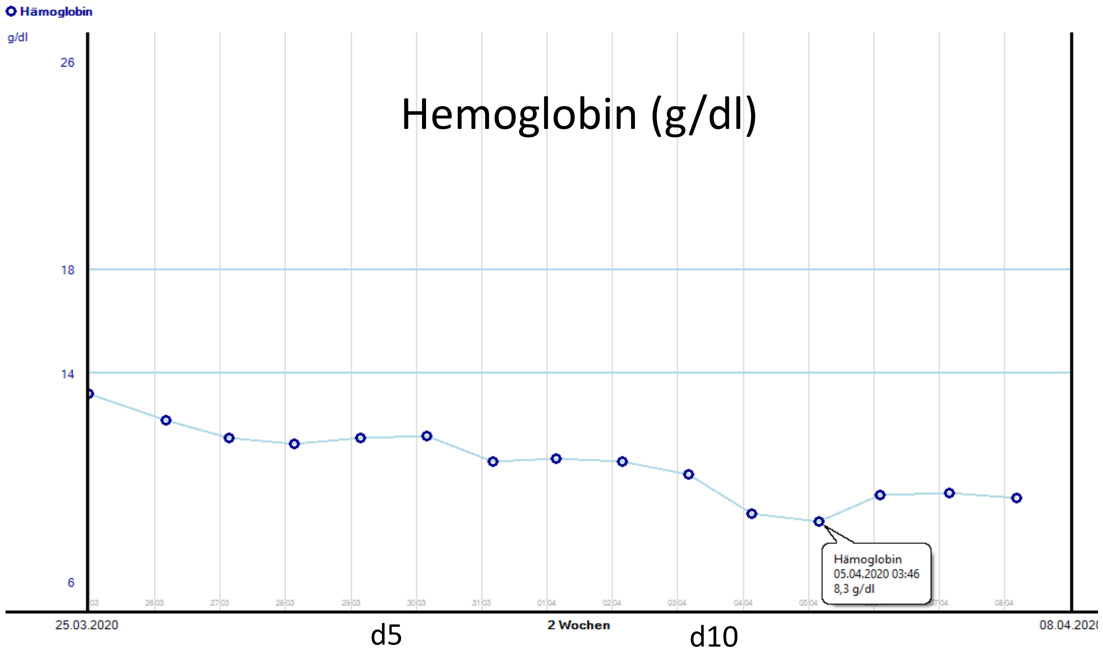

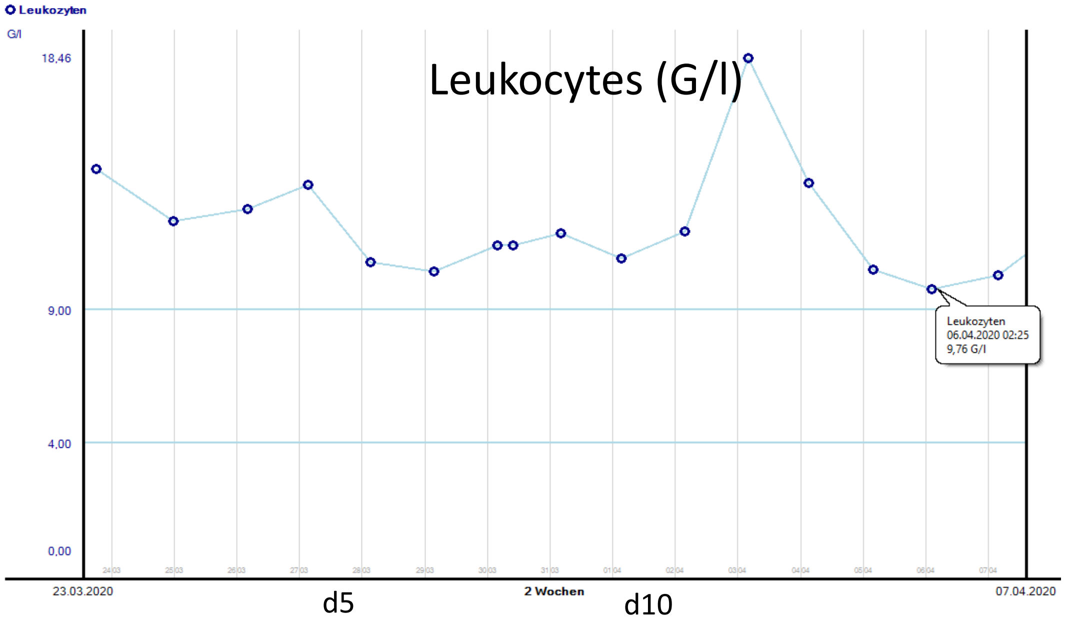

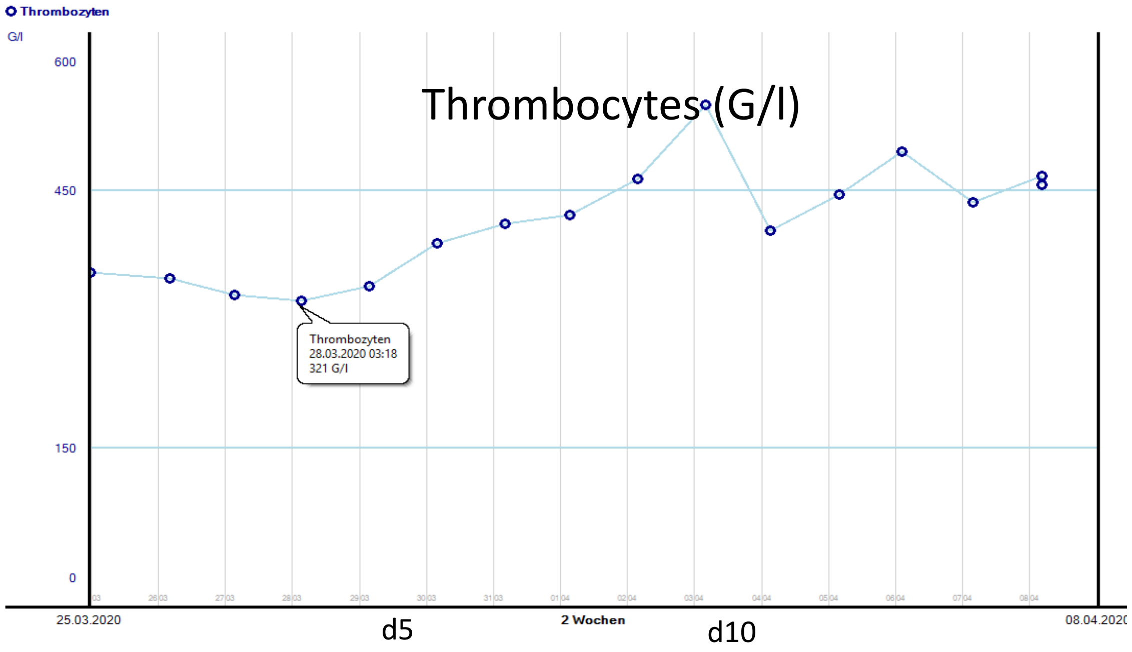

ID 10; m, 78 years

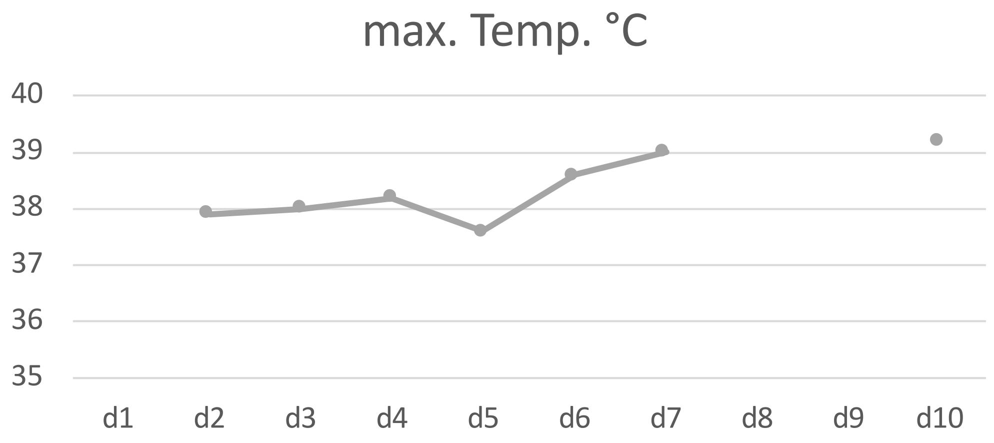

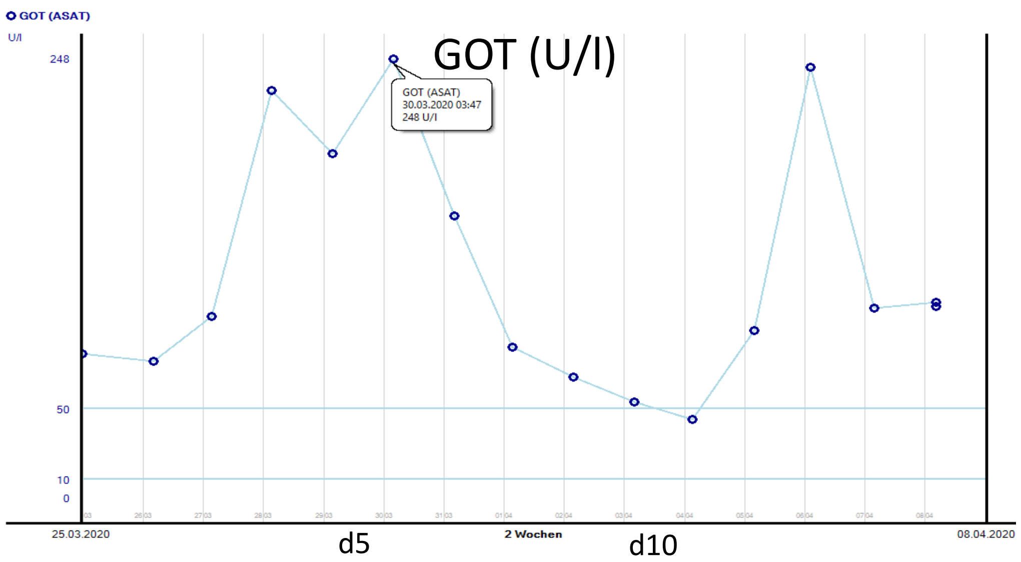

	d1	d2	d3	d4	d5	d6	d7	d8	d9	d10
Ferritin (ng/ml)				8875			5159			4674
sIL2-R (U/ml)							2129			
Fibrinogen (mg/dl)				823				591		
Triglycerides (mg/dl)				414						
INR	1.1			1.0			1.1			
D-Dimer (µg/l FEU)	3145		1891					7076		
Bilirubin (mg/dl)	0.7			0.4			0.3			
Procalcitonin (ng/ml)	1.4		1.6					0,6		

Superinfection: positive blood culture for Staph. Aureus on d3

ID 11; m, 53 years

Relevant comorbidities:
Ulcerative colitis
Multiple endocrine Neoplasie Type I
Diabetes mellitus

Immunosuppression: yes; Cyclosporin, Budesonid

splenomegaly: History of splenectomy

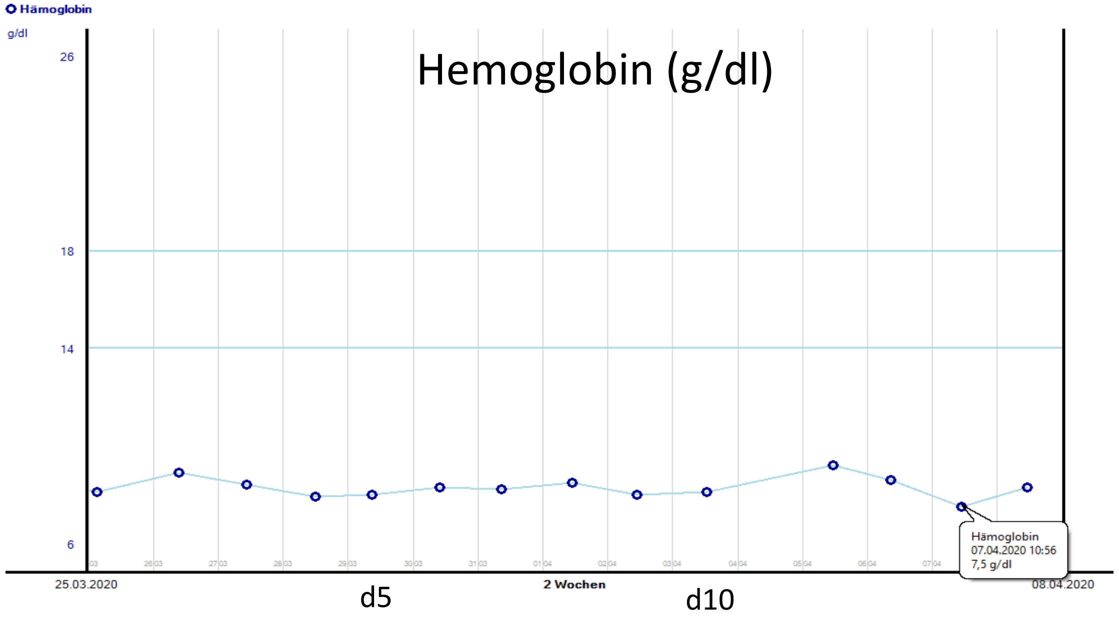

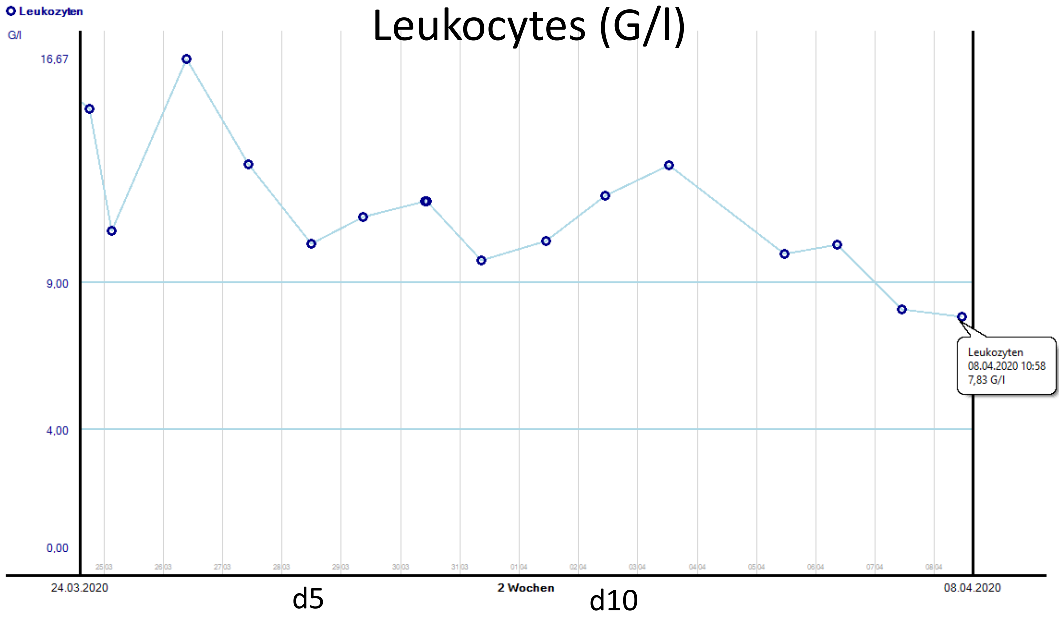

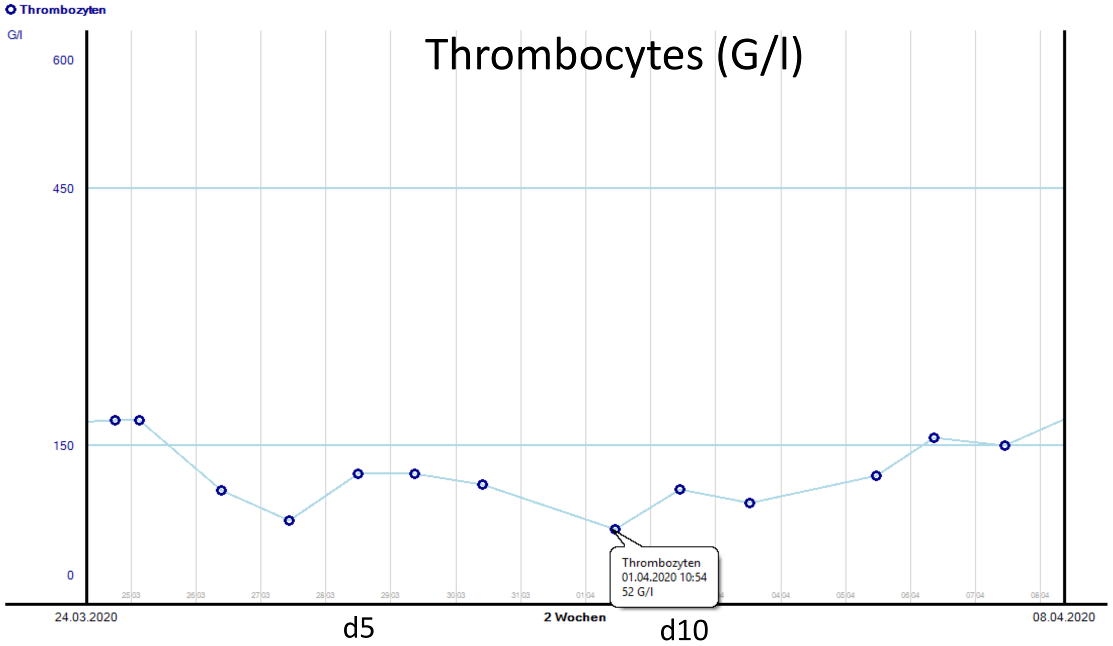

ID 11; m, 53 years

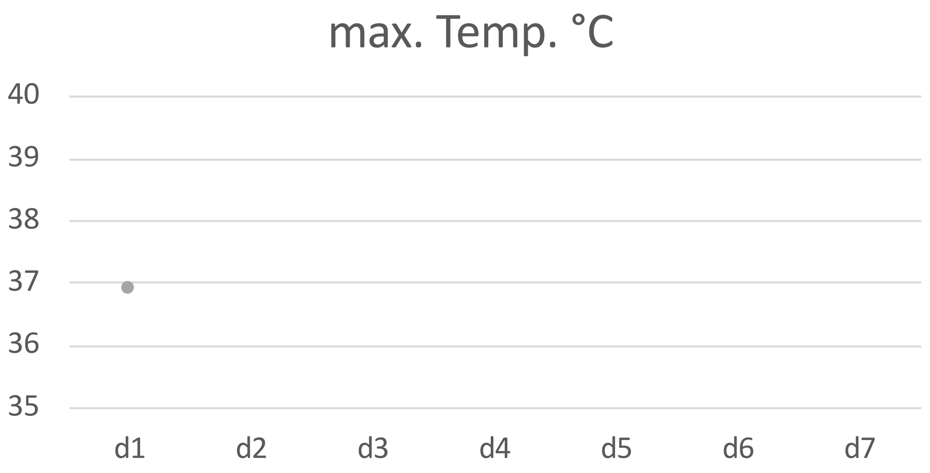

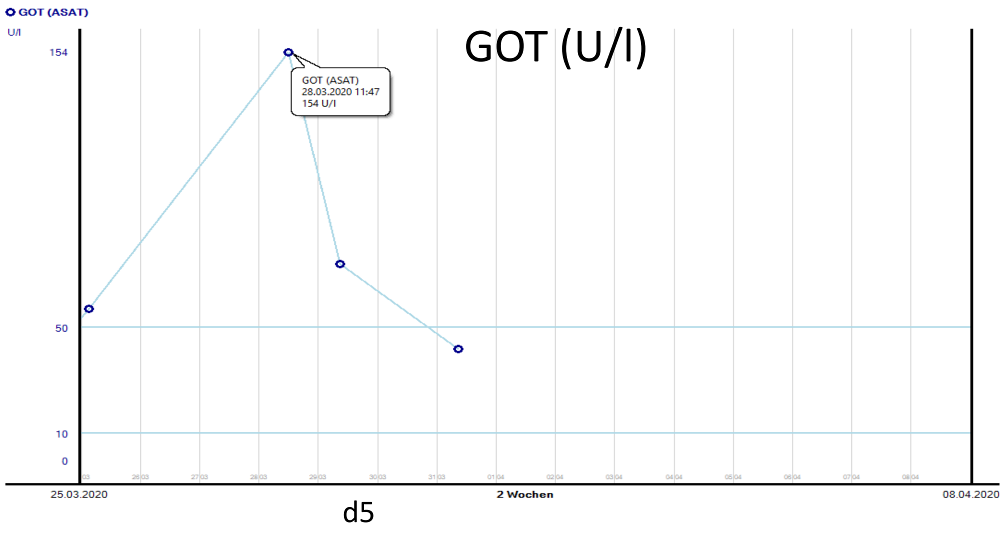

	d1	d2	d3	d4	d5	d6	d7	d8	d9	d10
Ferritin (ng/ml)			1322						288	
sIL2-R (U/ml)							2290			
Fibrinogen (mg/dl)			811							
Triglycerides (mg/dl)			361							
INR	1.0	1.0						0.9		
D-Dimer (µg/l FEU)	6548	6226								
Bilirubin (mg/dl)	1	1					1.6			
Procalcitonin (ng/ml)	5.6	4.7					0.6			

Superinfection: positive blood culture for Pseudomonas aer. on d -3

ID 12; f, 60 years

Relevant comorbidities:
COPD
arterial hypertension
Diabetes mellitus
Hypertensive heart disease

Immunosuppression: no

Splenomegaly: no

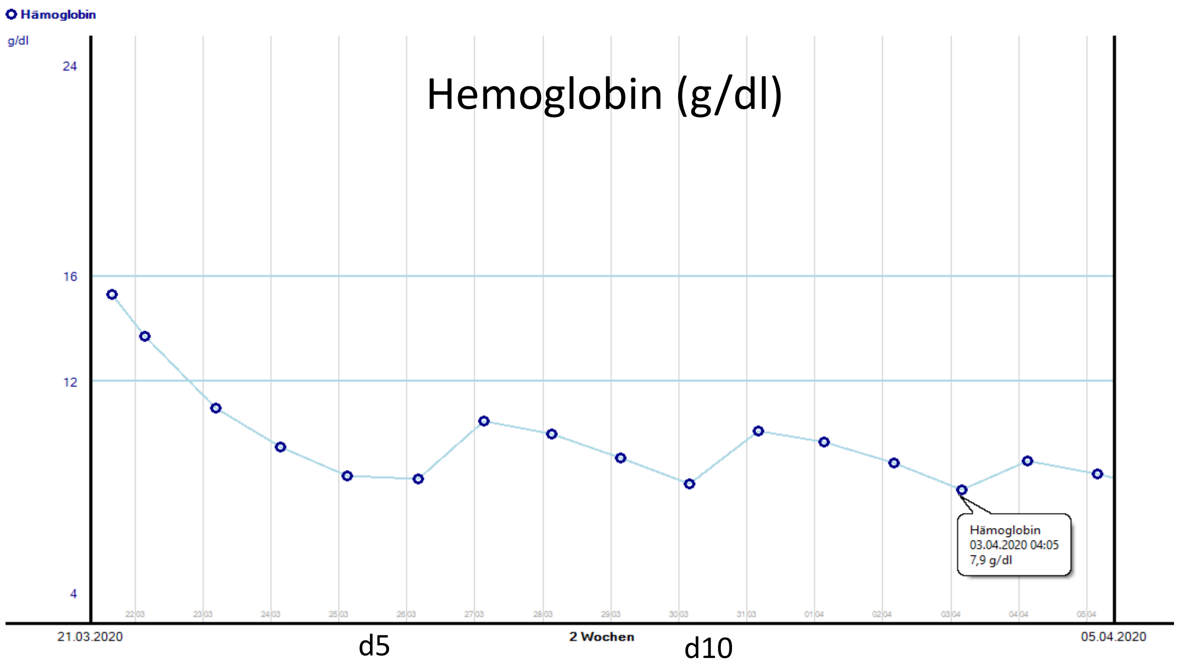

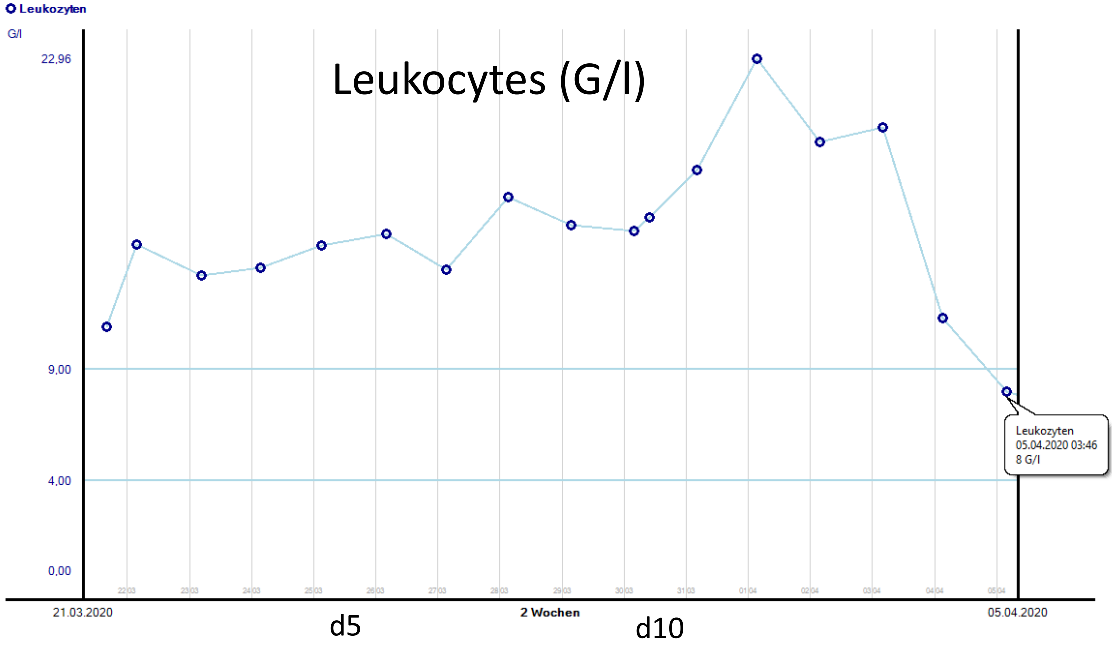

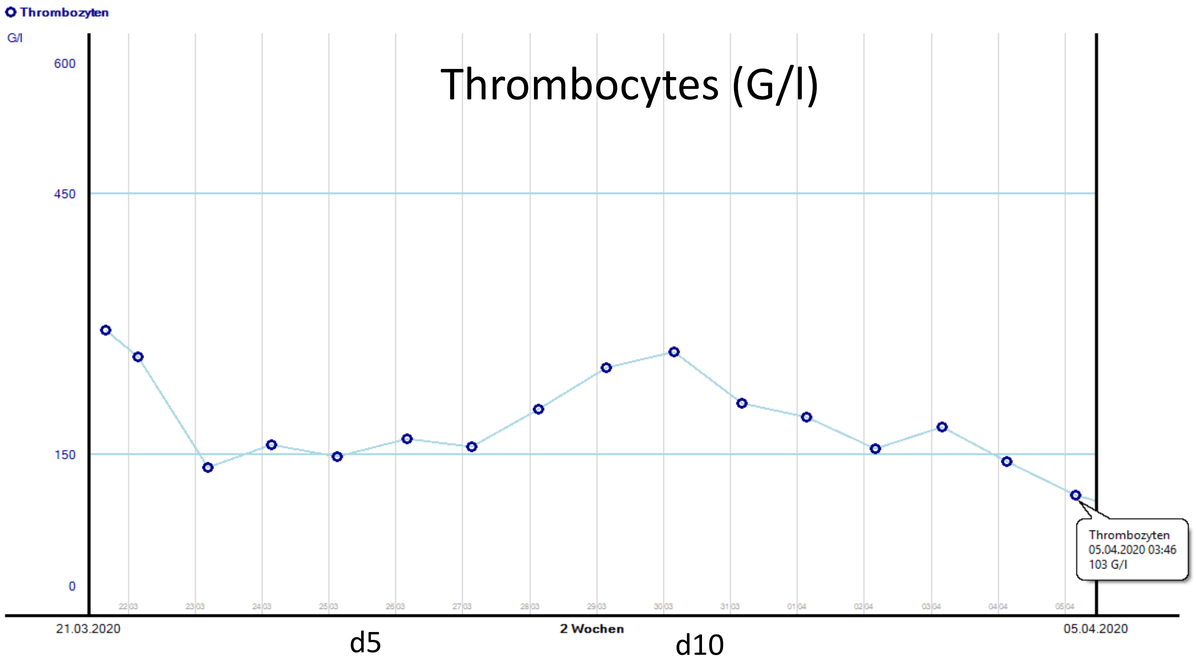

ID 12; f, 60 years

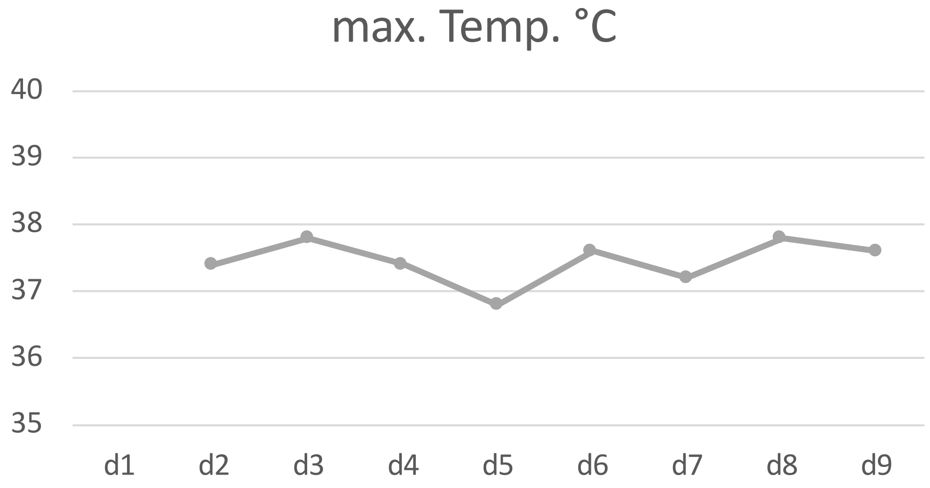

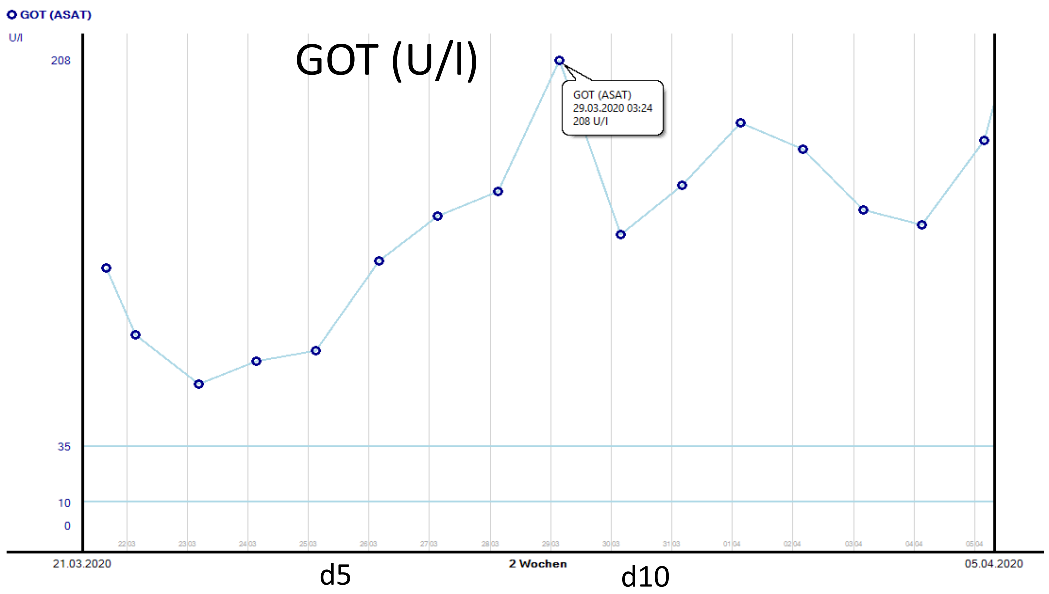

	d1	d2	d3	d4	d5	d6	d7	d8	d9	d10
Ferritin (ng/ml)			2196							3674
sIL2-R (U/ml)										1406
Fibrinogen (mg/dl)	549									649
Triglycerides (mg/dl)	136									
INR	0.9				1.0			1.0		
D-Dimer (µg/l FEU)	6640				14151		9618			
Bilirubin (mg/dl)	0.4				0.4			0.5		
Procalcitonin (ng/ml)	0.8				0.4		0,5			

ID 13; m, 59 years

Relevant comorbidities:
Diabetes mellitus
arterial hypertension

Immunosuppression: no

Splenomegaly: no

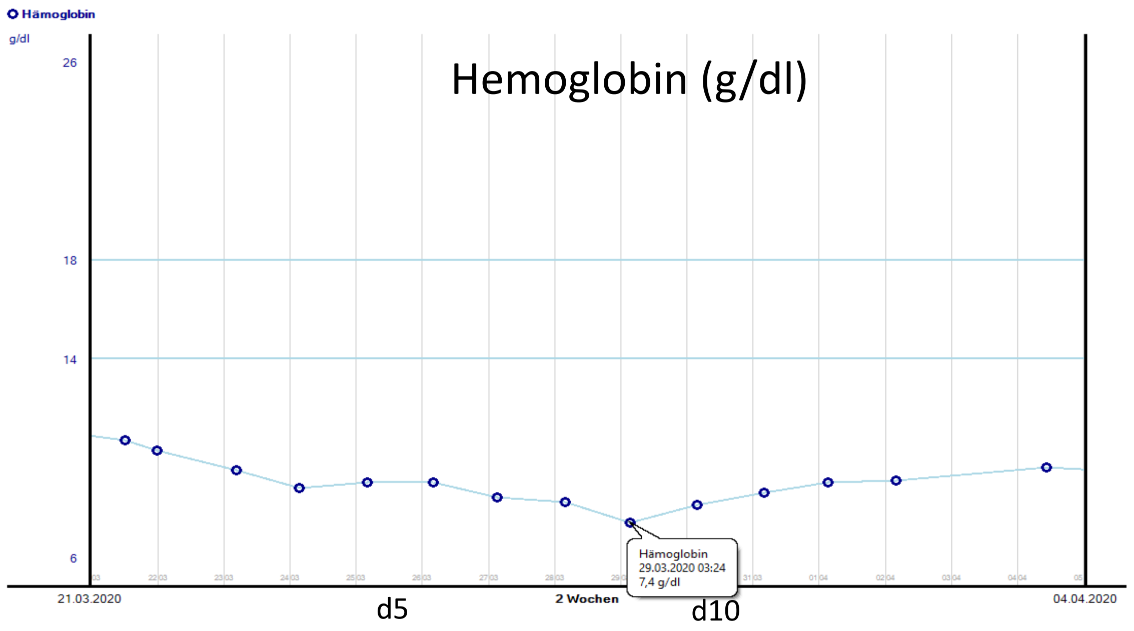

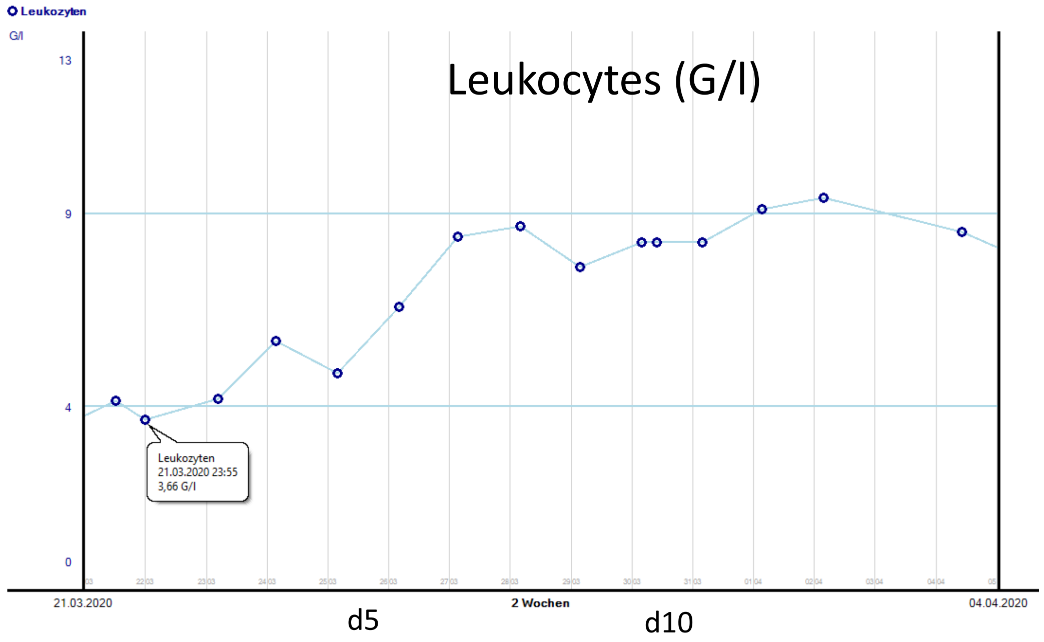

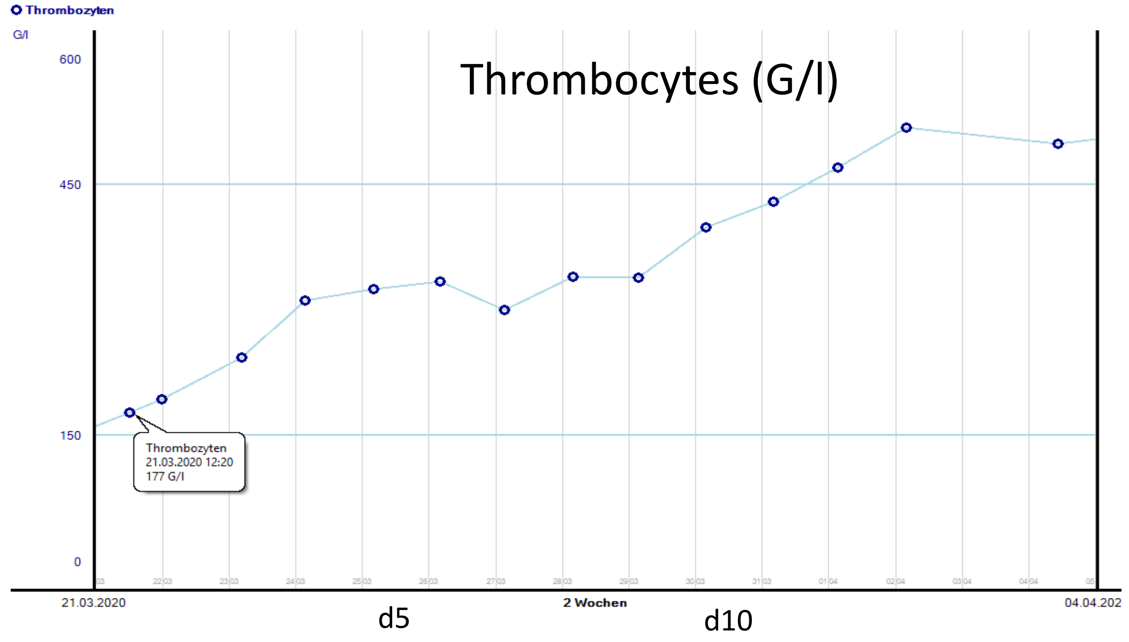

ID 13; m, 59 years

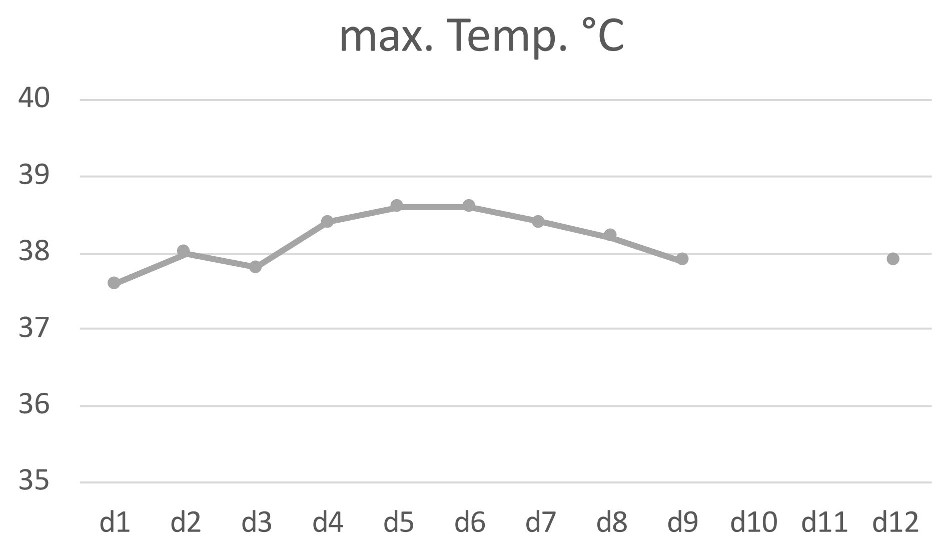

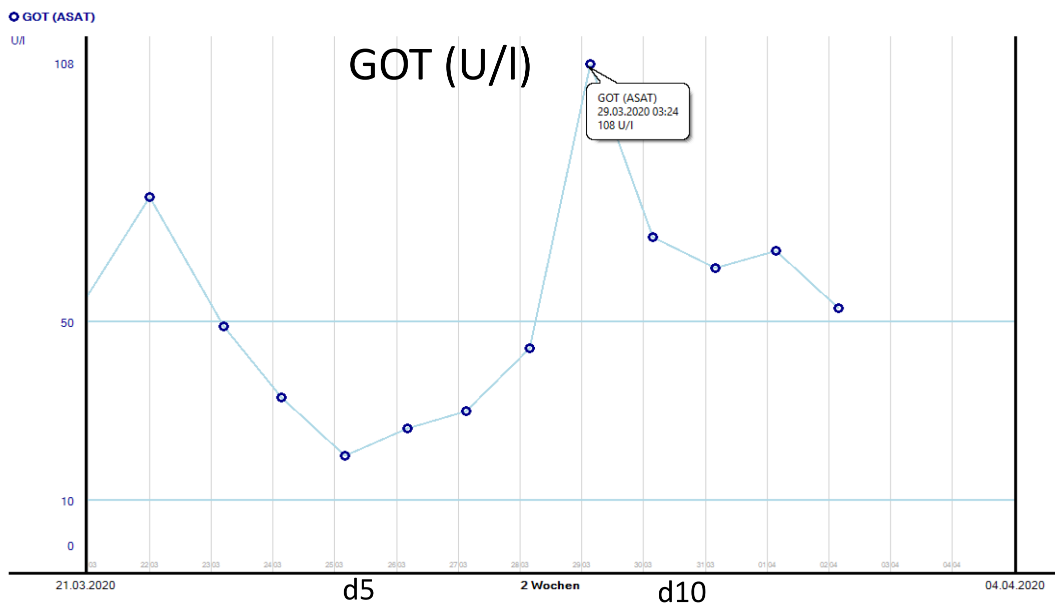

	d1	d2	d3	d4	d5	d6	d7	d8	d9	d10
Ferritin (ng/ml)			1652							1281
sIL2-R (U/ml)										1271
Fibrinogen (mg/dl)			859						838	
Triglycerides (mg/dl)			204							
INR			0.9		1.0				1.0	
D-Dimer (µg/l FEU)					5444		60630		10451	
Bilirubin (mg/dl)			0.3		0.3				0.2	
Procalcitonin (ng/ml)			0.2		0.2				0.2	

ID 14; m, 75 years

Relevant comorbidities:
Atrial fibrillation

Immunsuppression: no

Splenomegaly: unknown

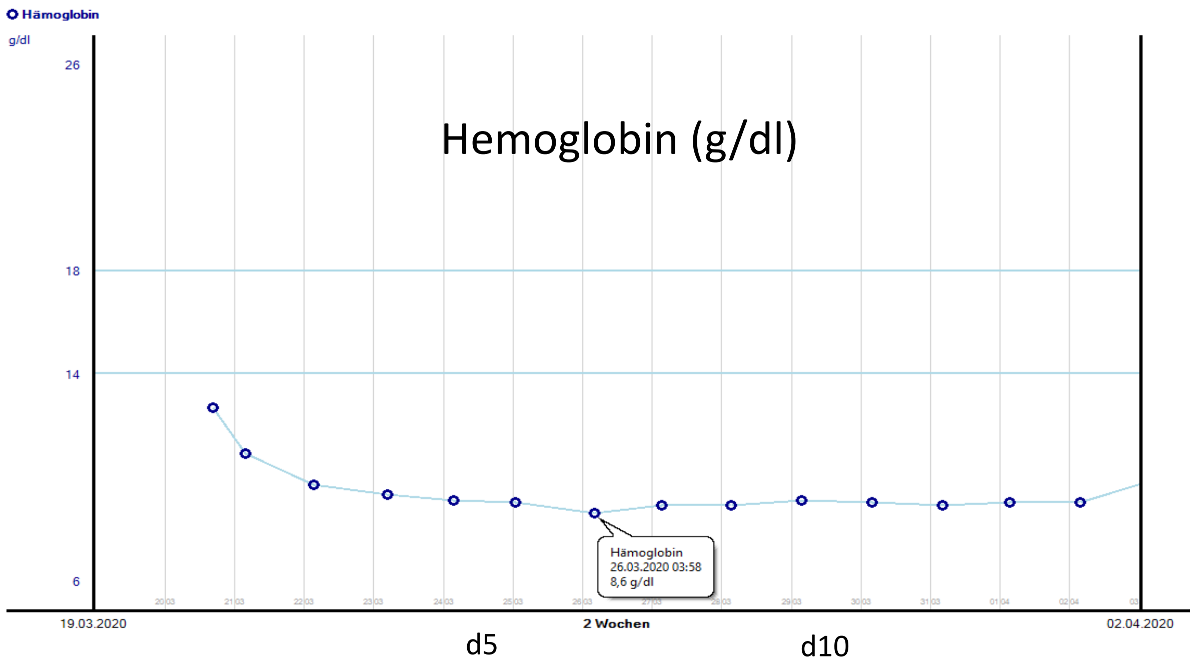

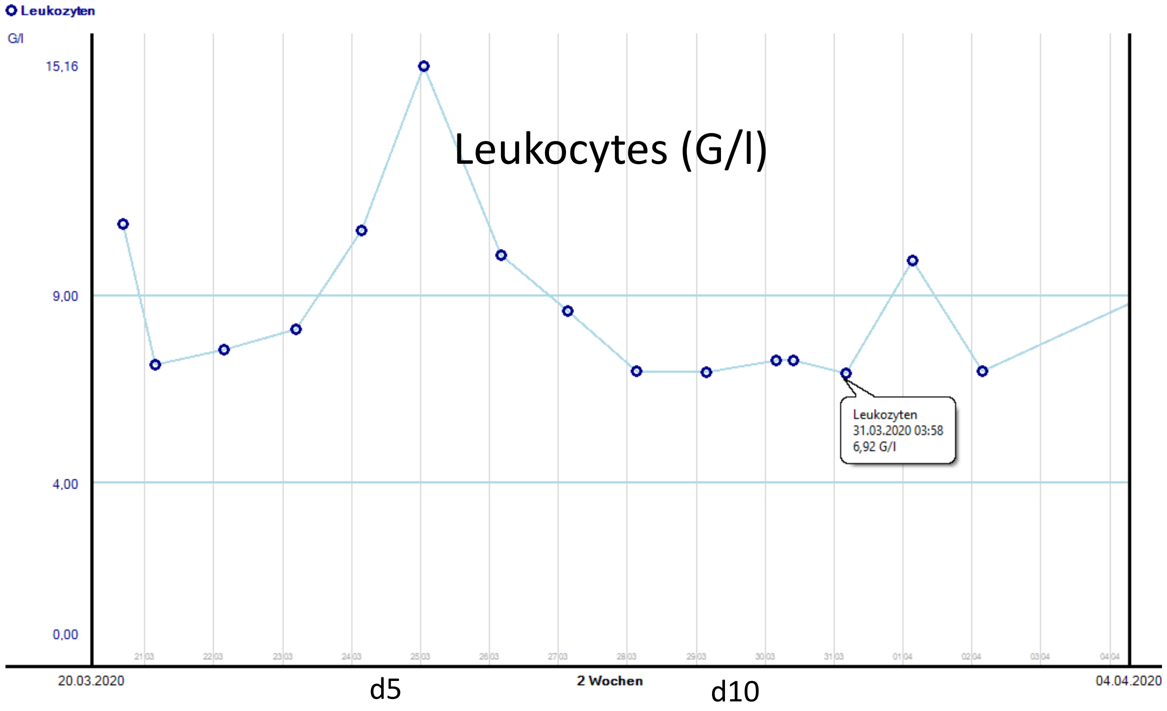

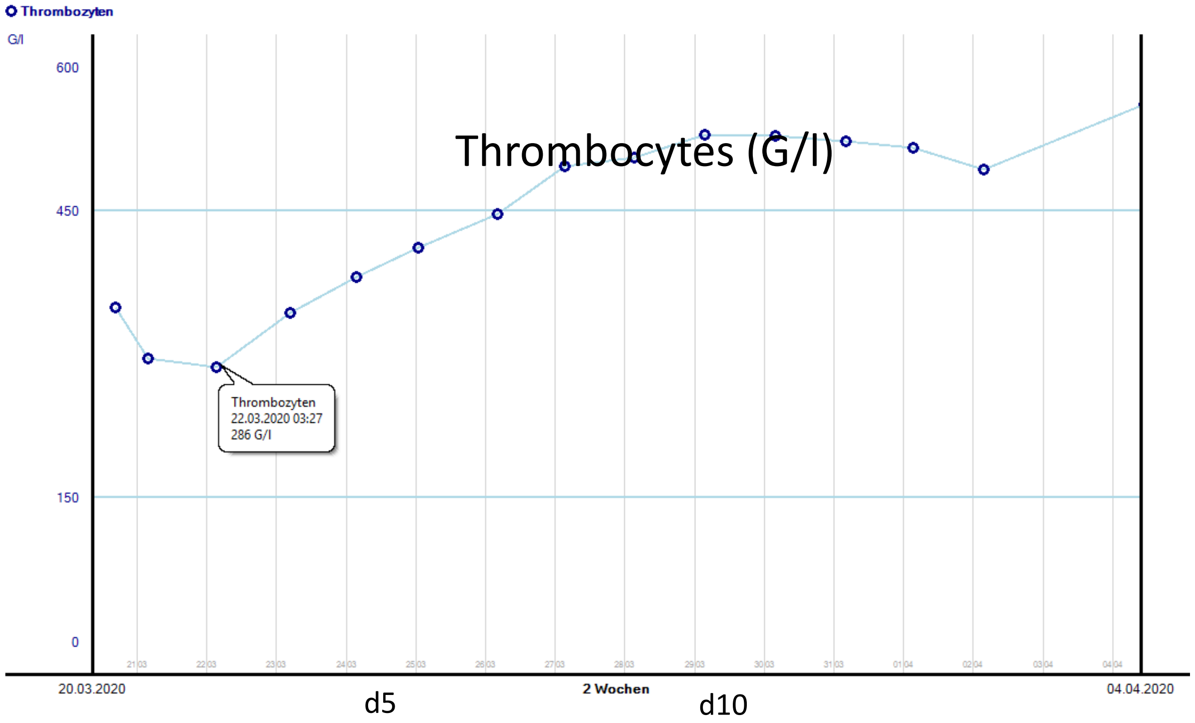

ID 14; m, 75 years

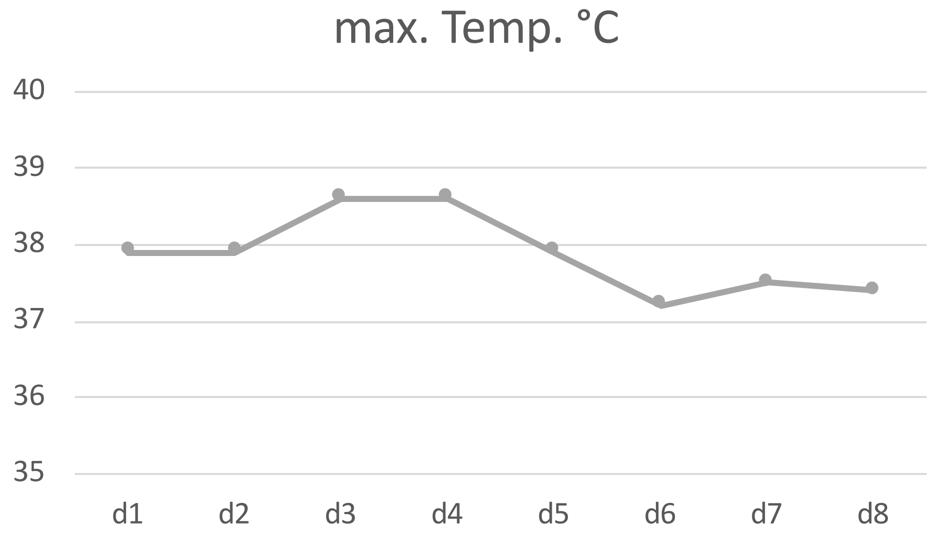

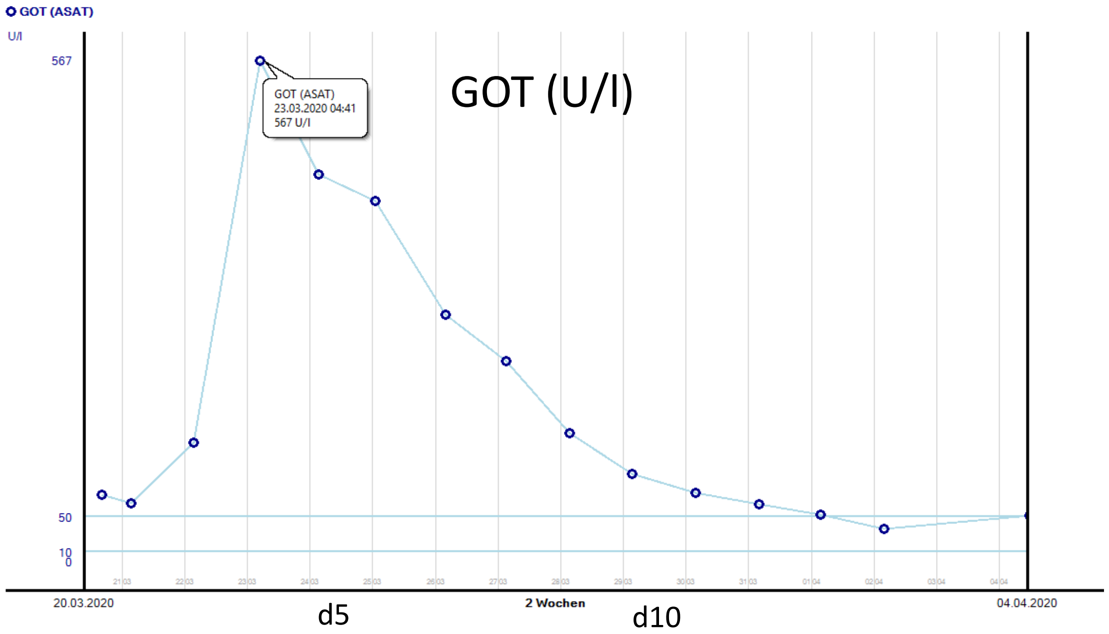

	d1	d2	d3	d4	d5	d6	d7	d8	d9	d10
Ferritin (ng/ml)							4269			1441
sIL2-R (U/ml)										1075
Fibrinogen (mg/dl)		1051						697		
Triglycerides (mg/dl)		206								
INR	1.2					1.2		1.3		
D-Dimer (µg/l FEU)						8803		6808		2912
Bilirubin (mg/dl)	1.8					1.3		0.7		
Procalcitonin (ng/ml)	5.3					0.5		0.4		

Superinfection: positive blood culture for Staph. Epid. on d1

ID 15, m, 84 years

Relevant comorbidities:

Ischemic cardiomyopathy
Arterial hypertension
Diabetes mellitus Type 2
Diabetic Chronic kidney disease
dementia

Immunosuppression: no

Splenomegaly: no

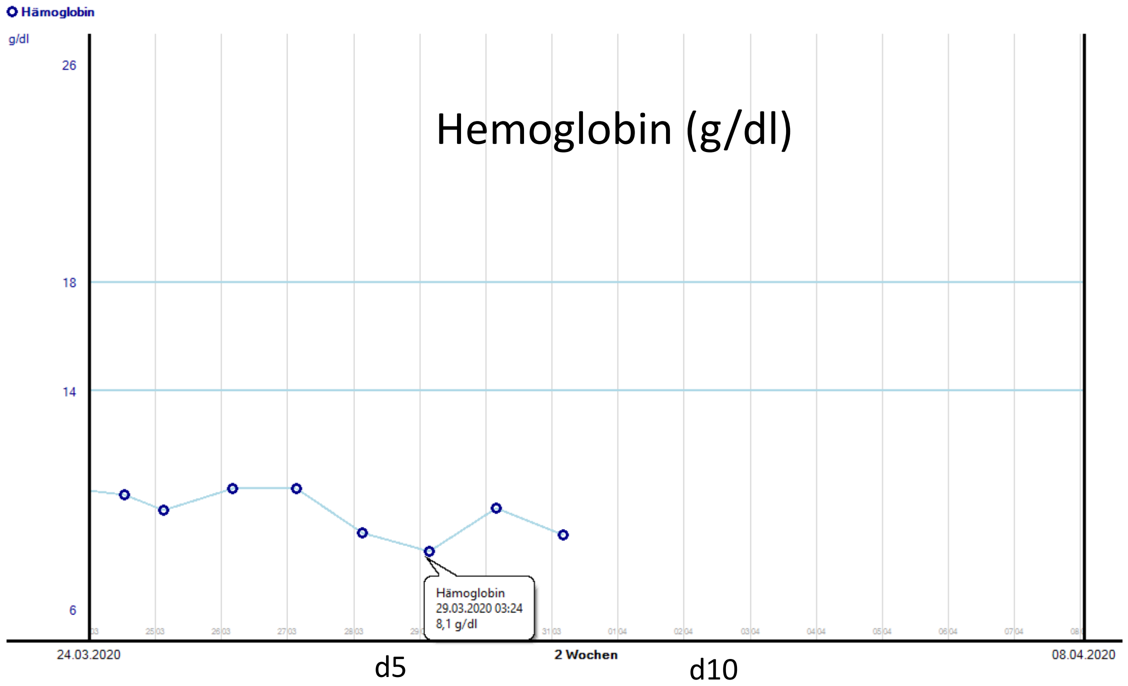

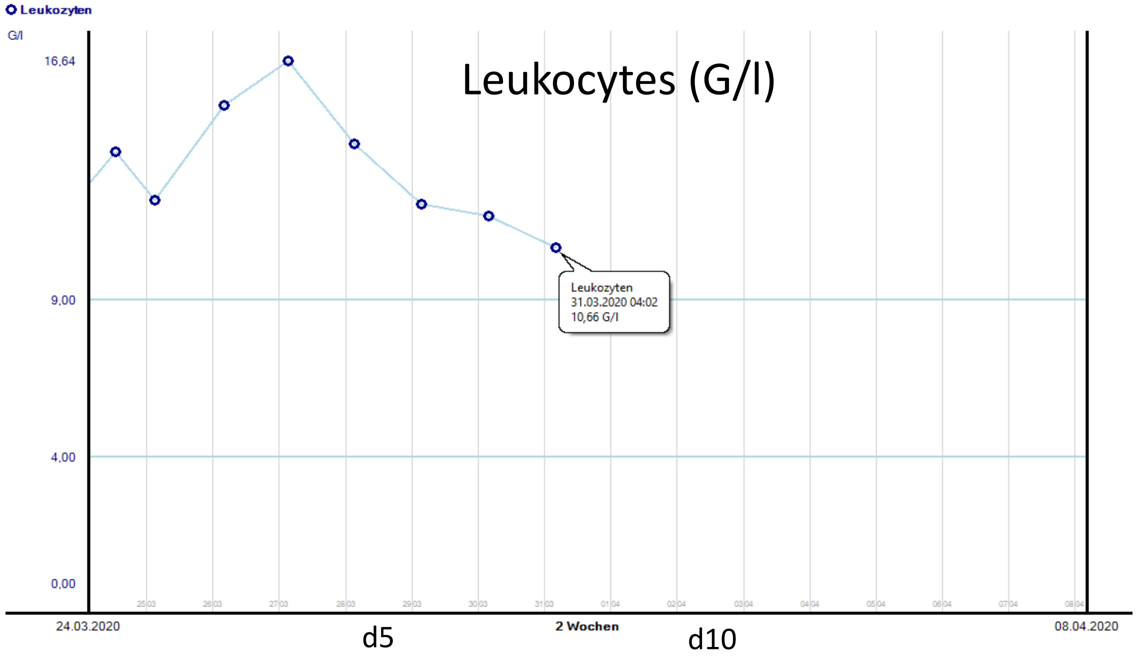

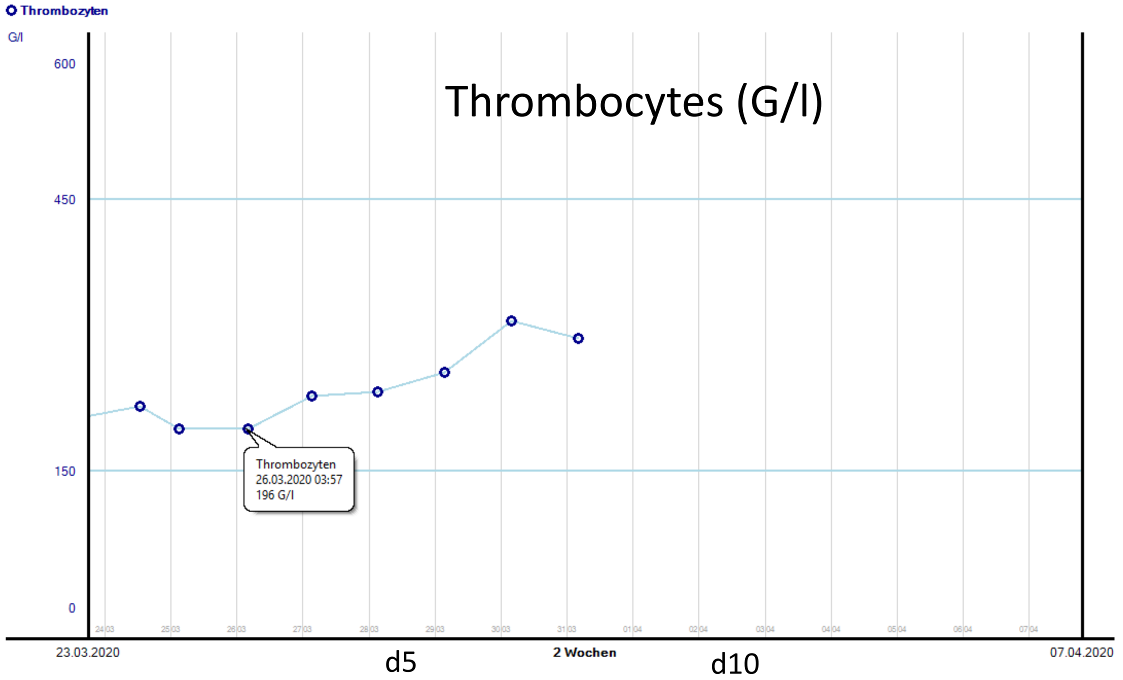

ID 15, m, 84 years

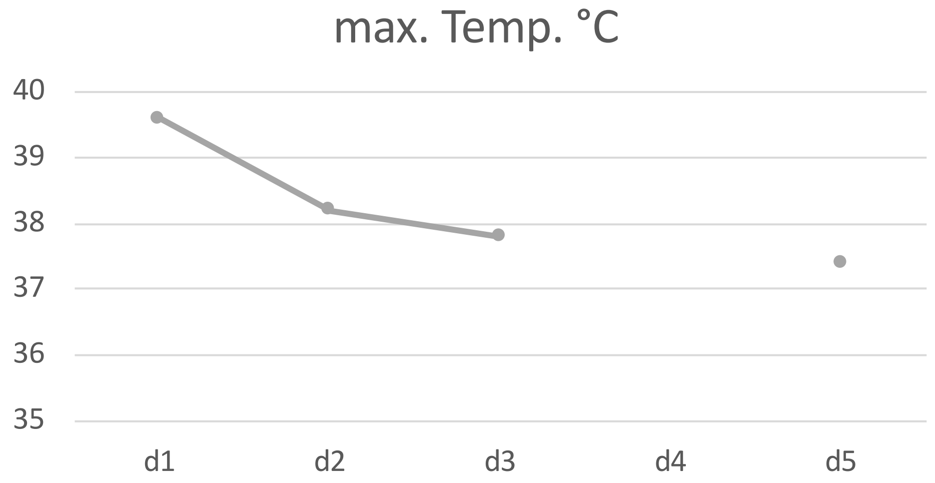

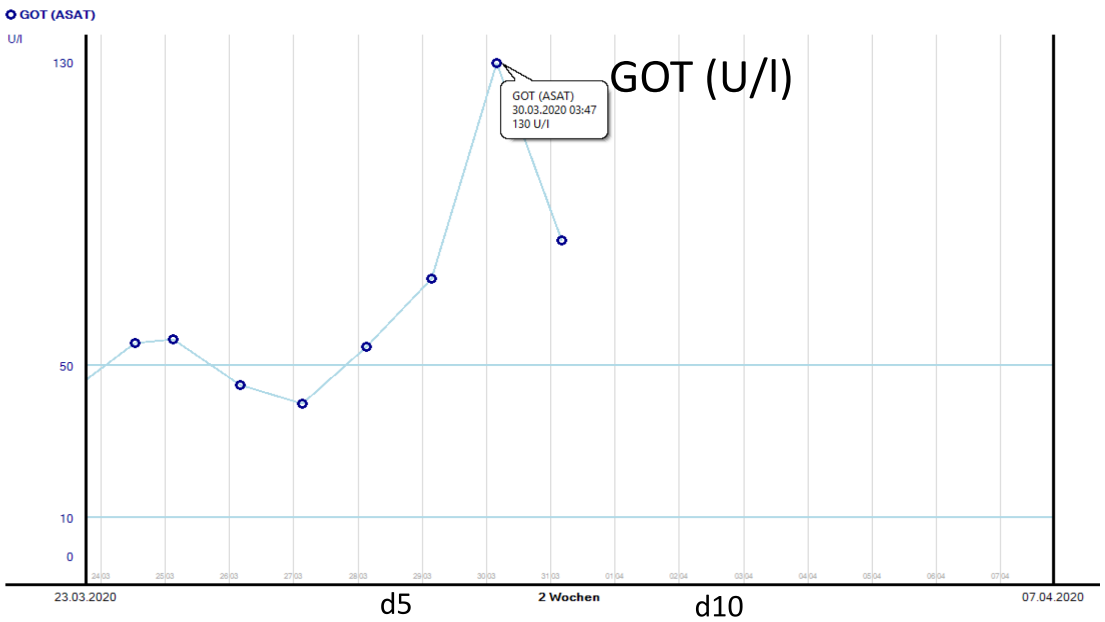

	d1	d2	d3	d4	d5	d6	d7	d8	d9	d10
Ferritin (ng/ml)				3607			1724			
sIL2-R (U/ml)										
Fibrinogen (mg/dl)				860			614			
Triglycerides (mg/dl)				57						
INR	1.0		1.0					1.1		
D-Dimer (µg/l FEU)	6333		27077				12885			
Bilirubin (mg/dl)	0.4		0.4					<0.2		
Procalcitonin (ng/ml)	1.0				2.4		0,4			

Superinfection: positive blood culture for staph. epid. on d7

ID 16; m, 80 years

Relevant comorbidities:
Lung fibrosis
Arterial hypertension
Diabetes mellitus Type 2

Immunosuppression: no

Splenomegaly: no

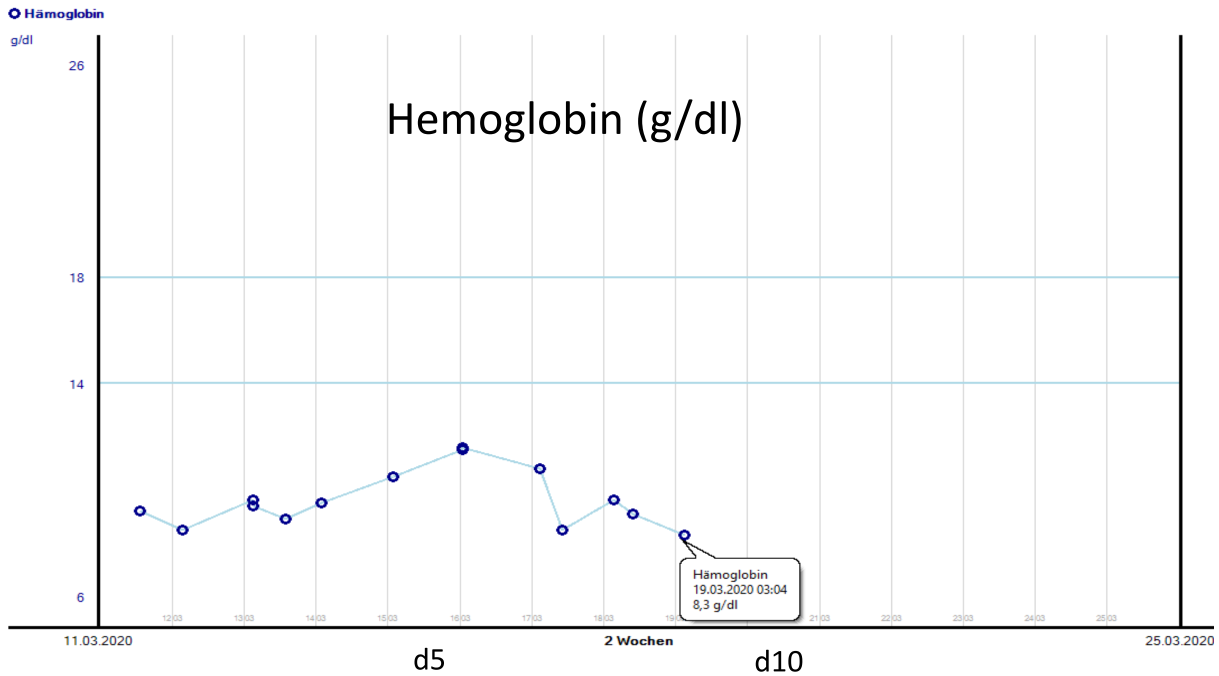

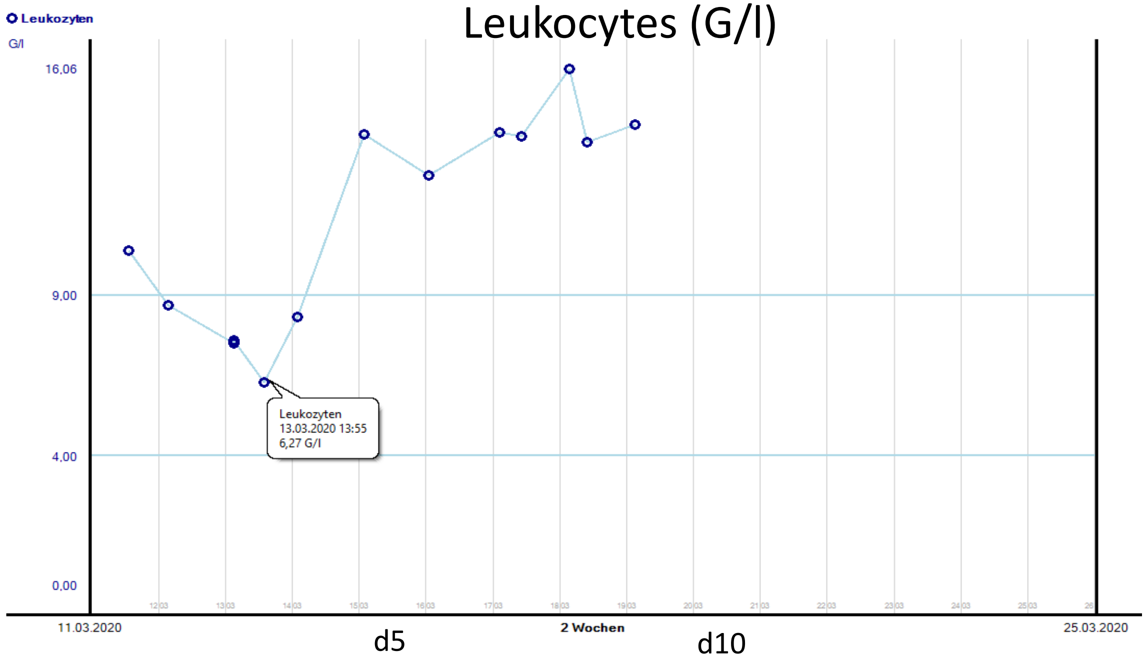

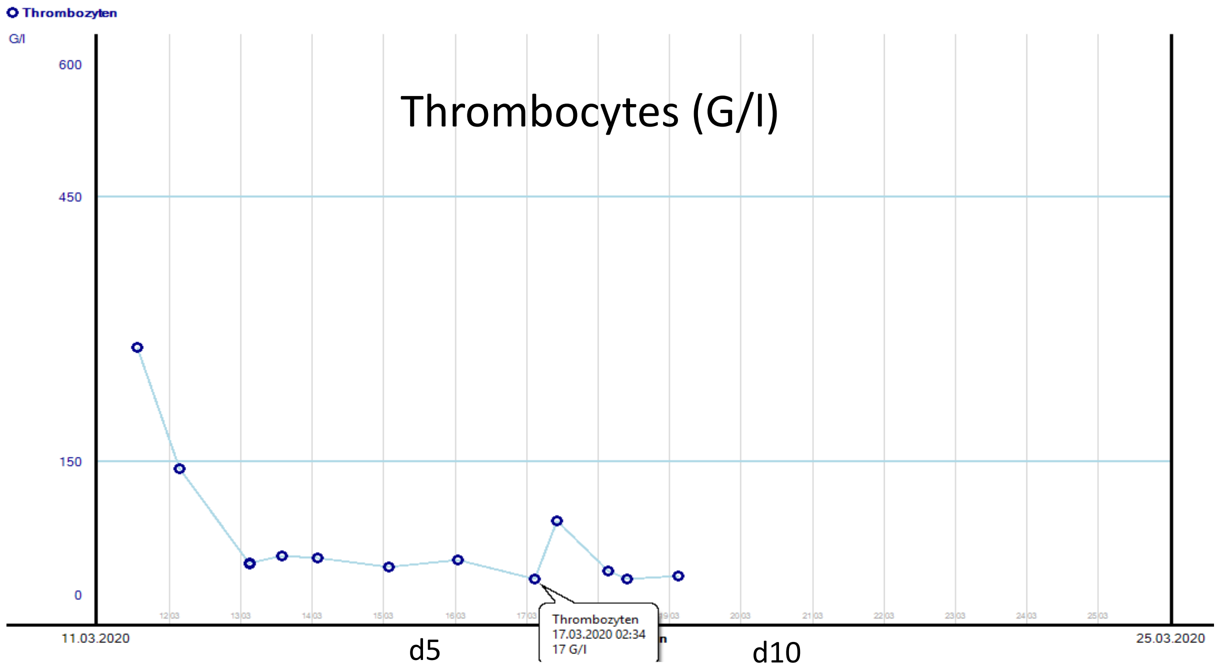

ID 16; m, 80 years

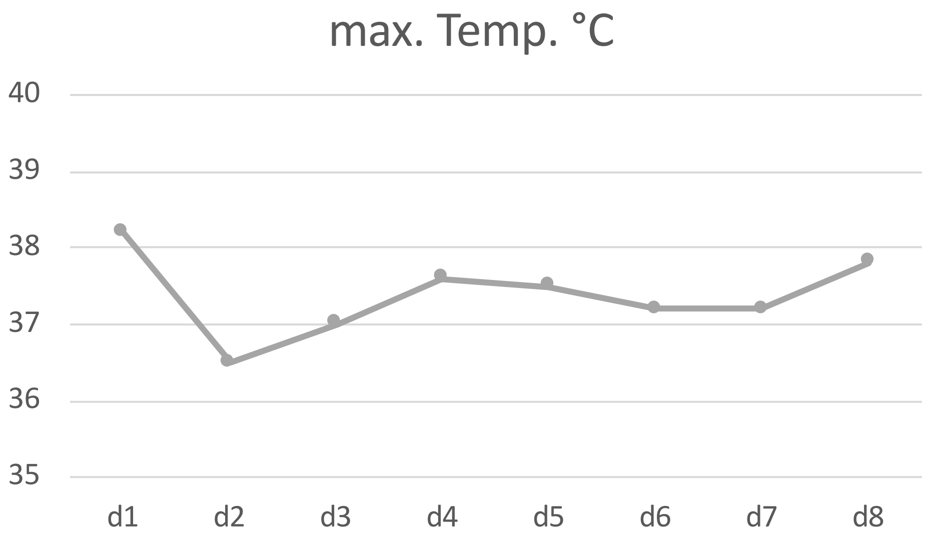

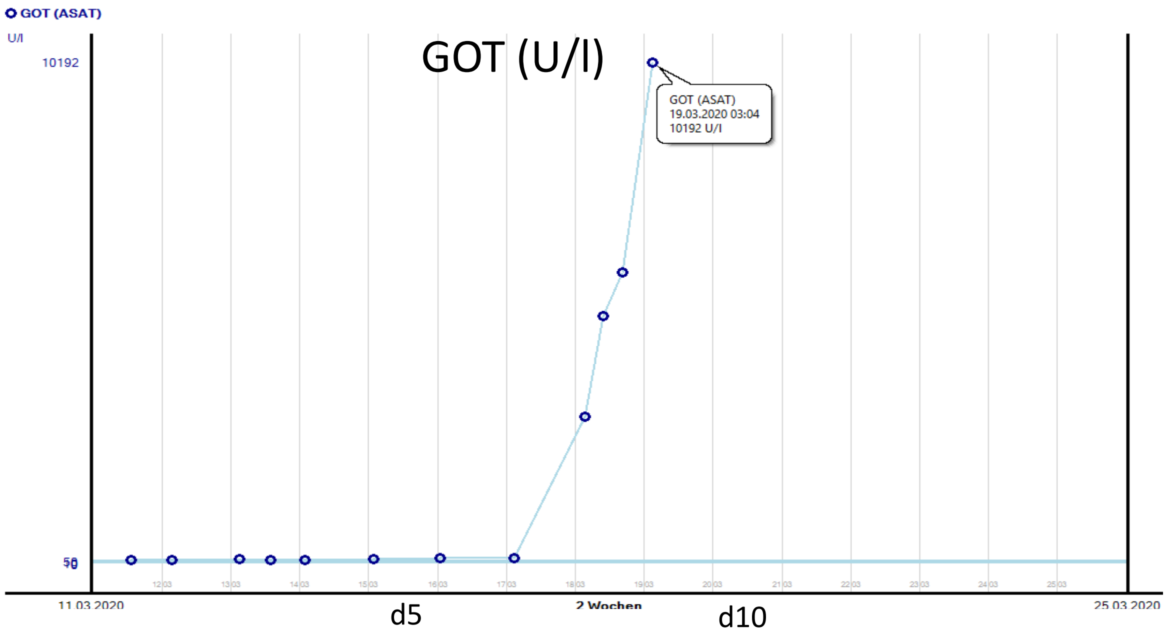

	d1	d2	d3	d4	d5	d6	d7	d8	d9	d10
Ferritin (ng/ml)									15729	
sIL2-R (U/ml)									9467	
Fibrinogen (mg/dl)				370						
Triglycerides (mg/dl)		433				454			608	
INR	1.0				1.1			4.2		
D-Dimer (µg/l FEU)		31497								
Bilirubin (mg/dl)	1.3				8.0			17.8		
Procalcitonin (ng/ml)	3.7				5.7			4.8		

Superinfection: Klebsiella oxytoca positive in tracheal secretion (d1) and blood culture (d5)

ID 17; m, 59 years

Relevant comorbidities:
Chronic Pancreatitis
Cerebrovascular insult May 2010
Chronic kidney disease
Arterial hypertension
Diabetes mellitus Type 2

Immunosuppression: no

Splenomegaly: yes

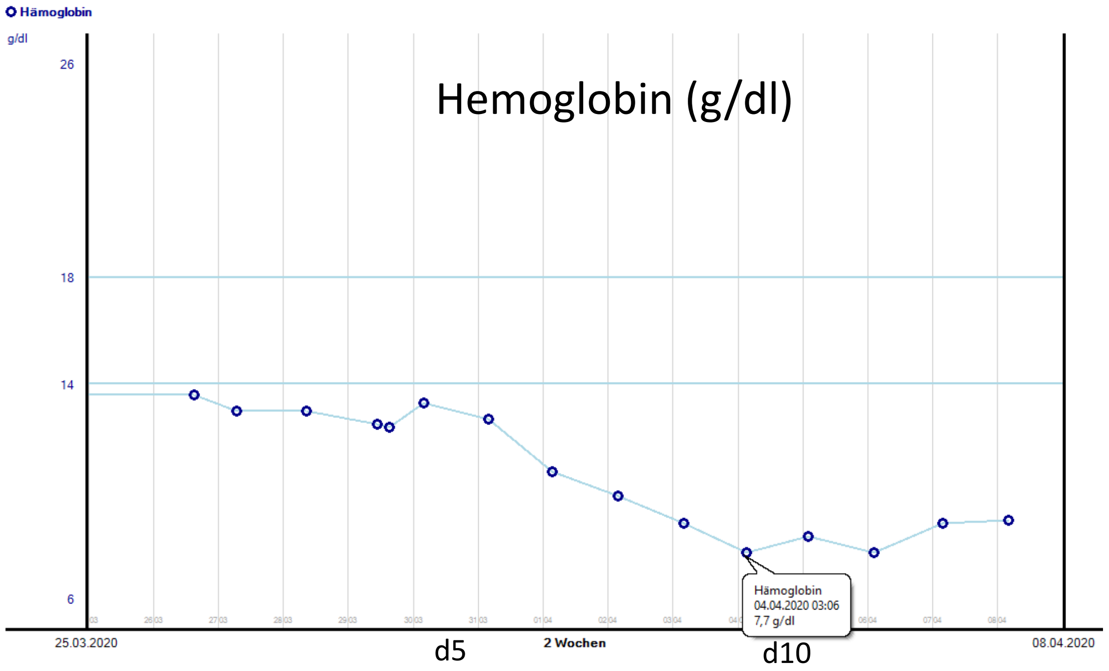

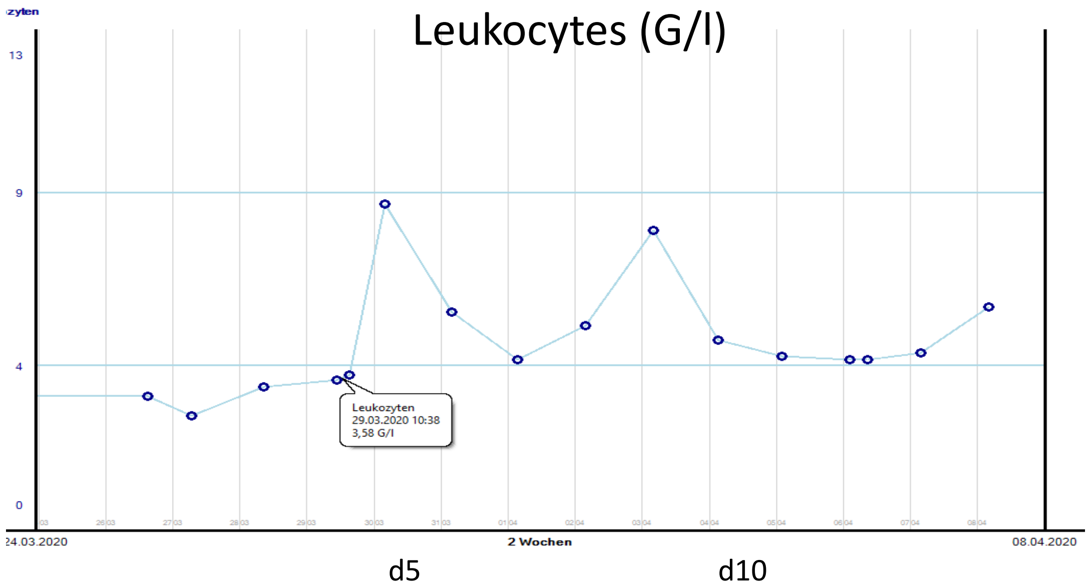

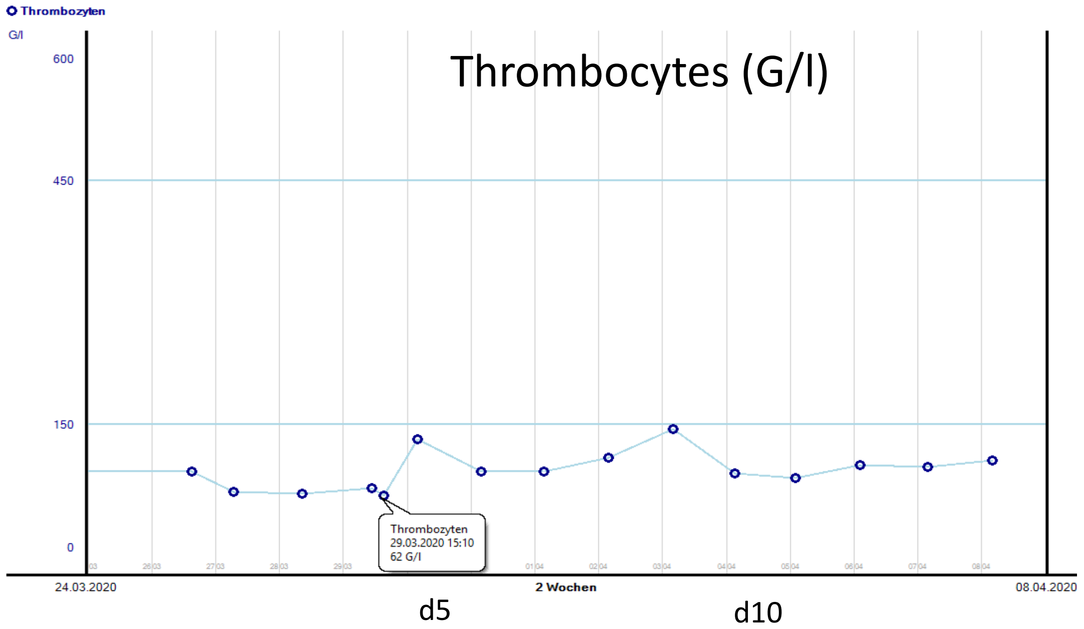

ID 17; m, 59 years

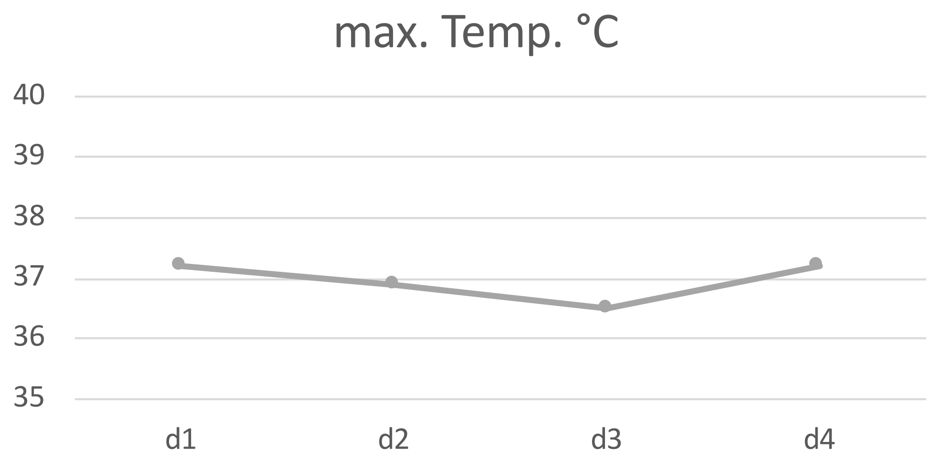

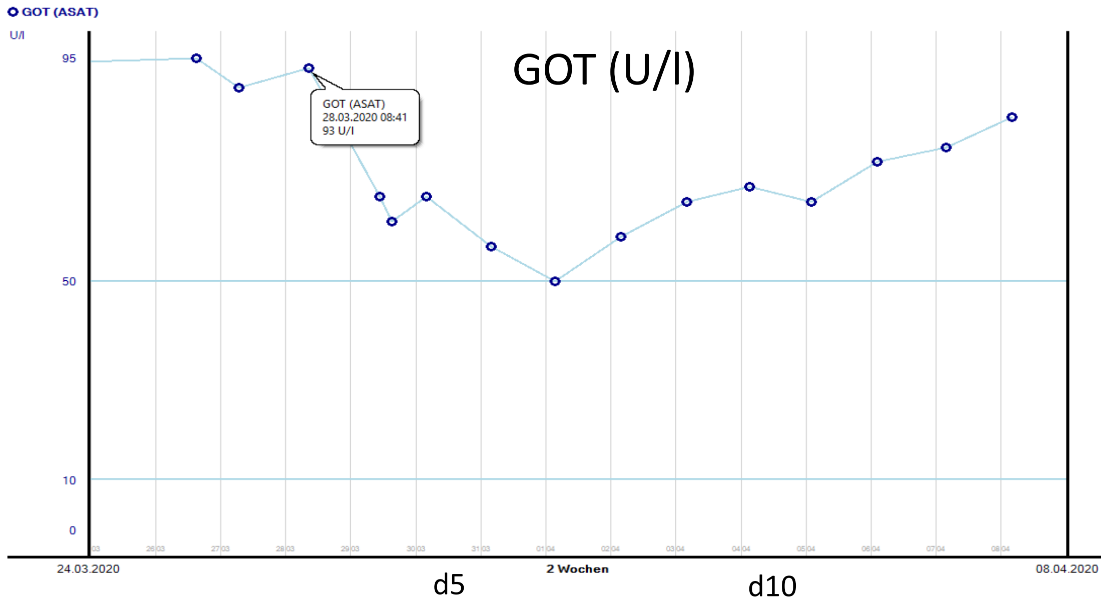

	d1	d2	d3	d4	d5	d6	d7	d8	d9	d10
Ferritin (ng/ml)	1399		1851			1818			1547	
sIL2-R (U/ml)										3091
Fibrinogen (mg/dl)	654		639			347			420	
Triglycerides (mg/dl)	298					277				
INR	0.9				0.9			1.0		
D-Dimer (µg/l FEU)	1498					3631			6492	
Bilirubin (mg/dl)	2.0				2.5			1.4		
Procalcitonin (ng/ml)	3.3				6.1			1.4		

Superinfection: culture negative

ID 18, m, 72 years

Relevant comorbidities:
COPD GOLD IV
Arterial hypertension

Immunosuppression: no

Splenomegaly: no

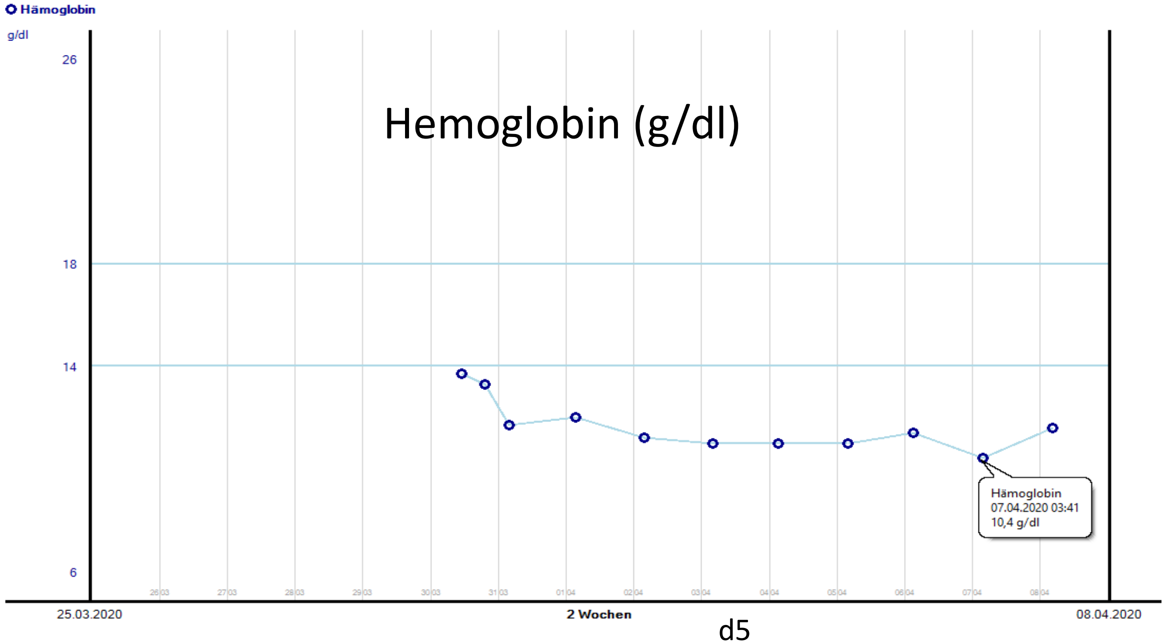

d5

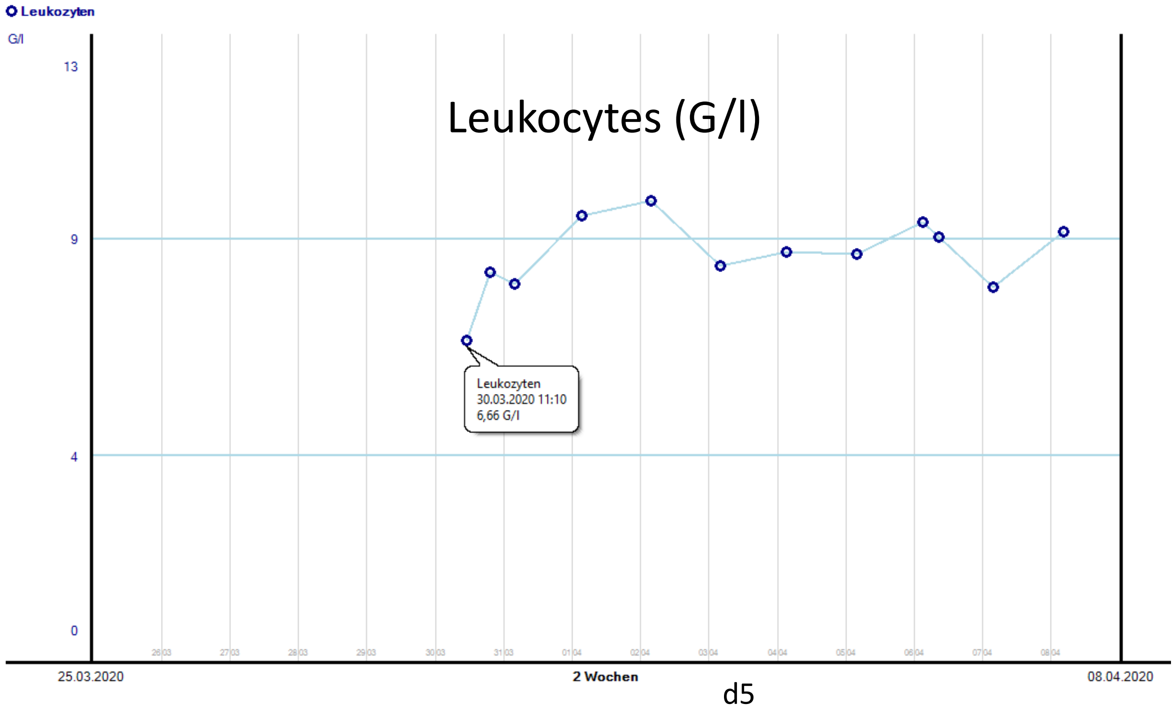

d5

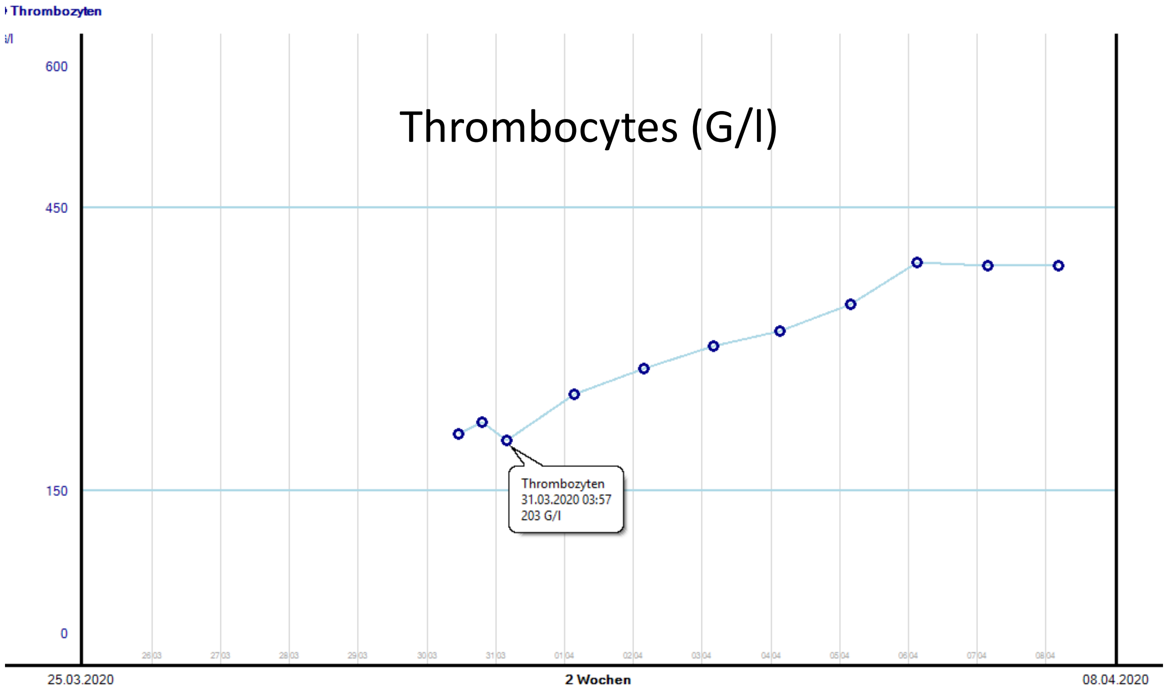

d5

ID 18, m, 72 years

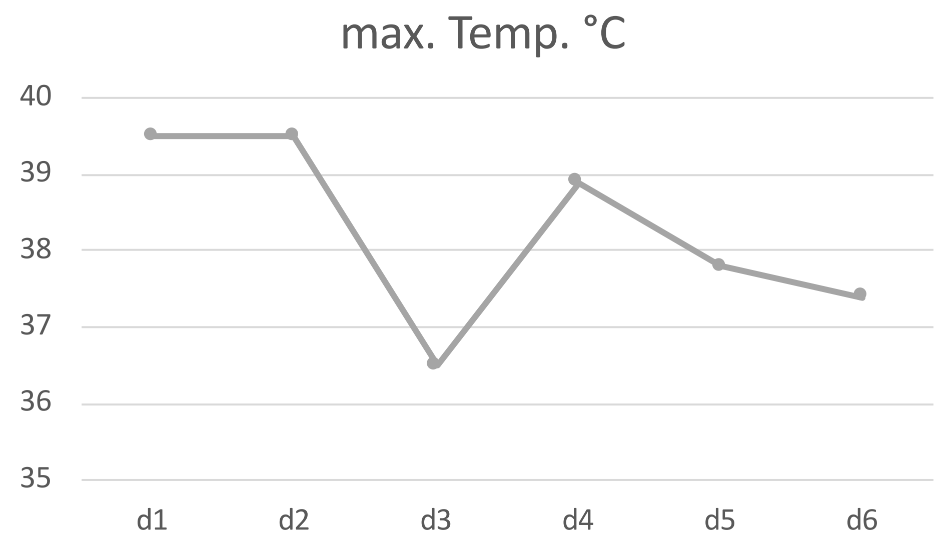

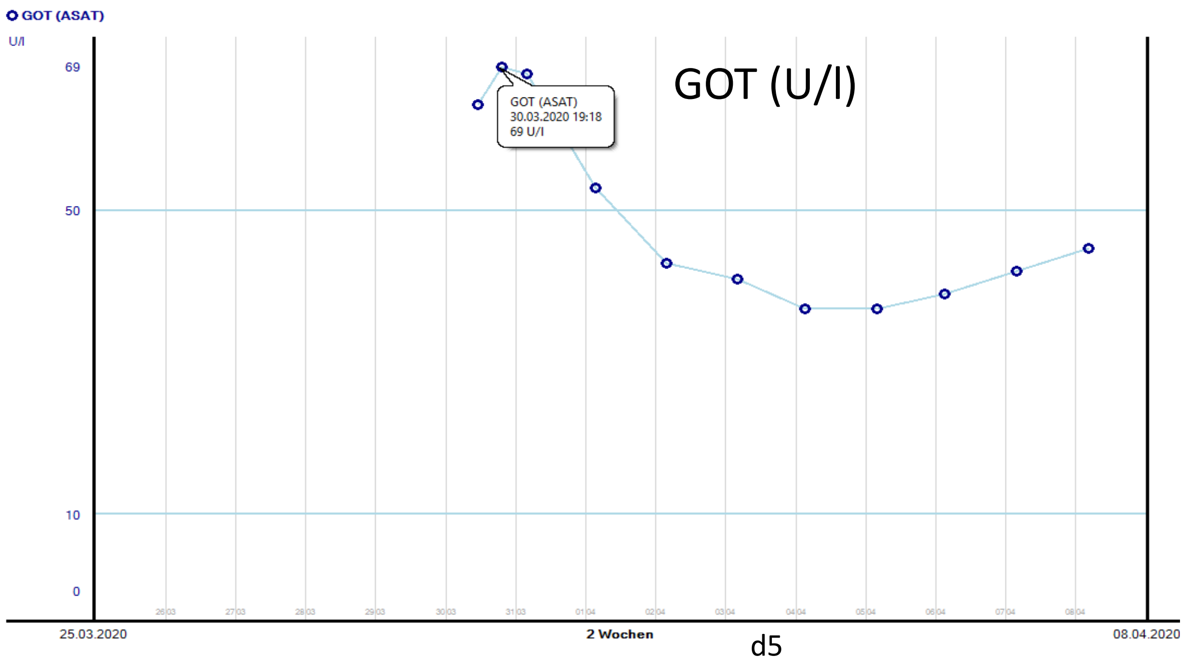

	d1	d2	d3	d4	d5	d6	d7	d8	d9	d10
Ferritin (ng/ml)	644			797					768	
sIL2-R (U/ml)							1147			
Fibrinogen (mg/dl)	793			619					639	
Triglycerides (mg/dl)	97									
INR	0.9				0.9			1.2		
D-Dimer (µg/l FEU)	1141			8310				3334		
Bilirubin (mg/dl)	0.6				0.4			0.4		
Procalcitonin (ng/ml)	0.5			0.2			0.1			

ID 19; m, 68 years

Relevant comorbidities:
Arterial hypertension
Vascular Dementia

Immunosuppression: no

Splenomegaly: no

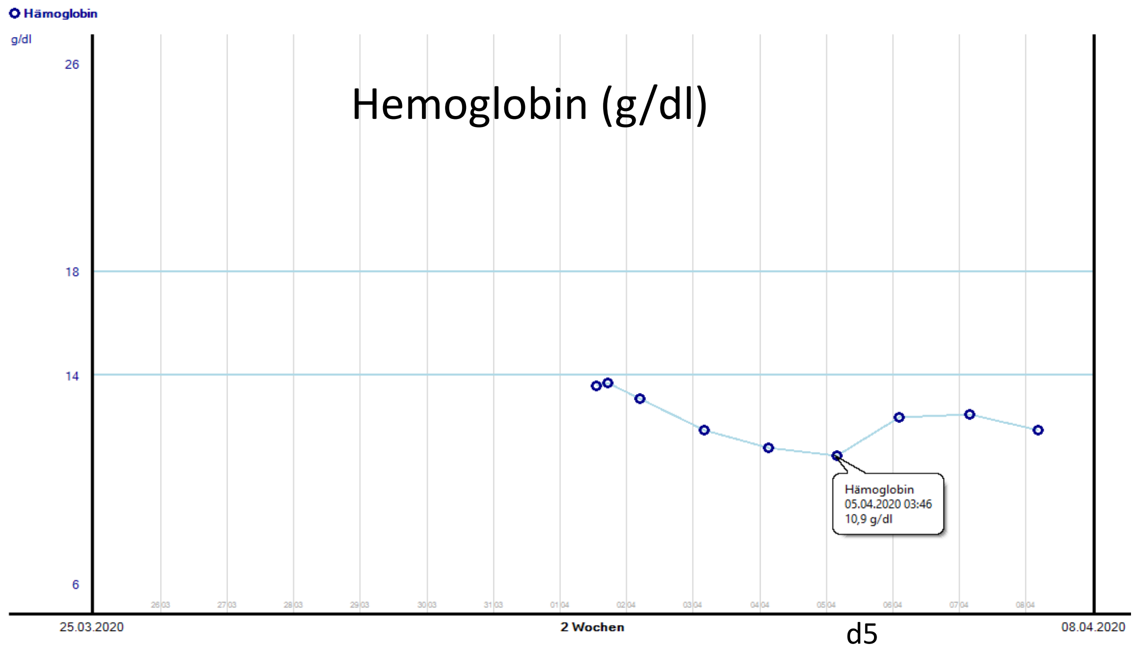

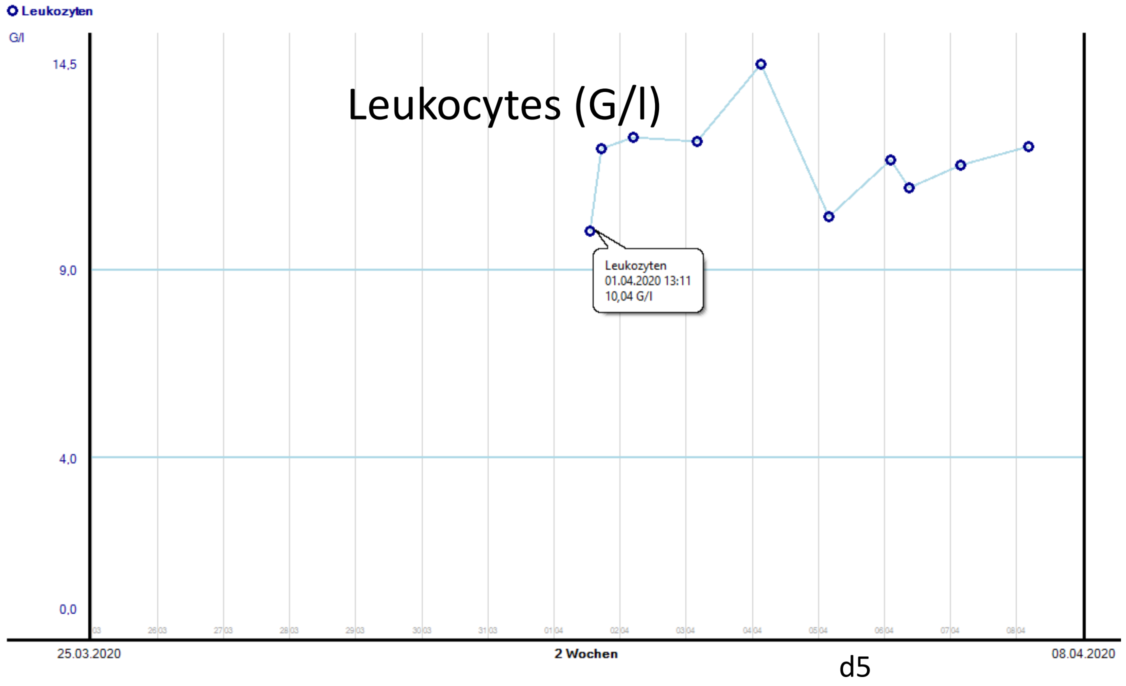

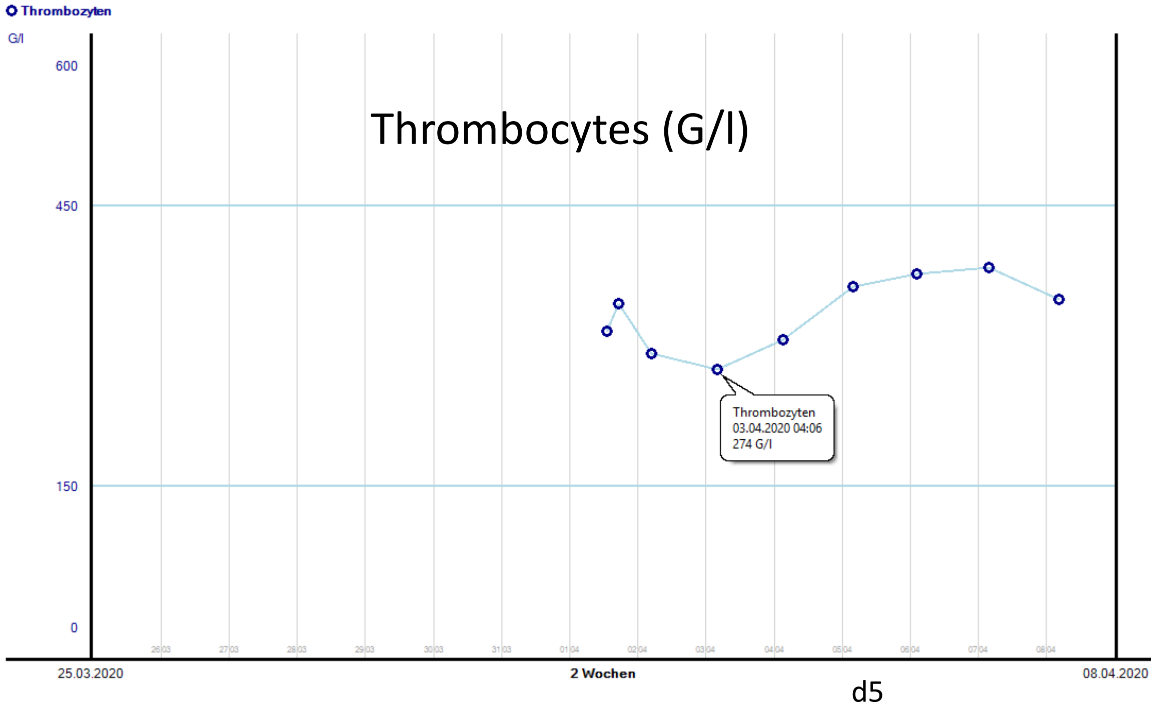

ID 19; m, 68 years

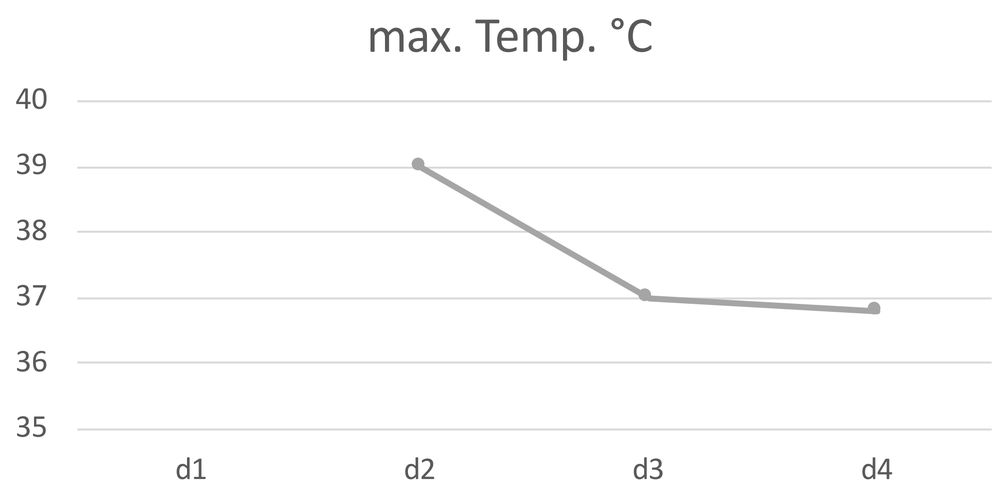

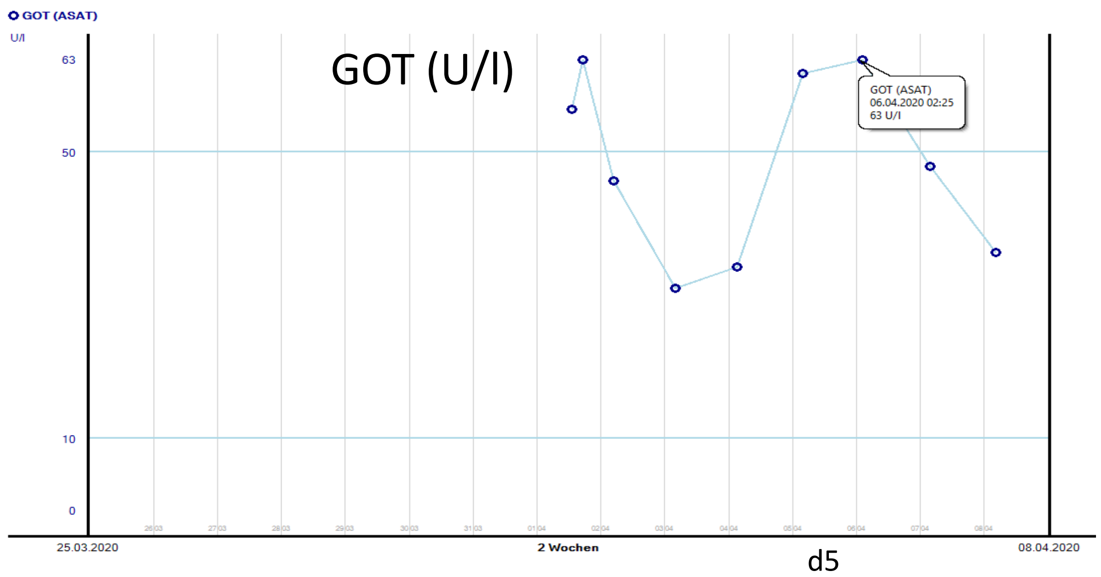

	d1	d2	d3	d4	d5	d6	d7	d8	d9	d10
Ferritin (ng/ml)	2700				1689		882			
sIL2-R (U/ml)						447				
Fibrinogen (mg/dl)	772				418		519			
Triglycerides (mg/dl)	206									
INR	0,9				0.9			0.9		
D-Dimer (µg/l FEU)	31990				6015			3721		
Bilirubin (mg/dl)	1.2				0.5			0.4		
Procalcitonin (ng/ml)	0.2				0.2		0.1			
